# Systematic analysis of the scientific-technological production on the use of the UV, H_2_O_2_, and/or Cl_2_ systems in the elimination of bacteria and associated antibiotic resistance genes

**DOI:** 10.1007/s11356-023-31435-2

**Published:** 2024-01-02

**Authors:** Paula Andrea Espinosa-Barrera, Marcela Gómez-Gómez, Javier Vanegas, Fiderman Machuca-Martinez, Ricardo Antonio Torres-Palma, Diana Martínez-Pachón, Alejandro Moncayo-Lasso

**Affiliations:** 1 Grupo de Investigación en Ciencias Biológicas y Químicas, Facultad de Ciencias, Universidad Antonio Nariño, Bogotá D.C., Colombia; 2 Doctorado en Ciencia Aplicada (DCA), Universidad Antonio Nariño, Bogotá D.C., Colombia; 3 Centro de Excelencia en Nuevos Materiales, Universidad del Valle, Calle 13 No. 100-00, Cali, Colombia; 4 Grupo de Investigación en Remediación Ambiental y Biocatálisis (GIRAB), Instituto de Química, Facultad de Ciencias Exactas y Naturales, Universidad de Antioquia UdeA, Calle 70 No. 52-21, Medellín, Colombia

**Keywords:** Disinfection, Chlorine, Hydrogen peroxide, Ultraviolet, Water treatment, Advanced oxidation process

## Abstract

This study presents a systematic review of the scientific and technological production related to the use of systems based on UV, H_2_O_2_, and Cl_2_ for the elimination of antibiotic-resistant bacteria (ARB) and genes associated with antibiotic resistance (ARGs). Using the Pro Know-C (Knowledge Development Process-Constructivist) methodology, a portfolio was created and analyzed that includes 19 articles and 18 patents published between 2011 and 2022. The results show a greater scientific-technological production in UV irradiation systems (8 articles and 5 patents) and the binary combination UV/H_2_O_2_ (9 articles and 4 patents). It was emphasized that UV irradiation alone focuses mainly on the removal of ARB, while the addition of H_2_O_2_ or Cl_2_, either individually or in binary combinations with UV, enhances the removal of ARB and ARG. The need for further research on the UV/H_2_O_2_/Cl_2_ system is emphasized, as gaps in the scientific-technological production of this system (0 articles and 2 patents), especially in its electrochemically assisted implementation, have been identified. Despite the gaps identified, there are promising prospects for the use of combined electrochemically assisted UV/H_2_O_2_/Cl_2_ disinfection systems. This is demonstrated by the effective removal of a wide range of contaminants, including ARB, fungi, and viruses, as well as microorganisms resistant to conventional disinfectants, while reducing the formation of toxic by-products.

**Supplementary Information:**

The online version contains supplementary material available at 10.1007/s11356-023-31435-2.

## Introduction

The overuse and inadequate degradation of antibiotics by the organisms that consume them leads to their continuous release into the environment, particularly into aquatic ecosystems. This release, combined with the great diversity of bacteria in wastewater, contributes to the increased occurrence and spread of antibiotic-resistant bacteria (ARB), which poses a risk to both human health and the environment due to the decreasing effectiveness of antibiotics in the treatment of infectious diseases (Anand et al. 2021; Fiorentino et al. 2015; Sharma et al. 2016).

Antibiotic resistance is transmitted through specific genes that can be shared or exchanged between bacteria via horizontal gene transfer processes such as transformation, transduction, and conjugation (Yoon et al. 2017). Currently, ARBs and their antibiotic resistance genes (ARGs) are considered as a biological contaminant of emerging concern (CEC) in water systems, especially in wastewater, due to the limitations of conventional treatment processes that only partially remove ARBs and ARGs (Anand et al. 2021; Fiorentino et al. 2015; Koch et al. 2021). This problem has reached significant scale, prompting the World Health Organization (WHO) to declare that antimicrobial resistance is one of the 10 threats to public health at a global level, with critical implications for health and development. Mitigating this challenge requires the implementation of urgent cross-sectoral action to archieve the sustainable development goals (SDGs) (World Health Organization (WHO), 2021; Yadav and Kapley 2021).

While advanced oxidation processes (AOPs) such as Cl_2_, O_3_, UV, and UV/H_2_O_2_ have been investigated for the removal of ARBs and ARGs in wastewater at laboratory, pilot, and large scale (Fiorentino et al. 2015; Yoon et al. 2017), shortcomings in wastewater disinfection continue to be identified. These deficiencies may depend on several factors, including the presence of ARGs in extracellular or intracellular DNA, the degree of elimination of ARGs based on the analytical methods used for monitoring (e.g., qPCR), system operating conditions, and water quality (Phattarapattamawong et al. 2021; Yoon et al. 2017).

To overcome the limitations of conventional AOPs, modifications and improvements have been introduced, such as the application of electrical energy to promote the electrochemical generation of oxidizing agents such as H_2_O_2_, Cl_2_, and O_3_, among others. This approach has led to electrochemical advanced oxidation processes (EAOPs) (Chaplin 2019; Yuan et al. 2023). In addition, the enhancement of these EAOPs by irradiation with ultraviolet radiation has been investigated with the aim of increasing the in situ generation of these oxidizing species, including others such as chlorine radicals (Cl∙, Cl^−^_2_∙, and ClO∙) (Wang et al. 2020; Yin et al. 2021) and singlet oxygen (^1^O_2_) (García-Fresnadillo 2018; Santos et al. 2012). This approach not only reduces reagent consumption and transportation, but also improves treatment efficiency by enabling faster elimination of CECs (Yin et al. 2021; Zhang et al. 2022) such as bacteria and ARGs (Liu and Hu 2020; Wu et al. 2021).

However, EAOP systems are still in the research and study phase, as most articles and accepted patents in recent years have only focused on evaluating the systems individually (UV, H_2_O_2_, or Cl_2_), or in binary combinations of UV/Cl_2_ (Phattarapattamawong et al. 2021), and/or UV/H_2_O_2_ systems (Ferro et al. 2017), but the use of an EAOP system combining UV, Cl_2_, and H_2_O_2_ is not considered in the literature, and even less with the aim of eliminating ARB and/or ARGs. Therefore, the aim of this study is to conduct a systematic analysis of the scientific-technological production related to the use of UV, H_2_O_2_, and/or Cl_2_ systems in the elimination of ARBs and ARGs to provide an understanding of the limitations or advantages of the system use, application trends, operating conditions, and system performance, especially in the elimination of this CECs under controlled conditions at laboratory scale in ultrapure, distilled, synthetic waters, wastewater, among others. To achieve this, the Pro Know-C (Knowledge Development Process-Constructivist) methodology (Costa et al. 2021; Linhares et al. 2019; Sanabria et al. 2022) is used to organize a bibliographic portfolio of relevant manuscripts and accepted patents on the topic of interest. The academic relevance of the scientific journals and authors was considered. Peer-reviewed articles and accepted patents published in the last 10 years (2011–2022) were included, and a set of guiding criteria on the topic was developed for the systematic analysis.

## Methodology

### Portfolio selection

**Fig. 1:**
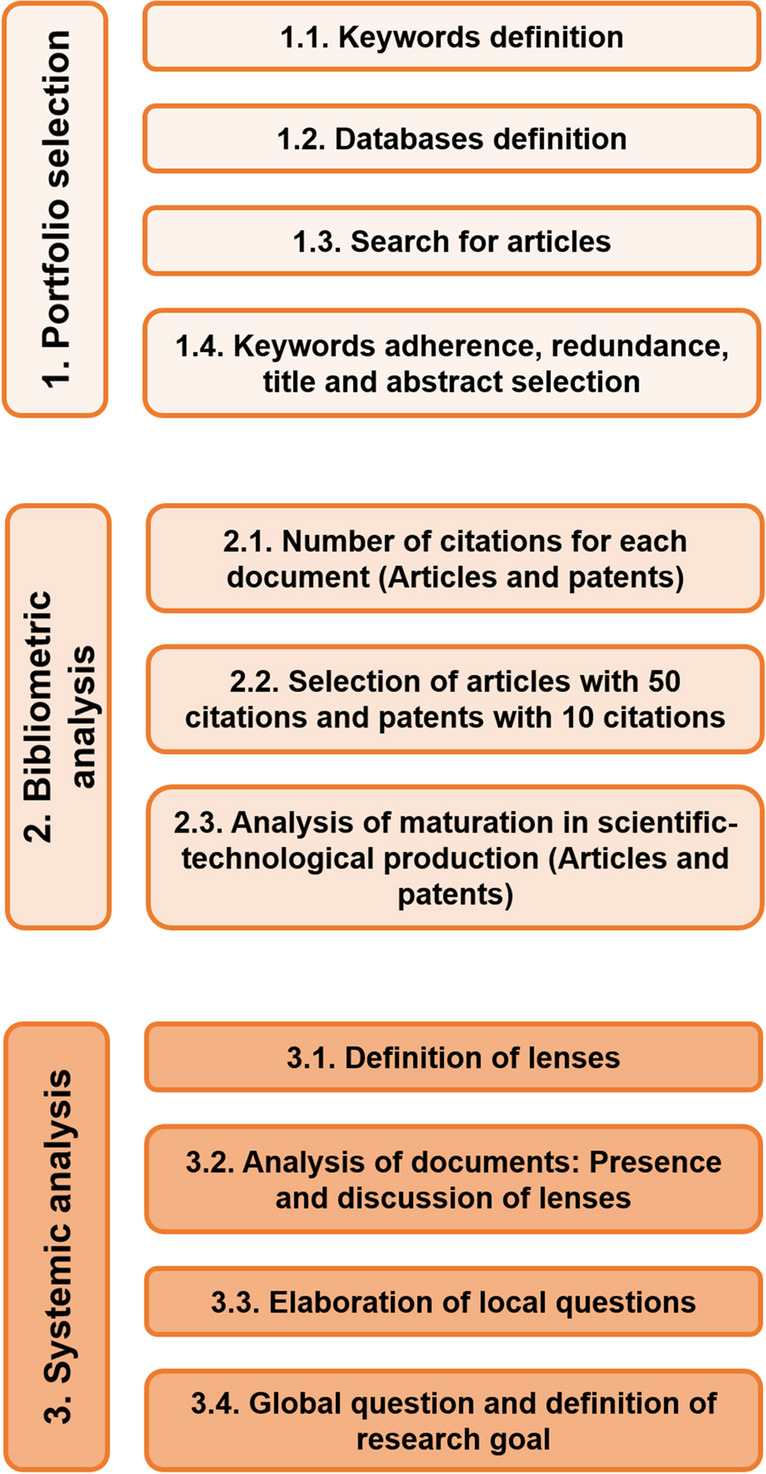
Modified Pro Know-C methodology applied to the review of scientific-technological production

A combined study of bibliometric analysis and systematic literature review was used to identify the latest scientific developments related to the use of an AOP based on UV, H_2_O_2_, and/or Cl_2_ for the elimination of ARBs and ARGs in the last decade (2011–2022). The applied methodology was based on a modified Pro Know-C approach that generated initial knowledge about the AOPs. This study allowed the collection of information on the published material and facilitated the formulation of research questions through a bibliographic portfolio of manuscripts, bibliometric analysis, and a systemic analysis (Fig. 1) (Costa et al. 2021; Linhares et al. 2019; Sanabria et al. 2022). The databases used in this study were selected for their relevance in the field of environmental sciences: ACS Journals Search, Science Direct (Elsevier), Springer Link, and Wiley Online Library.

**Fig. 2:**
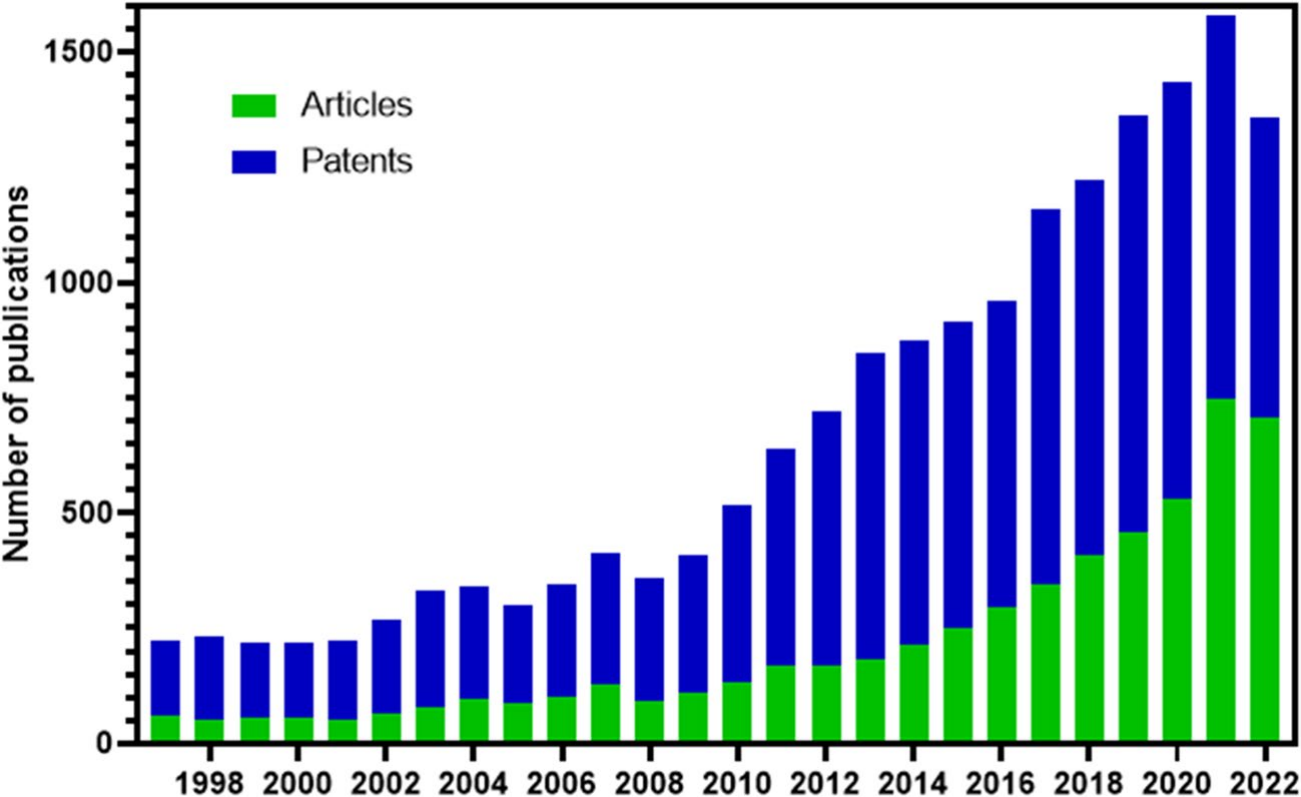
Number of publications per year between 1997 and 2022 using keyword searches ((“bacteria”) AND (“chlorine” OR “Chlorination” OR “Cl”) AND (“H_2_O_2_” OR “peroxide”) AND (“Ultraviolet” OR “UV”)) in the Science Direct database for articles and The Lens for patents. Unrefined

A preliminary search was conducted using different combinations of terms, such as “wastewater treatment,” “disinfection,” “chlorine,” “gene,” “bacteria,” “ultraviolet,” “hydrogen peroxide,” or some variant thereof (Figure SM1). This resulted to a preliminary search for manuscripts published in scientific journals in recent years (review + research) using the Science Direct database to define a search equation with some keywords (“bacteria,” “chlorine,” “peroxide,” and “ultraviolet”) (Fig. 2). It is important to note that the search formula was chosen so that a manageable number of manuscripts were found for the evaluation of their titles and abstracts (between 90 and 400 for articles).

This search helped to narrow down the information and to obtain at least two manuscripts that contained two or more search terms or synonyms in the title and keywords. If the number of results was not sufficient to achieve the first goal, the search was repeated with a different combination of keywords. If no article contained all the keywords that were originally used for the search, a different of keywords was selected for another search (Table SM1). The search equation adapted to these conditions, based on the search for articles, would be the one used in the search for patents to perform the scientific-technological review under similar conditions.

The combination of keywords was searched in the four selected databases, from which all articles resulting from the search were collected. After identifying and eliminating duplicates and evaluating all titles, manuscripts not related to the topic of interest were eliminated. The result was a selected group of manuscripts defined as a portfolio. Their keywords were used to create the distribution map in the VOSViewer software, both for the unrefined searches and for the final portfolio.

### Bibliometric analysis

For the bibliometric analysis of the portfolio, the articles were sorted in a Google spreadsheet and ranked by the number of citations according to Google Scholar (https://scholar.google.com/) and the Scopus Preview author search tool (https://www.scopus.com/freelookup/form/author.uri?zone=TopNavBar&origin=NO%20ORIGIN%20DEFINED) selecting articles with more than 50 citations.

The filter recommended in the methodology for the selection of manuscripts (manuscripts with more than 85% of the total number of citations) was not applied (Costa et al. 2021; Linhares et al. 2019). This decision was made because the research topic is specific, recent, and relatively under-researched, resulting in a more homogeneous distribution of citations. The highest percentage of participation was around 11%, so the selection by percentage of participation was omitted and limited us to a minimum number of citations (50). A similar strategy was used for the selection of patents with a minimum of 10 citations. The highest percentage of participation in this case was 26%.

### Systemic analysis

For the systemic analysis, criteria such as system conditions, evaluated microorganisms, and system components, among others, were chosen to analyze the selected articles (Table 1). At the end of the analysis, possible questions were formulated to guide future research on the topic.

**Table 1:** Lenses and objectives are used in the systemic analysis applied in this review

No	Lenses	Goal
1	Conditions and components of the disinfection system	What are the technical components of the system? What parameters have been controlled or varied in the system (pH, reagent dose, radiation source, reactor volume, flow rate, batch or continuous mode, etc.)?
2	Physicochemical characteristics of the aqueous matrix	What are the physicochemical and microbiological characteristics of the disinfected water (real or synthetic, residual or potable, wastewater with or without pre-treatment, etc.)?
3	Inactivated microorganisms	Which species of bacteria are inactivated by the disinfection system (*E. coli*, *P. aeruginosa*, *A. fischeri*, *Campylobacter*, *Salmonella spp*., *Shigella dysenteriae*, or others)?
4	Associated ARG removed	Which ARGs have been eliminated by the disinfection system (name of gene, type, and to which antibiotic does it confer resistance)?
5	Efficiency of the disinfection system	How efficient was the disinfection of the water and under what system conditions?
6	Cost analysis of the disinfection system	Does the publication include an economic or energy cost analysis of the disinfection system?

## Results and discussion

### Development of the portfolio

#### Search for articles with the defined keywords and databases

The combinations of different keywords for the preliminary search allowed us to define the search equation that corresponded to the criteria initially established. To do this, it required finding a reasonable number of manuscripts and identifying at least two search terms or synonyms in the title and keywords. The first attempts, without using terms that would limit the search to water treatment, yielded many results (search Eqs. 1–3, Table SM1). However, subsequent attempts with combinations of keywords such as “disinfection,” “chlorine,” “gen,” “bacteria,” “ultraviolet,” “wastewater treatment,” and synonyms of these terms were not successful (search Eqs. 4–6, Table SM1). Therefore, other sets of search equation keywords were selected based on the main components of the system and their intended use (“bacteria” and “chlorine” or “chlorination” or “Cl_2_” and “H_2_O_2_” or “peroxide” and “ultraviolet” or “UV”) as search filters for the words present in the title. This search resulted in 98 articles in Science Direct (Eq. 8, Table SM1).

#### Distribution by type of document (articles and patents)

Several databases were used in the literature search, and all keywords were first entered into the search filter “anywhere in article” to ensure a uniform search method in all databases. The retrieval for articles and patents related to the use of disinfection systems based on UV, H_2_O_2_, and/or Cl_2_, both individually and in combination reflects the interest of the scientific and industry in the development of surface and environmental disinfection technologies. In terms of the number of articles found per database (Table 2), Science Direct had the most results with 3769 documents, followed by Wiley Online Library with 2150 documents and SpringerLink with 1714 documents, while ACS Journals Search had the fewest articles (928). This difference may be due to the specific focus of the individual database and the number of journals they cover. Science Direct, for example, covers a large number of journals and publications, which may be related to the ownership of Elsevier and its extensive network of scientific journals, as well as its focus on a variety of scientific disciplines. Conversely, the limited coverage of ACS Journals Search may be due to its focus on a specific subject area (chemistry and related sciences), limited access to some journals due to subscription contracts, and differences in journal publication policies (Chen 2010; Sichel-Crespo et al. 2022; Yu 2004).

**Table 2:** Number of publications per database by searching the final filter equation in title, abstract, and keywords ((“bacteria”) AND (“chlorine” OR “chlorination” OR “Cl”) AND (“H2O2” OR “peroxide”) AND (“ultraviolet” OR “UV”))

Article database					Total
ACS Journals Search	Science Direct (Elsevier)		SpringerLink	Wiley Online Library	
928	3769		1714	2150	8561
Patents database					Total
Patentscope (WIPO)		Espacenet		The Lens	
9247		1774		9923	20,944

In terms of patents, The Lens had the most results (9923 documents), followed by Patentscope-WIPO (9247 documents) and Espacenet (1774 documents). These results indicate that many companies and inventors are developing technologies related to UV, H_2_O_2_, and/or Cl_2_ disinfection. The higher number of patents found in The Lens database, especially compared to Espacenet, can be attributed to access to multiple patent sources, data mining and natural language processing technologies, and the inclusion of open access patents (Montecchi et al. 2013; van Rijn and Timmis 2023). However, it is important to point out that for a comprehensive overview of the state of the art and technology and to avoid bias in document selection, it is necessary to use multiple databases that complement each other in terms of coverage, research areas, access, and other factors.

In the research and application of UV, H_2_O_2_, and Cl_2_ disinfection systems for the elimination of ARBs and ARGs, a greater emphasis on technology than on scientific research was observed (as shown in Fig. 2 and Table 2), with technological publications representing approximately 71% of the total published documents (articles + patents). This remarkable difference can be attributed to three factors: (i) the nature of the research: patents are a form of intellectual property protection for new inventions and discoveries and are particularly important in industry and technology development. (ii) Commercial interests: companies and organizations may be more interested in protecting their inventions and discoveries through patents because it gives them exclusive rights to use and commercialize their inventions. (iii) Differences in review procedures: patents and scientific articles go through different review and approval processes. Patents are reviewed by patent examiners to determine whether they meet the requirements for patentability, while scientific articles undergo to peer review to assess their quality and scientific validity. The patent review process can be faster and less rigorous than the review process for scientific articles, which may contribute to a higher number of published patents (Azoulay et al. 2019; Bassecoulard and Zitt 2005; Meyer 2004).

#### Distribution by legal status in technological production (patents)

**Fig. 3:**
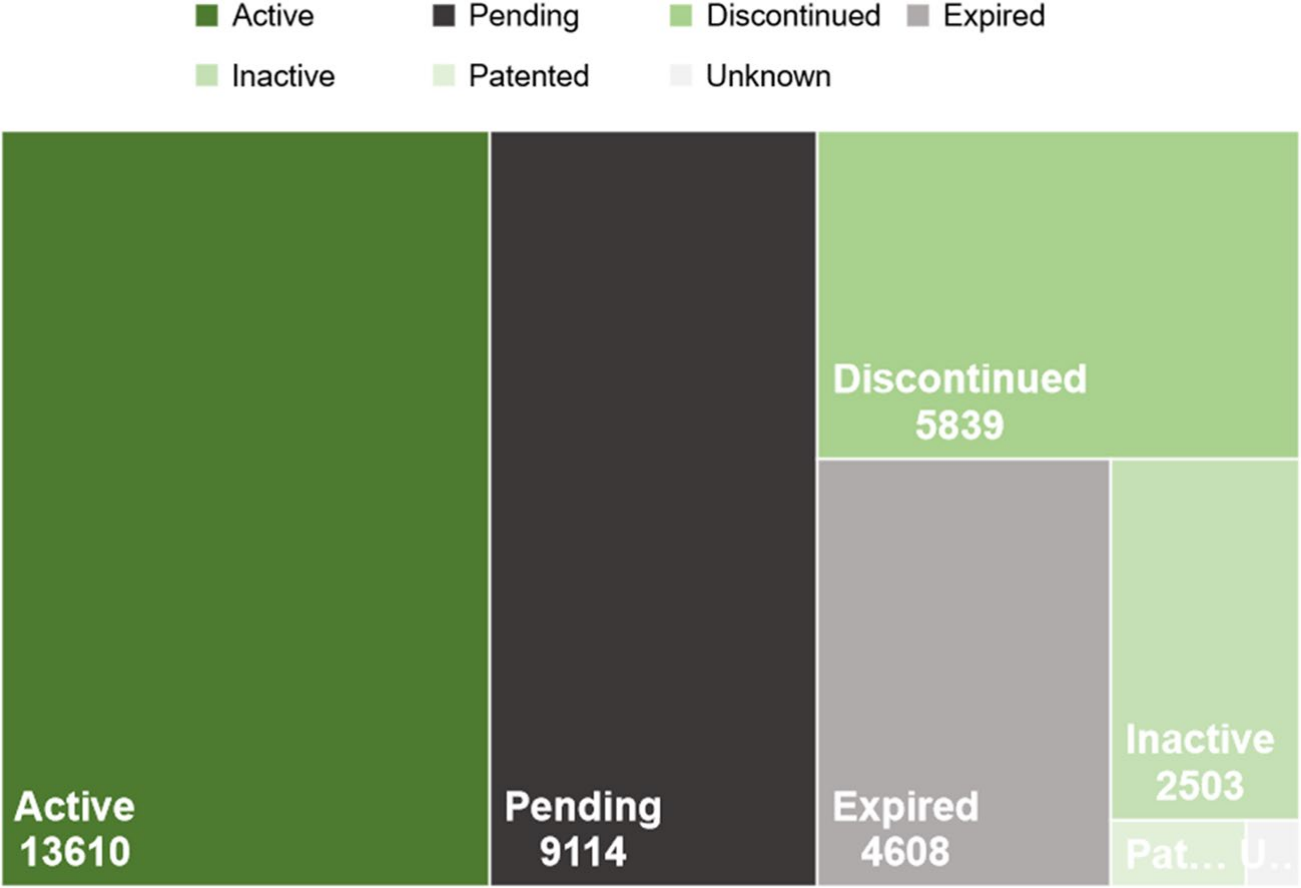
The number of patents by legal status. Data obtained after searching with the final filter equation in The Lens database (“bacteria”) AND (“chlorine” OR “Chlorination” OR “Cl”) AND (“H_2_O_2_” OR “peroxide”) AND (“Ultraviolet” or “UV”))

When considering technological production, it is significant to consider the legal status of patents, as this can have a significant impact on intellectual property protection and the commercial value of disinfection technologies and methods. To address this issue, an analysis of technological production was conducted based on the legal status of patents obtained from The Lens database (as shown in Fig. 3). Patents in active and pending status are those that are currently under evaluation and approval. Patents in active status (13,610 patents, representing 37.7% of the total documents in The Lens) have been approved and are currently in effect, while patents in pending status (9114 patents, representing 25.2% of the total documents) are still being evaluated by patent examiners. These patents can be particularly important to companies and organizations seeking to protect their disinfection technologies and methods, as they can have high commercial value and increase competition among companies and organizations.

Patents in discontinued status (5839 patents, representing 16.2% of the documents) and expired status (4608 patents, representing 12.8% of the documents) are no longer in force. Discontinued patents are those that have been abandoned by the patented or otherwise discontinued, while expired patents are those that have reached the end of their term. These patents may be of little commercial value to companies and organizations seeking to protect their disinfection technologies and methods. Patents in inactive status (2503 patents, with a 6.9% share of documents) are those that have not been renewed by the patent holder and have lost their validity. These patents may also have little commercial value. Patents in pending status (332 patents, representing 0.9% of documents) are those that have been approved and are currently in force but may be subject to litigation or other restrictions on their use and commercialization. Patents of unknown status (133 patents, representing 0.4% of documents) are those for which a clear classification or legal status has not been determined. Therefore, with a 37.7% share of documents in active legal status, it can be concluded that the industrial field related to the use of UV, H_2_O_2_, and/or Cl_2_ disinfection systems for the elimination of ARBs and/or ARGs is highly competitive and poses a challenge for the identification of innovative ideas or technologies with an inventive level and potential for industrial application. It should be noted, however, that this high number of patents changes after the application of participation or relevance filters in the bibliometric analysis phase. Nevertheless, the trends in the legal status of the documents finally selected remain consistent.

#### Geographical distribution

Regarding the total scientific and technological production between 2011 and 2022, distributed by country (as shown in Figure SM1), China leads in the production of articles related to the use of disinfection systems based on UV, H_2_O_2_, and Cl_2_ for the elimination of ARBs and ARGs, with a total of 946 articles. The Unites States (US) ranks second with 645 articles, followed by Spain with 516 articles. India (387), South Korea (301), Italy (215), Brazil (172), Canada (160), Japan (143), France (129), and the UK (73) are also listed with a significant number of published articles. These data suggest strong cross-country collaboration and growing global concern for the use of effective disinfection systems to combat antimicrobial resistance. It is also possible that the production of articles in a country is influenced by factors such as investment in research and development, available infrastructure, and the number of experts in the field, as is the case in more developed countries such as China and the US (Chen et al. 2006; Freeman 2005; Macías-Quiroga et al. 2021; Pivoto et al. 2018).

In terms of technological production in this area, the US leads by a wide margin with a total of 7062 patents published, followed by China with 1129 patents. Compared to the trend in the publication of articles, where China leads, there seems to be a significant discrepancy between the publication of patents and articles in this field. It is interesting to note that countries such as Spain (3) and France (0), which are among the top 10 countries for article publications, have very few published patents in this field. Conversely, countries such as Brazil (856) and Canada (1770) have a significant number of published patents but are not among the top countries for article publication. These data suggest that investment in research and development in this field may be more focused on obtaining patents than on publishing articles. Countries may also be prioritizing the development of practical solutions and technologies to address the problem of antimicrobial resistance rather than publication in scientific journals.

#### Themes of trend and development of current research

**Fig. 4:**
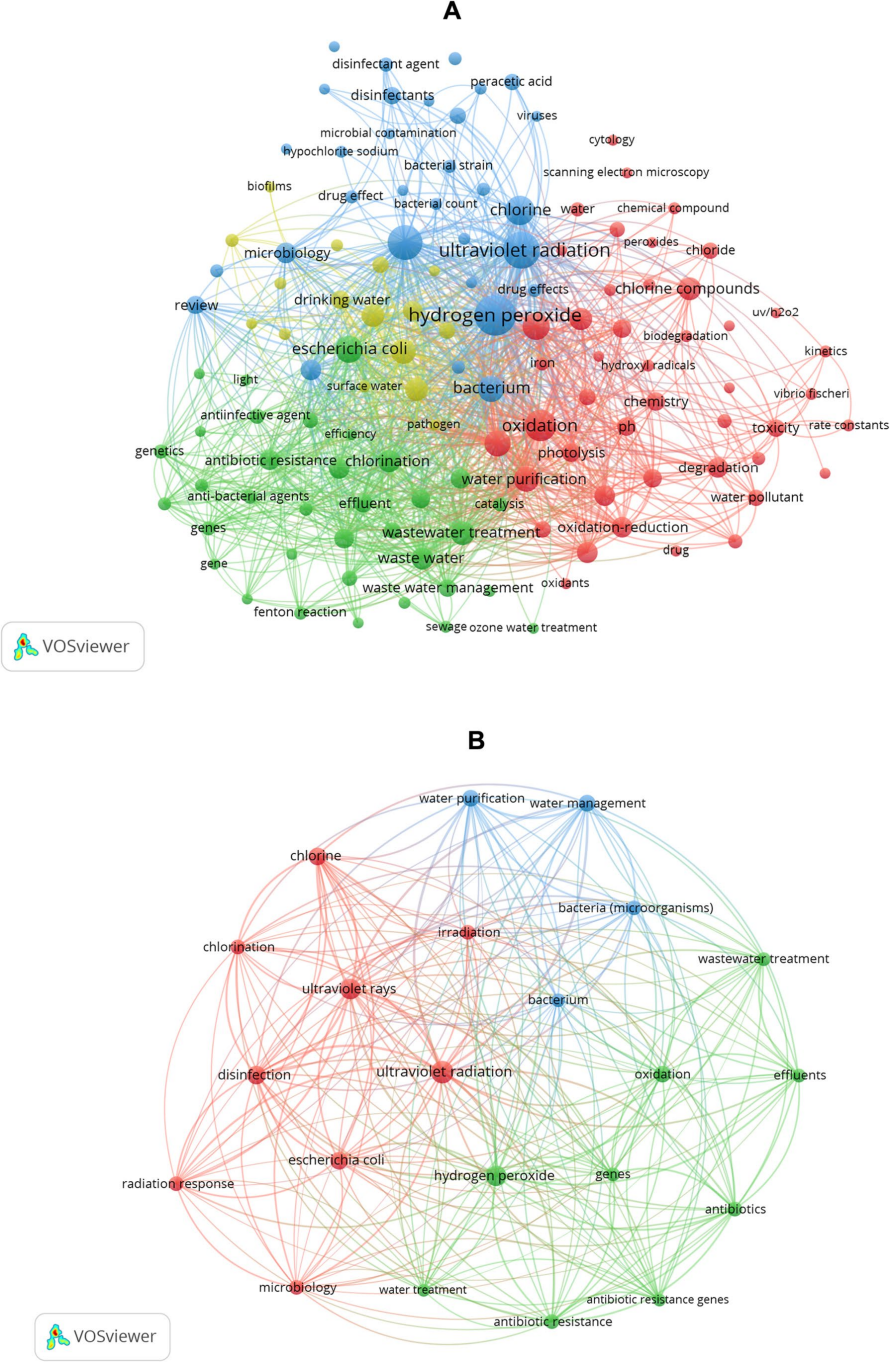
Map of keywords present in the title, abstract and keyword sections of all articles. **A** Retrieved from searches after the methodology compliance phase (94 articles). **B** Selected for the final portfolio (19 articles)

Furthermore, in the unrefined search (Fig. 4A), the three disinfection systems of interest—ultraviolet radiation, hydrogen peroxide, and chlorine—are highly cited exact keywords, indicating their broad relevance and importance in the search for documents (articles). They are a group within the keywords.

On the other hand, in the refined search (Fig. 4B), generic disinfection-related words and their synonyms or chemical compounds were observed. The presence of these very generic terms could give the impression of a lack of adherence to the research topic among the retrieved articles. However, in the refined portfolio, these generic terms are not as popular among the selected manuscripts, resulting in a clearer visualization of more specific terms. These include the most used model microorganism in disinfection studies (*Escherichia coli*), the appearance of the term ARGs with various synonyms, a general reference to disinfection, and the inclusion of oxidation. The UV, H_2_O_2_, and Cl_2_ systems, either individually or in combination, fall under the umbrella of advanced oxidation processes (AOPs). Although more specific terms can be observed, it is not specifically evident that ARBs or ARGs are more frequently cited, as there is a wide range of study models in these areas. Therefore, a bibliometric and systematic analysis (in-depth trend analysis) is needed to define research priorities and gaps.

### Bibliometric analysis of the portfolio

#### Selection for the final portfolio: keyword adherence, redundancy, title, and abstract selection

After applying the adherence filter, a portfolio of 30 manuscripts and 25 patents was obtained (as shown in Table SM2). The filter or selection was based on participation or relevance to the research, resulting in a total of 19 manuscripts and 18 patents selected for the bibliometric analysis stage. All these manuscripts had at least 50 citations up to the date of this work, with a total of 1124 citations among them. Of the selected manuscripts, ten were selected based on their high citation count, representing a total participation percentage between 11.6% and 4.0%. The remaining nine manuscripts were included in the portfolio as exceptions because they had relevant titles and abstracts and/or their authors were part of the author bank (as shown in Figure SM2, authors who are relevant in the publication of documents on the research topic). These manuscripts were published very recently and therefore had few citations (within the last 3 years, 2020–2022) (Costa et al. 2021; Eduardo Tasca et al. 2010), resulting in a total of 19 manuscripts for analysis (as shown in Table SM3).

The same approach has been taken for patents. As a technological production, the number of citations is typically lower, as it involves specific technical knowledge that may be useful in solving technical challenges in the cited inventions, making citation more difficult. Therefore, a minimum of 10 citations was set, resulting in a total of four patents selected for analysis, representing participation percentages ranging from 26.7 to 7.5%. In addition, another group of 14 patents with truly relevant titles, abstracts, and claims were added to the portfolio as exceptions. These patents were very recently published and had few citations within the last three years (2020–2022) (Costa et al. 2021; Eduardo Tasca et al. 2010).

#### General details in the scientific-technological production: portfolio for bibliometric analysis

The dataset of the 19 selected articles represents different bibliometric review articles on scientific production related to bacterial and ARG elimination in water treatment processes using AOPs for disinfection, specifically based on UV, H_2_O_2_, and/or Cl_2_ systems. Each record includes details such as title, reference, year of publication, number of citations, corresponding author, *h* index, and the journal in which the article was published (as shown in Table SM3). The most cited article in the dataset is “*Inactivation of Escherichia coli, Bacteriophage MS2, and Bacillus Spores under UV/H*_*2*_*O*_*2*_* and UV/Peroxydisulfate Advanced Disinfection Conditions*” (Sun et al. 2016), with 164 citations. This suggests that this article has been very influential in the scientific community and has been widely used as a reference in subsequent research.

Most of the articles in the dataset were published in high-impact journals in the field, such as *Environmental Science and Technology*, *Water Research*, and the *Journal of Hazardous Materials*. These three journals have the highest number of publications on the use of disinfection systems based on UV, H_2_O_2_, and/or Cl_2_, either individually or in combination. They are classified as the first quartile (Q1) according to SJR, indicating that these journals are in the top 25% of the impact factor distribution (Aparicio-Martinez et al. 2019; García et al. 2012; Vijayan and Renjith 2021). The journal-specific impact factors for JCR (2021) are 13.4 for *Water Research*, 11.75 for *Environmental Science and Technology*, and 14.224 for the *Journal of Hazardous Materials*. In addition, the three journals are among the most cited from 1980 to 2021. In 2021, *Water Research* has 36,528 citations, the *Journal of Hazardous Materials had* 52,787 citations, and *Environmental Science & Technology* has 48,242 citations. The journals that show the greatest interest in scientific production related to the use of UV, H_2_O_2_, and/or Cl_2_ disinfection systems are those that cover chemical engineering, environmental sciences, catalysis, and chemistry. The trend in the publication of articles in these high-impact journals has been previously observed in other works (Costa et al. 2021; Macías-Quiroga et al. 2021).

In addition, the fact that most of the corresponding authors of the most cited articles appear in multiple registries suggests that they are influential authors in the field. Notable authors with multiple publications on the topic include Luigi Rizzo, Huang Ching-Hua, Jingyun Fang, and Despo Fatta-Kassinos. Rizzo has authored three papers on the removal of antibiotic resistance genes by UV/H_2_O_2_ advanced oxidation and chlorination, and his *h* index is 46 (Di Cesare et al. 2020; Ferro et al. 2017; Fiorentino et al. 2015). Huang Ching-Hua is also the author of two articles on the removal of microorganisms and resistance genes using hydrogen peroxide and ultraviolet light and has a *h* index of 52 (Sun et al. 2016; Zhang et al. 2020). Jingyun Fang, co-author of two papers, has a *h* index of 39 (Guo et al. 2022; Wang et al. 2021). Finally, Despo Fatta-Kassinos, the first author of the article, has a *h* index of 66. Her study deals with a chemical, microbiological, and toxicological scheme to understand the efficiency of UV-C/H_2_O_2_ oxidation on micropollutants related to antibiotics in treated wastewater (Beretsou et al. 2020). It is worth noting that authors Huang Ching-Hua and Jingyun Fang had close collaborations with other authors in Asia, as evidenced by the articles present in the portfolio before the relevance filter was run (Figure SM2).

The 18 patents selected for the bibliometric review provide information on the technological production related to patentability in water and wastewater management. The table in SM3 provides details such as titles, numbers, years, number of citations, patent type, country, and inventors associated with each patent. The analysis revealed that most patents are directed to water and wastewater treatment, with the majority located in the US and China. In addition, the majority of patents are method patents, closely followed by apparatus and system patents. These findings suggest a significant focus on the development of water and wastewater treatment technologies. Of note, US10023484B2 describes the use of hydrogen peroxide and peracetic acid, along with peroxide-reducing agents, for the treatment of drilling fluids, fracturing fluids, flow back water, and washing backwater. This technology has been cited 39 times, indicating its widespread recognition in the industry. Patent US2013087504A1 describes a water treatment apparatus and methods of use and has been cited 13 times. This technology could be useful for purifying drinking water. Patent US2014353256A1 describes a multiple barrier system for water treatment and has been cited 11 times. This technology could be useful for treating municipal and industrial wastewater. Finally, patent US2014374103A1 describes a method for treating and recycling oil field wastewater and has been cited 11 times. This technology could be useful in reducing pollution from oil operations.

Overall, these data suggest that there is a significant amount of research and development in water and wastewater treatment technologies and that these technologies are becoming widely recognized in the industry. There are also a variety of patents related to water treatment, from equipment to multi-barrier systems to treatment processes.

#### Distribution of publications and co-publications in the main countries in scientific production

This study validated the effectiveness of the methodology used to perform a robust bibliometric search. The result was a list of highly relevant manuscripts and patents published by highly cited authors (as shown in Table SM3). To evaluate the final number of citations for each author, the Scopus tool was used due to the lack of specific profiles for some co-authors in Google Scholar. In addition, when considering authors with profiles available in both search tools, Google Scholar retrieved a greater number of manuscripts and citations, demonstrating its search efficiency.

Table 3 shows the number of joint publications between different countries in the field of ARB and ARG removal using disinfection systems based on UV, H_2_O_2_, and/or Cl_2_, either individually or in combination. China has the highest number of joint publications with other countries with a total of seven, followed by the US with three, and several countries with two and one joint publication. Overall, Asian countries, such as China and South Korea, have more collaborations with each other than European or American countries. However, it is important to note that the table only shows collaborations in the field of ARB and ARG removal using UV, H_2_O_2_, and Cl_2_-based disinfection systems, and there may be other research areas where European and American countries collaborate more frequently (Gazni et al. 2012; Macías-Quiroga et al. 2021; Sichel-Crespo et al. 2022; Sonnenwald 2007). In addition, the data show that some countries have more collaborations with certain partners than with others. For example, Spain appears to have more collaborations with Cyprus, Iran, and the Netherlands than with other countries listed in the table.

**Table 3:** Matrix of publications and co-editions for countries on UV, H_2_O_2_, and/or Cl_2_ removal systems in the final portfolio of articles

	China	US	South Korea	Italy	Spain	Cyprus	Iran	Canada	Germany	Netherlands
China	7	1	0	0	0	0	0	0	0	0
US	1	3	2	0	0	0	0	0	0	0
South Korea	0	2	1	0	0	0	0	0	0	0
Italy	0	0	0	2	1	0	0	0	0	0
Spain	0	0	0	1	0	0	1	0	0	0
Cyprus	0	0	0	0	0	1	0	1	1	1
Iran	0	0	0	0	1	0	1	0	0	0
Canada	0	0	0	0	0	1	0	0	1	1
Germany	0	0	0	0	0	1	0	1	0	1
Netherlands	0	0	0	0	0	1	0	1	1	0
Total	8	6	3	3	2	4	2	3	3	3

In analyzing the patents identified (Table SM3), a trend toward the invention of water treatment devices and methods using advanced oxidation technologies such as UV, H_2_O_2_, and/or Cl_2_ as disinfection systems, either in combination or individually, was observed. Five apparatus patents were found, indicating a focus on the development of water treatment devices. In addition, a significant number of system and process patents were observed, indicating a focus on improving the water treatment process. However, there were only three method and apparatus patents, indicating a potential gap in research to optimize existing water treatment systems and processes. In addition, only two system and method patents were found, highlighting an opportunity to explore new comprehensive water treatment solutions. Finally, it is important to note that most of the patents identified were filed in Asian countries, primarily China and South Korea, indicating a tendency in these regions to invest in water treatment technologies.

#### Distribution of scientific production—technologies with the different disinfection systems based on UV, H_2_O_2_, and Cl_2_ individually and in combinations

**Fig. 5:**
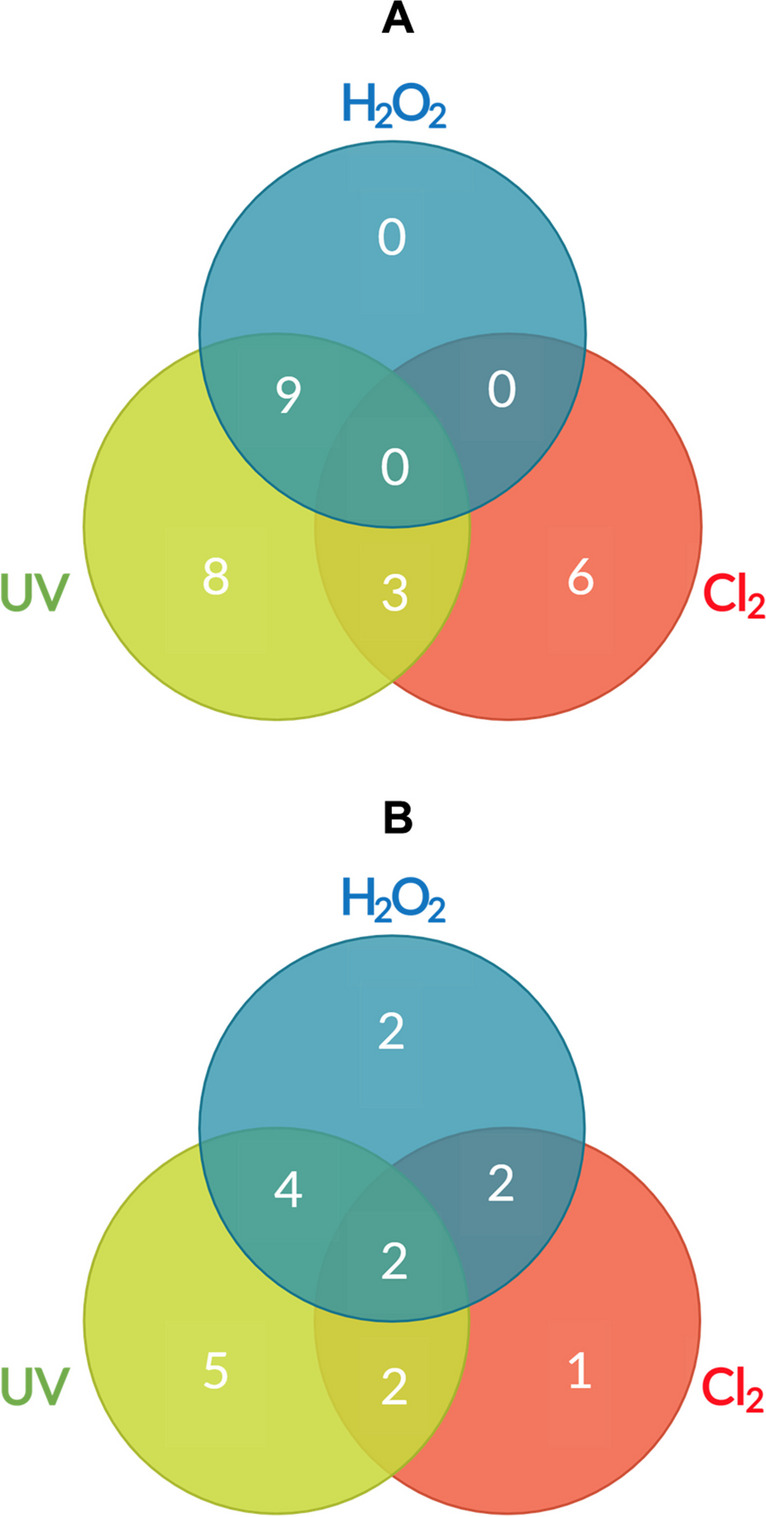
Occurrences and relationships between UV, H_2_O_2_, and/or Cl_2_ disinfection systems in the final portfolio. **A** Articles (19 publications). **B** Patents (18 publications)

Based on the results of the bibliometric analysis (Fig. 5A), the use of disinfection systems using combined UV and H_2_O_2_ (9 papers) appears to be the most popular approach in ARB and ARG removal research. It is interesting to note that although the use of H_2_O_2_ alone and the combination of Cl_2_ and H_2_O_2_ have not been the subject of published studies, it could be inferred that the use of Cl_2_ alone may not be as effective in removing ARBs and ARGs, as there are only six articles published on this system. It is important to note that the results of a bibliometric analysis may be limited by the availability and quality of the data, as well as the scope and precision of the search terms used. Furthermore, the number of published articles cannot be used to infer the actual effectiveness of disinfection systems, as there may be many factors that influence the effectiveness of these systems in different settings and situations. In general, the results indicate that there is a growing interest in the use of disinfection systems based on UV, H_2_O_2_, and Cl_2_ for the removal of ARBs and ARGs in both scientific and technological production. In the bibliometric analysis of patents, it is observed that the combined use of the three disinfection systems (UV/H_2_O_2_/Cl_2_ with 2 patents) is less common than their individual use (UV = 5, H_2_O_2_ = 2, and Cl_2_ = 1) or in the combination of two (UV/H_2_O_2_ = 4, UV/Cl_2_ = 2, and H_2_O_2_/Cl_2_ = 2), suggesting that researchers are exploring the effectiveness of combinations (Costa et al. 2021; Gandhi and Prakash 2023; Kokkinos et al. 2021; Umar 2022; Walker et al. 2015).

#### Documents selected for the systematic review stage

Of the 19 manuscripts and 18 patents initially selected, five manuscripts and five patents were selected for three main reasons: (i) to focus on the simultaneous study of ARBs and ARGs removal; (ii) to examine the application of different disinfection systems, including single treatments such as UV, H_2_O_2_, and/or Cl_2_, binary combinations such as UV/H_2_O_2_, UV/Cl_2_, and H_2_O_2_/Cl_2_, and tertiary combinations such as UV/H_2_O_2_/Cl_2_, and tertiary combinations such as UV/H_2_O_2_/Cl_2_; and (iii) ensuring that each document was selected from a different section of the citation list or percentage of participation to obtain a broad range of data without being biased toward the most highly cited documents. In the case of the selected patents, a fourth criterion was added: (iv) the selected documents had to be classified as different types of patents (apparatus, method, system, apparatus and method, or method and system) to obtain a diverse range of data without being biased towards a particular type of patent.

Consistent with most of the cited keywords (Table SM1 and Fig. 4B), the selected manuscripts employed different systems of interest, including UV, Cl_2_, UV/H_2_O_2_, and UV/Cl_2_ combinations, as well as other advanced oxidation processes commonly used in wastewater disinfection, such as O_3_, hydroxyl radical generation, UV/peroxydisulfate, and UV/H_2_O_2_ modification with a Cu-IDS complex.

In the dataset of five articles on ARBs and ARGs removal by disinfection systems (Table 4), the most cited article is “*Degradation and Deactivation of Bacterial Antibiotic Resistance Genes during Exposure to Free Chlorine, Monochloramine, Chlorine Dioxide, Ozone, Ultraviolet Light, and Hydroxyl Radical*” published in 2019 in the journal Environmental Science and Technology, with a total of 115 citations and a 10.23% share of the dataset (He et al. 2019). The most cited authors are Michael C. Dodd from the Department of Water and Environmental Sciences at the University of Alabama and Xiaochang Wang from Tongji University, each with 3 articles and a total of 3360 and 12,093 citations, respectively. In terms of *h* index and SJR, the most influential article is “*Reduction in horizontal transfer of conjugative plasmid by UV irradiation and low-level chlorination*” published in 2016 in the journal Water Research (Lin et al. 2016). Lead author Xin Yu’s *h* index is 327, while the journal’s SJR index is 2806.

**Table 4:** Summary of the bibliometric analysis: publications selected and statistical data

Articles							
Title	Year	Citations (% participation)	Most cited author	No. of author citations	Journal	Index *h*	Index SJR^a^
Degradation and Deactivation of Bacterial Antibiotic Resistance Genes during Exposure to Free Chlorine, Monochloramine, Chlorine Dioxide, Ozone, Ultraviolet Light, and Hydroxyl Radical	2019	115 (10.23%)	Michael C. Dodd	3360	Environmental Science and Technology	70	2.702
Reduction in horizontal transfer of conjugative plasmid by UV irradiation and low-level chlorination	2016	74 (6.58%)	Xin Yu	2249	Water Research	327	2.806
Mechanisms of ultraviolet disinfection and chlorination of *Escherichia coli*: Culturability, membrane permeability, metabolism, and genetic damage	2018	68 (4.81%)	Xiaochang Wang	12,093	Journal of Environmental Sciences	109	1.205
Combination of flow cytometry and molecular analysis to monitor the effect of UVC/H_2_O_2_ vs UVC/H_2_O_2_/Cu-IDS processes on pathogens and antibiotic resistant genes in secondary wastewater effluents	2020	25 (1.77%)	Luigi Rizzo	9016	Water Research	327	2.806
Free radicals removing extracellular polymeric substances to enhance the degradation of intracellular antibiotic resistance genes in multi-resistant *Pseudomonas putida* by UV/H_2_O_2_ and UV/peroxydisulfate disinfection processes	2022	9 (0.64%)	Guanyu Zheng	1195	Journal of Hazardous Materials	307	1.991
Patents							
Title	Year	Citations (% participation)	No		Type of patent	Country	
Water treatment device and methods of use	2015	13 (10.08%)	US2013087504A1		Apparatus	US	
Method and special equipment for removing antibiotic-resistant bacteria and resistant genes in sewage	2020	2 (1.55%)	CN111620493A		Method and apparatus	China	
Wastewater composite disinfection process for harmless treatment of livestock and poultry died of illness	2020	2 (1.55%)	CN111056701A		System	China	
Technology for producing a disinfectant for neutralizing viruses, bacteria and other microorganisms	2020	1 (0.78%)	WO2020019047A1		System	Brazil	
Water treating apparatus based on advanced oxidation	2021	0 (0.00%)	KR20210042540A		Apparatus	South Korea	

^a^SJR index: indicator of the average prestige of each journal per article in the period 2021 (most recent published period within the analysis) (Di Cesare et al. 2020; He et al. 2019; Lin et al. 2016; Meng et al. 2022; Xu et al. 2018)

Importantly, the majority of articles in this dataset were published in high-impact water and environmental science journals, indicating a growing concern within the scientific community about the risks of spreading ARBs and ARGs through water. In addition, research focuses on different types of disinfection systems, highlighting the need to develop effective and sustainable technologies to combat the spread of ARBs and ARGs in water (Lin et al. 2016; Morante-Carballo et al. 2022; Navarro et al. 2023; Niegowska et al. 2021; Sanseverino et al. 2022).

Regarding the selected technological production (Table 4), a similar number of patents was observed in the types of patents related to systems or apparatus. In addition, a higher number of citations to water treatment patents were found in the US compared to other countries such as China, Brazil, and South Korea. Specifically, the US has 13 citations related to one patent (US2013087504A1), while China has two citations across the two selected patents (CN111620493A, CN111056701A), Brazil has one citation (WO2020019047A1), and South Korea has no citations (KR20210042540A). In terms of specific technologies, it is noteworthy that a patent was filed in Brazil for the manufacture of a disinfectant to neutralize viruses, bacteria, and other microorganisms (WO2020019047A1), indicating a focus on developing innovative technologies to combat harmful microorganisms that can affect both humans and animals.

In addition, the patent relating to the composite disinfection process of wastewater for the safe treatment of livestock and poultry killed by the disease in China (CN111056701A) indicates a specific approach to addressing the problem of harmful microorganisms in the agricultural industry. It is important to note that the number of patents does not necessarily reflect the current level of research or development in each area, but it can provide insight into areas of interest to companies and institutions. Therefore, in the previous sections of this work, all the data obtained from the patents were analyzed to determine trends, and in the next sections (“Systematic analysis of the portfolio”), the gaps associated with the information provided in the patents for the most important technological production will be evaluated.

### Systematic analysis of the portfolio

#### Development of the first lens: conditions and components of the disinfection system

##### General information obtained in the articles

The complete systematic analysis carried out for each article is summarized and illustrated in Table 5. The analysis of the five selected articles shows that all of them describe the controlled or varied parameters in the disinfection systems. In addition, they provide details about the reactors, flow, mode (continuous or batch), reagent dosages (such as buffers and disinfectants), optimal pH for each disinfection system, optimal temperatures, irradiation times in UV disinfection, specific irradiation lengths, types of adjuvants, and concentrations of all reagents. The article “*Degradation and Deactivation of Bacterial Antibiotic Resistance Genes during Exposure to Free Chlorine, Monochloramine, Chlorine Dioxide, Ozone, Ultraviolet Light, and Hydroxyl Radical*” is the most detailed description of the parameters controlled in disinfection systems. It provides complete information on the different disinfectants evaluated and describes the parameters of each disinfection system. In addition, the supplementary material provides additional details on the disinfection systems (Table 5) (He et al. 2019).

**Table 5:** Summary of the systematic analysis of the manuscript: publications selected

Article: Degradation and Deactivation of Bacterial Antibiotic Resistance Genes during Exposure to Free Chlorine, Monochloramine, Chlorine Dioxide, Ozone, Ultraviolet Light, and Hydroxyl Radical (He et al. 2019)				
Lens	The lens is dealt?	If yes, how is this lens dealt with in the publication?	Main strengths	Main weaknesses
Conditions and components of the disinfection system	Yes	In the methodology section, they summarize the important parameters of the disinfection systems used. In addition, the supplementary material describes in detail the conditions and parameters of the systems, such as reactor, flow, mode (continuous or discontinuous), reagent doses (buffers and disinfectants), optimal pH for each elimination system, optimal temperatures of radiation in UV disinfection, specific radiation lengths (they use different types of UV), they specify the devices or equipment they implement to facilitate the operation of the system, etc For example, if they use UV irradiation to remove DNA, they use a 10 mM phosphate buffer (PB) autoclaved at pH 7 and contained in quartz tubes irradiated with a low-pressure mercury lamp (emission at 254 nm) in a carousel photoreactor	When studying different types of disinfectants, there is a wide range of possibilities to determine the most efficient in eliminating genetic material (DNA, specifically genes for resistance to intracellular and extracellular antibiotics (eARG and iARG)) The study makes it easy to compare disinfectants commonly used in water treatment, such as free available chlorine (FAC), NH_2_Cl, O_3_, ClO_2_, UV light (254 nm), and -OH from H_2_O_2_ as a precursor, and to use them in established drinking water ranges	The publication does not consider other alternatives to the disinfectants studied, focusing on lower reagent consumption, lower energy consumption, ease of disposal or use (e.g. UV LEDs)
Physicochemical characteristics of the aqueous matrix	Yes	In the methodology section, they describe the aqueous solution to be treated. In this study, they focus only on evaluating the efficiency of DNA removal (eARG and iARG), which is why they promote the growth and maintenance of the microorganism (*Bacillus subtilis*) in a buffer that avoids any cell damage due to stress. They do not introduce real or synthetic water	The use of a buffer to avoid stress to the microorganism makes it easier to determine the effect of the disinfectants on the microorganism and DNA without false positives	The effect of the physicochemical characteristics of the water (residual, irrigation, or synthetic) on the elimination of the microorganism (*Bacillus subtilis*) or DNA (eARG and iARG) under the specific conditions of the different disinfection systems is completely unknown. This may have a negative or positive effect on the elimination efficiency of the micro-organism and DNA
Inactivated microorganisms	Yes	Elimination of the species *Bacillus subtilis* (*B. subtilis*). In the introduction, they described why it was chosen as a model bacterium, highlighting characteristics associated with ease of use in the laboratory, high pathogenicity, and the ability to horizontally transfer ARG	The use of a model bacterium associated with diseases of the digestive system, such as acute diarrhoeal diseases (ADD), the main health problem caused by poor water quality and crops irrigated with this water, for which to evaluate the elimination of ARG and the same bacterium can give a first good approximation to the disinfection of water contaminated with this microorganism or bacilli with similar characteristics	Since it is a single species under fairly controlled conditions, it is completely unaware of the effect of the physicochemical characteristics of the water (residual, irrigation, drinking, or synthetic) on the elimination of the microorganism (*Bacillus subtilis*), as well as the effect of the interaction with bacteria of other species
Associated ARG removed	Yes	Deletion of the *B. subtilis 1A189 bltR*-*blt*-*bltD* gene with the *acfA* mutation (mutation associated with gene overexpression). Gene associated with the generation of resistance to multiple antibiotics	The results of ARG deletion are consistently confirmed in the study because it is evaluated in terms of ARG degradation (decrease in ARG copy number as measured by qPCR), ARG deactivation (elimination of ARG transforming activities), and inactivation of the microorganism as a donor ARB cell This involves the use of molecular biology techniques such as qPCR to analyze alternative sequences in the bacterial chromosome. This is an ARG elimination study with important conceptual and methodological foundations for future studies	qPCR results as false positives due to overestimation of the real inactivation levels, for which further studies are needed, and even more with other ARGs of different composition (content in bp), different characteristics and from other species
Disinfection system efficiency	Yes	The efficiency is described in terms of the reduction in the number of ARG copies, the elimination of ARG transformation activities and the inactivation of the microorganism (*B. subtilis* bacteria) They found that when FAC, O_3_, or UV were applied, eARG and iARG were degraded in percentages greater than 90%, but NH_2_Cl and ClO_2_ were inefficient for complete degradation	Using weighted linear regressions, among other techniques, they explained the differences in the efficiency of ARG removal by the behavior of the disinfectants with DNA (by the amount of bp, AT or GC content, doublets, triplets, etc.) They performed a normalization of the number of amplicon copies, the rates of DNA elimination and the elimination of the microorganism (*Bacillus subtilis*), which facilitates the comparison of the disinfection efficiency between the systems, regardless of the differences in the conditions and components of each disinfection system	The efficiencies in terms of decreasing number of copies, rates of elimination of ARG transformation activity and of the bacterium are shown, but the percentages of efficiency are not shown anywhere, nor are tables with specific data on the elimination of ARG and the model bacterium
Cost analysis of the disinfection system	No	-	-	-
Patent: Water treatment device and methods of use (US2013087504A1)				
Lens	The lens is dealt?	If yes, how is this lens dealt with in the publication?	Main strengths	Main weaknesses
Conditions and components of the disinfection system	Yes	The claims section specifies the conditions and technical components of the system. The technical components of the system described in the patent are a treatment chamber housing with an inlet and an outlet, an ultraviolet (UV) radiation source located inside the treatment chamber housing, and a treatment chamber inlet for introducing an oxygen-containing pressurized gas stream into the interior volume of the treatment chamber housing. The parameters mentioned in the patent are the oxygen-containing pressurized gas stream, the UV radiation source and the treated water stream formed after the process	The strength of the system described in the patent is that it uses UV radiation and oxygen to disinfect water. UV radiation is effective in destroying microorganisms and pressurized oxygen helps to accelerate the chemical reaction. In addition, the system is applicable to various types of water, including cooling tower water, swimming pool water, spa water, hot tub water, fracking water, water associated with oil and gas production, water produced with oil and/or gas, injection water, and water associated with primary, secondary and tertiary production of oil and/or gas	One of the weaknesses of the system described in the patent is that it does not specify the exposure time required for the UV radiation and pressurized oxygen to effectively disinfect the water. In addition, the patent does not specify the mode of operation of the system (batch or continuous), nor the parameters that need to be controlled or varied in the system to ensure adequate disinfection of the water, such as pH, reagent dose, reactor volume, and flow. They do not explore other alternatives to the disinfectants studied, focusing on lower reagent consumption, lower energy consumption, ease of disposal or use (e.g., UV LEDs)
Physicochemical characteristics of the aqueous matrix	No	-	-	-
Inactivated microorganisms	Yes	The detailed description section of the patent refers to heterotrophic bacteria in general, including species within the genera *Pseudomonas*, *Aeromonas*, *Alcaligenes*, *Acinetobacter*, *Klebsiella*, *Flavobacterium*, *Chromobacterium*, and others. *Legionella* growth reduction and *Listeria* eradication in feedwater are also mentioned	The strength of the patent is that the water treatment device is capable of reducing the population of heterotrophic bacteria, *Legionella* and *Listeria* in water, which are often associated with causing disease in both healthy and immunocompromised mammals. This may render the water unfit for its intended use, such as drinking water or discharge into a river. It is also mentioned that the water treatment device can reduce coliform levels in feedlot water by a factor of about 1000, indicating remarkable effectiveness in reducing bacteria	The weaknesses of the patent may be related to the limitation on the elimination of other types of bacteria or microorganisms that are not mentioned in the patent. In addition, it is not specified whether the device is effective against genetic material such as ARG present in water. It is also important to note that the removal of bacteria may not be sufficient to ensure the safety of the water, particularly with regard to the presence of toxins or chemicals in the water. Therefore, a comprehensive approach and combination of treatments is required to ensure water quality
Associated ARG removed	No	-	-	-
Disinfection system efficiency	Yes	The efficiency of water disinfection varies depending on the bacteria and the type of water being treated, but in general, there is a significant reduction in the concentration of bacteria. For swimming heterotrophic bacteria, the reduction is usually over 90% and in some cases up to 99.9%. In the case of *Legionella*, the reduction in free-floating concentration is usually over 60% and in some cases as high as 85%. In addition, they see significant reductions in bacterial colonies in feedwater, including coliforms, *Staphylococcus Aureus*, *Escherichia coli*, and *Listeria* As the system is protected to achieve these high efficiencies, the system conditions are the same as those described in Lens 1	The patent shows great strength in the efficiency of water disinfection, as a significant reduction in the concentration of bacteria is observed in different types of water. It also mentions different types of bacteria that can be killed, suggesting that the water treatment device is effective against a wide range of micro-organisms. It is also mentioned that the treated water meets industrially accepted recommendations for biofouling control	A potential weakness of the patent is that it describes the efficiency of water disinfection in general terms, without detailing the specific conditions of the water treatment system that could affect the effectiveness of the device in practical situations. In addition, no information is provided on the safety of the device in terms of potential generation of toxic by-products or degradation of water quality Another weakness is that the patent does not provide information on the costs associated with the use and maintenance of the water treatment device. More information would therefore be needed to determine the feasibility and potential application of this technology in practice. In addition, the efficiency is not presented in figures or tables, nor is there any data from the statistical analysis of the results, which would provide a clear and concise picture of the efficiency
Cost analysis of the disinfection system	No	-	-	-

The article “*Reduction in horizontal transfer of conjugative plasmid by UV irradiation and low-level chlorination*” focuses on UV radiation and chlorination to reduce horizontal transfer of conjugative plasmids. The study describes the parameters of the disinfection systems, such as the different frequencies of UV radiation and the doses of free chlorine used (Table SM4) (Lin et al. 2016). In the article “*Mechanisms of ultraviolet disinfection and chlorination of Escherichia coli: Culturability, membrane permeability, metabolism, and genetic damage*,” disinfection systems based on UV and chlorination are compared. The study provides details on the mercury lamp used for UV radiation, as well as the concentration of free chlorine in the chlorination system. The pH and the volume of the reactor were kept constant in all the experiments (Table SM5) (Xu et al. 2018).

The article “*Combination of flow cytometry and molecular analysis to monitor the effect of UVC/H*_*2*_*O*_*2*_* vs UVC/H*_*2*_*O*_*2*_*/Cu-IDS processes on pathogens and antibiotic resistant genes in secondary wastewater effluents*” compares two disinfection systems: UV-C/H_2_O_2_ and UV-C/H_2_O_2_/Cu-IDS. It provides details on the intensity of the UV lamp, the concentration of H_2_O_2_ and Cu-IDS, the volume of the reactor, and the exposure time. The pH remained constant in both disinfection systems (Table SM6) (Di Cesare et al. 2020). Finally, in the article “*Free radicals removing extracellular polymeric substances to enhance the degradation of intracellular antibiotic resistance genes in multi-resistant Pseudomonas Putida by UV/H*_*2*_*O*_*2*_* and UV/peroxydisulfate disinfection processes*,” three disinfection systems are compared: UV, UV/H_2_O_2_, and UV/peroxydisulfate (PDS). Details of the UV lamp used, the dose of H_2_O_2_ or PDS, the UV fluence, the volume, and the distance between the lamp and the reactor are described (Table SM7) (Meng et al. 2022). Strengths of the articles include a detailed description of the disinfection systems used, which allows replication of the studies by other researchers. In addition, several of the articles combine molecular and microbiological techniques to assess the impact of treatment on microorganisms and antibiotic resistance genes, providing a comprehensive assessment of the effectiveness of disinfection systems.

##### General information obtained in the patents

On the contrary, the articles, with the patents (Table 5, Table SM8–SM11), show a greater variety in the disinfection systems based on UV, H_2_O_2_, and/or Cl_2_, with greater combinations, implementation of electrochemical cells, and couplings with other systems. Specifically, UV in the patent “*Water treatment device and methods of use*” (US2013087504A1, Table 5), UV/H_2_O_2_ electrochemically assisted in the patent “*Method and special equipment for removing antibiotic-resistant bacteria and resistant genes in sewage*” (CN111620493A, Table SM8), the UV/Cl_2_ system in the patent “*Wastewater composite disinfection process for harmless treatment of livestock and poultry died of illness*” (CN111056701A, Table SM9), electrochemically assisted H_2_O_2_/Cl_2_ modified with myeloperoxidase (MPO) enzyme as an adjuvant in the “*Technology for producing a disinfectant for patent neutralizing viruses, bacteria and other microorganisms*” (WO2020019047A1, Table SM10), and UV/H_2_O_2_/O_3_ in the patent “*Water treating apparatus based on advanced oxidation*” (KR20210042540A, Table SM11). These patents share the common goal of achieving greater efficiency and speed in the disinfection process while using more advanced and safer technologies. In terms of the parameters they control, most of the patents relate to the dose of the reagent used, the radiation source (whether UV or visible light), the speed of the water flow, and the exposure time.

It should be noted that these individual or combined UV, H_2_O_2_, and/or Cl_2_ disinfection systems presented in the patents include in some cases combinations with other systems such as ultrasound (CN111620493A), with primary treatments (sand filtration, flocculation, and sedimentation) and biological treatments (CN111056701A), with a cartridge filter (WO2020019047A1), and ozone (KR20210042540A). These combinations between different treatments can improve the efficiency of removing contaminants in the water by taking advantage of the strengths of each process and minimizing its limitations. In addition, the combination of different treatment processes can also help reduce costs and improve the sustainability of water treatment (An et al. 2008; Cerveira et al. 2022; Czarnitzki et al. 2014; Pavitt 1985).

Among the strengths of these patents is their potential to improve the effectiveness of water disinfection processes, which can help prevent disease and ensure the safety of drinking water. In addition, several of these patents represent a more sustainable and environmentally friendly approach, using technologies that require less energy and do not produce toxic by-products (Li et al. 2021; Zinn et al. 2018). However, some weaknesses have been identified in these patents. In some cases, the information provided is limited and details of the technical components of the disinfection system used are not provided. In addition, the practical application of these technologies may have certain limitations, such as the need for more physical space to install the equipment or the need for continuous monitoring of the process. A possible explanation for the lower level of detailed information in patents compared to articles may be that patents focus on protecting the intellectual and commercial property of the technology, while articles aim to share knowledge and scientific information more broadly. Therefore, less specific technical information may be presented in patents to avoid the risk of unauthorized copying and reproduction of the patented technology (Basberg 1987; Czarnitzki et al. 2014; Ernst 2003; Meyer 2004; Pavitt 1985).

##### Potential use of electrochemical cells

Similarly, in the five articles selected for systematic analysis and those previously selected in the bibliometric analysis portfolio (Table 5, SM3–SM7), H_2_O_2_ and Cl_2_ are used by adding a certain concentration for the elimination of ARBs and ARGs. They achieve elimination efficiencies between 70 and 100% in 10 to 30 min of treatment, with different efficiencies depending on the disinfectant and doses used. This practice of constantly adding H_2_O_2_ or Cl_2_ reagents has several advantages. Firstly, both are dangerous chemical compounds that require safety measures and special security for handling and storage. Cl_2_ is a toxic and corrosive gas that can cause eye and skin irritation, while H_2_O_2_ and Cl_2_ (in NaClO or similar precursors) are corrosive and oxidizing liquids that can cause burns. Secondly, the cost of H_2_O_2_ and Cl_2_ reagents can vary depending on where they are purchased, and the quantity required, and can be high. Third, disinfecting water with H_2_O_2_ and Cl_2_ requires the addition of these chemicals to the water at specific times, which may not be optimal for efficient and consistent disinfection. Fourth, the use of Cl_2_ can produce by-products such as trihalomethanes and haloacetic acids, which are carcinogenic and have a negative impact on drinking water quality. In comparison, the use of an electrochemical cell that produces Cl_2_ and H_2_O_2_ in the water increases disinfection efficiency by providing constant production of the reagents in the water and eliminating the need to store and handle hazardous chemicals. In addition, the electrochemical cell reduces the formation of unwanted by-products, resulting in improved water quality in the treated water (Kerwick et al. 2005; Kraft 2008; Sales Monteiro et al. 2021).

The alternative to implementing chemical disinfection methods is the use of an electrochemical cell, which has been widely studied. However, there are still technical challenges in designing an electrochemical cell that can adapt to the specific needs of actual water treatment. Among the five patents evaluated in the systematic analysis phase, two of them implemented electrochemical disinfection systems (CN111620493A—Table SM8 and WO2020019047A1—Table SM10), although none of the analyzed articles implemented an electrochemical cell (Table 5, Table SM4–SM7). The design of an appropriate electrochemical cell involves consideration of factors such as cell geometry, size, number of electrodes, electrode arrangement, and distance between them, among others. In addition, the selection of appropriate materials for the electrodes is critical to the scalability of an electrochemical cell, as they must be corrosion resistant and able to withstand the extreme conditions of the large-scale electrochemical process. One of the biggest challenges in electrochemical systems is energy management. In scientific and technical production, reagent addition is still preferred over electrochemical disinfection because of factors such as the amount of energy required to operate the cell, energy efficiency, and safety in energy management. It is necessary to implement a monitoring and control system to measure the key parameters of the process and ensure that the quality of the treated water meets the required standards. In general, scaling up an electrochemical cell for real water treatment requires careful planning and consideration of several technical factors to ensure an efficient and safe process. This is an area of research with high potential for improving conventional disinfection systems using UV, H_2_O_2_, and/or Cl_2_ (Hand and Cusick 2021; Kraft 2008; Mosquera-Romero et al. 2023).

##### LEDs as an alternative with high potential in water disinfection processes

In the specific case of each article, there is a potential weakness in the studies as they focus on the limitations of commonly used disinfectants in water treatment and do not explore other alternatives that may have lower reagent consumption, energy consumption, or ease of use, such as UV-LEDs. An alternative to these disinfectants is the use of UV-LEDs, which are currently widely used in the research and application of AOPs in water treatment due to several advantages. First, they are more energy efficient than lamps used in AOPs, such as low-pressure mercury lamps. Second, they can emit light in an adjustable spectrum, allowing greater flexibility in the application of AOPs to target different contaminants in water, such as ARBs and ARGs. Third, they have a longer useful life than conventional material lamps, reducing maintenance and replacement costs. Fourth, they are safer than conventional lamps because they do not contain mercury or other hazardous substances. As a result, UV-LEDs are a more sustainable and efficient alternative in terms of energy and cost for the application of AOPs in water treatment. In addition, because UV-LEDs still emit in the UV range, they can directly damage bacterial DNA and prevent the spread of ARBs and ARGs, resulting in more effective water disinfection (Autin et al. 2013; Kebbi et al. 2020; Matafonova and Batoev 2018; Nguyen et al. 2019; Vilhunen and Sillanpää 2010).

##### The low scientific-technological interest on the combined systems H_2_O_2_/Cl_2_ and UV/H_2_O_2_/Cl_2_

Regarding the implementation of a disinfection system based on the combination of H_2_O_2_ and Cl_2_, none of the five articles selected for the systematic analysis (Table 5, Table SM4-SM7) or the articles in the final portfolio for the bibliometric analysis (Table SM3) implement or record the combined H_2_O_2_/Cl_2_ system. There may not be many registered articles using this system because it is a relatively new technology that is not yet widely used in water treatment. According to the technology production data (Table SM3), the use of the H_2_O_2_/Cl_2_ system has only been established since 2017.

Several authors have reported that the H_2_O_2_/Cl_2_ system has lower efficiencies compared to other disinfection systems (UV/H_2_O_2_ or UV/Cl_2_) due to parasitic reactions between H_2_O_2_ and Cl_2_. When the concentration of H_2_O_2_ is high, an exothermic reaction with Cl_2_ can occur, producing hydrochloric acid (HCl) and oxygen (O_2_). This reaction can generate heat and can be dangerous if not properly controlled. In addition, once the disinfectants are consumed in this parasitic reaction, there are no oxidants in the system to allow disinfection (elimination of ARB and/or ARG), which reduces the efficiency of the system (Chen et al. 2023; Kribeche et al. 2022; Mora et al. 2022; Salmerón et al. 2021). However, it is necessary to observe three reasons that may increase the interest in the study and use of the combined H_2_O_2_/Cl_2_ system, which may be ignored by some authors: (i) to minimize parasitic reactions between H_2_O_2_ and Cl_2_ in a combined disinfection system, the doses of each compound must be carefully controlled and the concentration of oxidants and reaction by-products in the treated water must be regularly monitored (Fu et al. 2023; Kribeche et al. 2022; Wang et al. 2019a, b, c). (ii) Catalysts or co-oxidants may be added to improve the efficiency of the oxidation process and reduce the formation of toxic reaction by-products (Abo Atia et al. 2021; Martínez-Pachón et al. 2021; Meena et al. 2021). (iii) When H_2_O_2_ and Cl_2_ are in solution, they can react with each other through an oxidation–reduction reaction, where Cl_2_ is reduced to chloride (Cl^−^) and H_2_O_2_ is oxidized to water (H_2_O) through a chemical reaction that produces hydroxyl radicals (HO∙). Hydroxyl radicals are highly reactive and can react with organic contaminants in the water, increasing the efficiency of oxidation (Chen et al. 2023; Feng et al. 2019; Kribeche et al. 2022; Mora et al. 2022; Salmerón et al. 2021; Yin et al. 2021). Similarly, it can happen that the hydroxyl radicals (HO∙) produced by the reaction between H_2_O_2_ and Cl_2_ may react with the molecular oxygen (O_2_) present in the water. This reaction produces singlet oxygen (O_2_∙^−^) and can increase the effectiveness of the disinfection and oxidation process (Al-Nu’airat et al. 2021; Chen et al. 2023; Kribeche et al. 2022; Krystynik 2022; Lu et al. 2022; Mora et al. 2022; Salmerón et al. 2021). On the other hand, at high Cl_2_ concentrations, hydroxyl radicals can react with Cl_2_ to produce hypochlorite (OCl^−^) and hypochlorous acid (HClO), active chlorine species (ACS) that have a lower reduction potential than Cl_2_, but still contribute to the treatment or disinfection of water (Angyal et al. 2023; Delgado-Vargas et al. 2022; Martínez-Pachón et al. 2021; Szczuka et al. 2022).

In contrast to the articles, the final portfolio of the bibliometric analysis included two patents implementing a disinfection system based on H_2_O_2_/Cl_2_ (US2014374103A1 and WO2020019047A1, Table SM3). The latter patent was included in the systematic analysis (Table SM10). The lower technological output for the H_2_O_2_/Cl_2_ system compared to other systems (Fig. 5) may be because patents focus on other disinfection technologies instead of the H_2_O_2_/Cl_2_-based disinfection system. Alternatively, it is possible that the patents have limitations on the practical implementation of the H_2_O_2_/Cl_2_-based disinfection system, such as technical or economic problems that prevent its large-scale implementation. This may reduce inventors’ interest in the H_2_O_2_/Cl_2_ system, especially if they do not see potential industrial applications or a relevant inventive step (Lobo and Strumsky 2019; Mao et al. 2021, 2022; Stryzhak et al. 2020).

Similarly, there is little or no scientific-technological production on the disinfection system that combines three disinfectants (UV/H_2_O_2_/Cl_2_). Figure 5B shows that only two out of 18 patents implemented this system. Specifically, patent CN104528957A (Table SM3) implements an electrochemical system with a cathode for generating H_2_O_2_ and a titanium oxide anode for generating ACS, in addition to a UV light lamp located between the electrodes. However, they did not provide details of the conditions or components of the system. Patent US2017044035A1 (Table SM3) includes the implementation of a variety of oxidants such as H_2_O_2_, potassium permanganate, chlorine, sodium hypochlorite, calcium hypochlorite, sodium percarbonate, sodium perborate, ozone, UV, or oxygen. It should be noted that these combinations, as mentioned above, are done to increase the efficiency in disinfecting water.

However, the lack of documents implementing a UV/H_2_O_2_/Cl_2_ disinfection system, especially in patents, suggests that this system is relatively new and has not been researched enough to generate patents. The two patents found were registered in 2017. It may also be that production costs are too high to justify large-scale implementation, especially for the electrochemical-assisted system.

The low scientific and technological production in the use of the UV/H_2_O_2_/Cl_2_ system prevents the advantages of the system from being explored, such as that this system can be more effective than separate disinfection systems in eliminating microorganisms, because each system component works synergistically to maximize the effectiveness of the disinfection process, generating a wide variety of oxidants (H_2_O_2_, Cl_2_, HClO, ClO, HO∙, O^−^∙, O_2_∙^−^, Cl∙, among others) (Belghit et al. 2020; Berruti et al. 2022; Djaballah et al. 2023). The combination of UV, H_2_O_2_, and/or Cl_2_ also helps to reduce the amount of by-products and residues produced during the disinfection process. This is because each component complements the others to reduce the undesirable side effects of the disinfection process (Li et al. 2022; Pai and Wang 2022; Tian et al. 2020; Wang et al. 2019a, b, c; Yang et al. 2023). Although implementing a combined UV/H_2_O_2_/Cl_2_ disinfection system can be expensive, it can be more economical in the long run than using separate disinfection systems because the combined system is more effective, reducing the need to repeat the disinfection process several times (Gandhi and Prakash 2023; Wang et al. 2019a, b, c). Similarly, the combined UV/H_2_O_2_/Cl_2_ system is more versatile than separate disinfection systems because each component of the system can be independently adjusted to meet different disinfection needs. With these advantages, the potential for the study and use of the UV/H_2_O_2_/Cl_2_ system is high (Kebbi et al. 2020; Lang et al. 2022; Paździor et al. 2019).

#### Development of the second lens: physicochemical characteristics of the aqueous matrix

In three of the five articles selected in this section (Table 5), they do not implement synthetic or real water, so in “*Degradation and Deactivation of Bacterial Antibiotic Resistance Genes during Exposure to Free Chlorine, Monochloramine, Chlorine Dioxide, Ozone, Ultraviolet Light, and Hydroxyl Radical*” (Table 5) as it focuses on evaluating the efficiency of DNA removal (eARG and iARG) through different disinfection processes, for which they did not implement a real or synthetic water sample, and they were limited to the use of a buffer to avoid stress on the bacteria that could generate erroneous results. Similarly, in “*Reduction in horizontal transfer of conjugative plasmid by UV irradiation and low-level chlorination*,” they implement sterile saline water and LB culture medium (Table SM4); in “*Mechanisms of ultraviolet disinfection and chlorination of Escherichia coli: Culturability, membrane permeability, metabolism, and genetic damage*,” they chose to use PBS buffer at pH 7.4 (Table SM5). In these cases, the only physicochemical characteristics detailed in the documents are the pH and the concentration of the buffers.

In general, in these articles where they do not implement any relevant water sample, they only detail the pH because it is an important factor in UV, H_2_O_2_, and/or Cl_2_ disinfection systems individually and in combination, as it can significantly affect the disinfection efficiency (Gao et al. 2020; Lee et al. 2022; Wang et al. 2019a, b, c). For example, when disinfecting with H_2_O_2_ and Cl_2_, pH is also an important factor. A pH that is too low can reduce the effectiveness of the disinfection because it can inhibit the formation of hypochlorous acid (HOCl), which is the most effective species for disinfection due to its reduction potential, as it has the highest (*E*° = 1.49) compared to the other ACS that may be present depending on the pH (pH < 3, Cl_2_ predominates with *E*° = 1.36 V, and at pH > 7, hypochlorite ions (ClO^−^) predominate with *E*° = 0.89 V). Therefore, for effective disinfection with H_2_O_2_ and Cl_2_, a pH between 3 and 7 is required to ensure optimal HOCl formation (Delgado-Vargas et al. 2022; Jin et al. 2011; Wang et al. 2019a, b, c; Wang et al. 2019a, b, c; Yin et al. 2021; Yoon et al. 2017).

On the contrary, in the other two articles selected in the systematic analysis stage, they detail the physicochemical characteristics of real wastewater samples, in the case of the article “*Combination of flow cytometry and molecular analysis to monitor the effect of UVC/H*_*2*_*O*_*2*_* vs UVC/H*_*2*_*O*_*2*_*/Cu-IDS processes on pathogens and antibiotic resistant genes in secondary wastewater effluents*” using actual samples of wastewater treated by primary and secondary systems at a WWTP from Verbania, Italy (Table SM6). The detailed description of the physical, chemical, and microbiological characteristics of the samples is a strength of the study, since they extend the characterization to parameters such as pH, COD, BOD_5_, SST, N-NO_2_, N-NO_3_, N-NH_4_, TP, and TN, and at the microbiological level, they detail 14 different genera (*Acinetobacter*, *Aeromonas*, *Bacillus*, *Bacteroides*, *Citrobacter*, *Enterobacter*, *Enterococcus*, *Escherichia-Shigella*, *Legionella*, *Morganella*, *Pantoea*, *Prevotella*, *Proteus*, *Pseudomonas*, *Serratia*, *Streptococcus*, and *Treponema*). Similarly, it happens in the article “*Free radicals removing extracellular polymeric substances to enhance the degradation of intracellular antibiotic resistance genes in multi-strong antibiotic Pseudomonas Putida by UV/H*_*2*_*O*_*2*_* and UV/peroxydisulfate disinfectant processes*” where they make an initial evaluation in sterile water, to be able to later go on to real wastewater to evaluate the effect of the matrix on the elimination of microorganisms and ARG allows the evaluation of the efficiency of the system in a relevant field, although with less detail of the physicochemical characteristics (pH, TOC, DO, COD, and conductivity) in comparison with the variety and quantity of the article mentioned above (Article 4: Table SM7).

It should be noted that the wide range of physicochemical characteristics detailed in the article “*Combination of flow cytometry and molecular analysis to monitor the effect of UVC/H*_*2*_*O*_*2*_* vs UVC/H*_*2*_*O*_*2*_*/Cu-IDS processes on pathogens and antibiotic resistant genes in secondary wastewater effluents*” is one of its greatest strengths (Table SM6). However, the number of physicochemical characteristics to be specified in a document can be limited to only the most important or those that limit the functioning of disinfection systems. As mentioned above, pH is a critical factor in the effectiveness of disinfection. It is significant to ensure that the pH is optimal for the disinfection system being used. In general, a pH between 6 and 8 is suitable for most disinfection systems, in addition to the fact that these pHs allow the treated water to be discharged after disinfection without the need for additional post-treatment to neutralize the water (Delgado-Vargas et al. 2022; Tamersit and Bouhidel 2020; Wang et al. 2019a, b, c; Yin et al. 2021; Yoon et al. 2017). Another characteristic is water turbidity, which can reduce the effectiveness of UV disinfection because particles can block UV rays and reduce the amount of light that reaches microorganisms. It is important to ensure that the turbidity of the water is low enough to ensure the effectiveness of UV disinfection (Kebbi et al. 2020; Li et al. 2019; Nguyen et al. 2019; Pichel et al. 2019).

A parameter that stands out in scientific production is the concentration of organic matter, measured as COD and BOD_5_ (Table SM6) or COD and TOC (Table SM7). The presence of organic matter in water can reduce the effectiveness of disinfection and increase the formation of toxic disinfection by-products. This can occur due to several factors. For example, (i) organic matter can absorb UV radiation, limiting the amount of radiation that reaches microorganisms in the water and reducing the effectiveness of disinfection. In addition, certain types of organic matter may absorb more UV radiation than others, resulting in a greater reduction in disinfection effectiveness (Płonka and Pieczykolan 2020; Shi et al. 2021). (ii) UV disinfection produces hydroxyl radicals (OH∙), which are very effective in eliminating microorganisms present in water. However, organic matter present in the water can react with these radicals and consume them, reducing their availability to disinfect the water. It also happens with H_2_O_2_ and Cl_2_ implemented as main disinfectants; they can be consumed to eliminate organic matter, instead of eliminating microorganisms, and in focus of this study, ARBs and ARGs (Guo et al. 2022; Martínez-Pachón et al. 2021; Meng et al. 2022; Núñez-Núñez et al. 2018). (iii) Some types of organic matter have the ability to form photoprotective compounds, which serve to safeguard microorganisms from the harmful effects of UV radiation, thereby reducing its effectiveness in water disinfection. Therefore, it is important to consider the concentration of organic matter in real water and make appropriate modifications to the disinfection conditions (Di Cesare et al. 2020; Giannakis et al. 2016; Lado Ribeiro et al. 2019; Rubio et al. 2013).

It is very important to record the physicochemical characteristics of the residual or real water used in disinfection studies with UV, H_2_O_2_, and/or Cl_2_ systems, either individually or in combination. This increases the reliability of the results and allows other researchers to reproduce the experiments and verify the results. It also helps to identify factors that may affect the effectiveness of disinfection and to adjust disinfection conditions to optimize results. On the other hand, keeping a record of the systems and types of water that have been efficiently disinfected allows the design of disposal systems adapted to the specific characteristics of other waters with similar characteristics. In this way, it is possible to design optimal disinfection systems for different types of water and ensure optimal disinfection effectiveness. It would also allow researchers to compare different disinfection systems and determine which is most effective for a given water body (Paschke 2003; Pereira et al. 2023).

Only the types of water treated are mentioned, and there is no record of the physicochemical characteristics of the water in technological production (four of the five selected patents) (Table 5, Table SM8–SM11). For example, patent US2013087504A1 (Table 5) mentions that various types of water can be treated, such as water used in cooling towers, chillers, recirculation systems, and wastewater treatment systems. Similarly, patent CN111620493A (Table SM8) focuses on wastewater from antibiotic production facilities, hospitals, municipalities, and aquaculture, while patents WO2020019047A1 (Table SM10) and KR20210042540A (Table SM11) focus on general water and wastewater treatment. Only in patent CN111056701A (Table SM9) do they specify the type of water to be treated, which is wastewater from the project for the safe disposal of sick and dead livestock and poultry. In this case, they describe some physicochemical and microbiological characteristics of the water, such as the range of fecal coliforms, COD, ammoniacal nitrogen, turbidity, and pH.

There is a big difference in the information registered on the physicochemical characteristics between the articles and the patents; this can happen because the patents are used to protect the intellectual property of a technology or process, which may include confidential information on the physicochemical characteristics of the treated water (Bassecoulard and Zitt 2005; Blackman 1995; Cohen et al. 2000; Meyer 2004; Willis 2021). In other cases, the inventors or patent holders may feel that the details about the treated water are not relevant to the description of the technology or process itself (Kieff 2003; Klemperer 1990). Similarly, the physicochemical properties of water can be very complex and vary significantly between different water sources, which could make it difficult to present detailed and accurate information about the treated water in a patent, as well as potentially limit the industrial applicability of the patent to a certain type of water or with certain characteristics, which is not beneficial to inventors (Ernst 2003; McGee 1966; Schmidt 2013).

Therefore, it is important to keep in mind that patents are a tool to protect intellectual property and describe specific technologies or processes. While it is desirable that patents include detailed information on the physicochemical characteristics of the treated water, it may not always be feasible to include such information due to resource limitations or the scope of the patent. Therefore, it is significant to evaluate each patent individually and consider other studies and scientific publications, such as peer-reviewed journal articles, to obtain more detailed information on the physicochemical characteristics of water treated with disinfection systems such as UV, H_2_O_2_, and/or Cl_2_, individually or in combination.

### Development of the third lens: inactivated microorganisms

Regarding the third objective, in the five articles analyzed, it is observed that they specify the type of microorganisms they inactivate. In all five cases, bacteria related to public health problems were inactivated, such as *E. coli* and *P. putida*, and characteristics such as resistance to multiple drugs and the ability to transmit ARGs. In addition, the importance of these bacteria in the spread of ARGs to other human pathogens is mentioned.

Specifically, the articles “*Degradation and Deactivation of Bacterial Antibiotic Resistance Genes during Exposure to Free Chlorine, Monochloramine, Chlorine Dioxide, Ozone, Ultraviolet Light, and Hydroxyl Radical*” (He et al. 2019), “*Reduction in horizontal transfer of conjugative plasmid by UV irradiation and low-level chlorination*” (Lin et al. 2016), “*Mechanisms of ultraviolet disinfection and chlorination of Escherichia coli: Culturability, membrane permeability, metabolism, and genetic damage*” (Xu et al. 2018), and “*Free radicals removing extracellular polymeric substances to enhance the degradation of intracellular antibiotic resistance genes in multi-resistant Pseudomonas Putida by UV/H*_*2*_*O*_*2*_* and UV/peroxydisulfate disinfection processes*” (Meng et al. 2022) focus on inactivating only one species of bacteria, limiting themselves to a model ARB, such as *B. subtilis* (Table 5), *E. coli* (Table SM4 and SM5), and *P. putida* (Table SM7).

There are several reasons for the inactivation studies of particular bacteria as models in scientific research, which may be (i) these species of bacteria are easy to grow in the laboratory and can be obtained in large numbers; (ii) they are considered indicators of microbial contamination and are commonly used to assess the effectiveness of disinfection processes; (iii) in the case of *E. coli* and *P. putida*, they are known to carry antibiotic resistance genes and are therefore important for evaluating the elimination of antibiotic resistance in water treatment processes (Bower et al. 2005; Chun et al. 2022; He et al. 2019). However, it is important to note that the microorganisms used in laboratory models or assays may not fully represent the microbial diversity present in wastewater or other aquatic environments. Therefore, this limitation must be considered when interpreting the results of bacterial inactivation studies using these models.

In the specific case of the article “*Combination of flow cytometry and molecular analysis to monitor the effect of UVC/H*_*2*_*O*_*2*_* vs UVC/H*_*2*_*O*_*2*_*/Cu-IDS processes on pathogens and antibiotic resistant genes in secondary wastewater effluents*” when carrying out research on water samples real residual, take advantage of the evaluation of a wide variety of species of bacteria (Di Cesare et al. 2020). They highlight that they inactivated putative pathogenic bacteria of the genera *Acinetobacter*, *Aeromonas*, *Bacillus*, *Bacteroides*, *Citrobacter*, *Enterobacter*, *Enterococcus*, *Escherichia-Shigella*, *Legionella*, *Morganella*, *Pantoea*, *Prevotella*, *Proteus*, *Pseudomonas*, *Serratia*, *Streptococcus*, and *Treponema*. Being the only article selected for the systematic analysis section that eliminates a number of bacterial species that can significantly represent the microbial diversity present in wastewater, reasons why it is the article with the greatest strengths in this lens and no disadvantages.

Focusing the analysis on the information in the selected patents, it was observed that two of the five patents detail the types of bacteria or microorganisms they eliminate. In patent CN111620493A, they eliminate heterotrophic bacteria in general, a classification that includes species within the genera *Pseudomonas*, *Aeromonas*, *Alcaligenes*, *Acinetobacter*, *Klebsiella*, *Flavobacterium*, *Chromobacterium*, and others. The reduction of *Legionella* growth and the eradication of *Listeria* are also mentioned, which are usually present and in high concentration in feedlot waters (Table 5).

Additionally, patent WO2020019047A1 extends the elimination to different types of microorganisms, effectively eliminating bacteria, viruses, fungi, spores, and more commonly pathogenic microorganisms, including *Campylobacter* spp*.*, *Escherichia coli*, *Legionella pneumophila*, *Pseudomonas aeruginosa*, *Salmonella*, *Staphylococcus aureus*, *Aspergillus niger*, and *Candida albicans*, among others (Table SM10). On the other hand, in the three remaining patents (CN111620493A—Table SM8, CN111056701A—SM9, and KR20210042540A—SM11), they did not specify the microorganisms or species of bacteria eliminated, writing bacteria as a general term. This latter situation may be since inventions tend to limit information about the microorganisms to be eliminated because detailed disclosure of the identity and characteristics of these microorganisms could limit the scope of the patent in terms of its patentability and commercially. Therefore, in order to maximize their commercial reach and profit potential, patent holders often refrain from disclosing detailed information about the microorganisms targeted for inactivation (Ernst 2003; McGee 1966; Schmidt 2013).

In scientific and technological production, it is important to increase the number and types of bacteria inactivated by UV, H_2_O_2_, and/or Cl_2_ disinfection systems, individually and in combination, because the diversity of microorganisms found in water can vary by geographic region and water use (Posselt et al. 2020; Wu et al. 2019; Zhang et al. 2019). In addition, some microorganisms may be more resistant to certain disinfection methods, so it is necessary to evaluate the effectiveness of different treatment combinations to ensure water safety (Luo et al. 2021; Masjoudi et al. 2021; Phattarapattamawong et al. 2021). In addition, with the emergence of new threats to public health, such as the spread of emerging diseases, antimicrobial resistance, and the presence of chemical contaminants in water, it is necessary to evaluate the ability of disinfection systems to eliminate these threats (Akhbarizadeh et al. 2020; Khan et al. 2022). Therefore, expanding the number and types of bacteria inactivated by UV, H_2_O_2_, and/or Cl_2_ disinfection systems, individually or in combination, can help improve water quality and ensure public health protection.

#### Development of the fourth lens: associated ARG removed

Within the systematic analysis guided to answer the fourth lens, it was found that four of the five articles reviewed eliminated different genes. In terms of ARGs, in the article “*Degradation and deactivation of bacterial antibiotic resistance genes during exposure to free chlorine, monochloramine, chlorine dioxide, ozone, ultraviolet light and hydroxyl radicals*,” the *blt* gene was deleted from the *bltR-blt-bltD* genome segment with an *acfA* mutation encoding efflux-mediated constitutional resistance to a wide variety of antibiotics such as fluoroquinolones, chloramphenicol, doxorubicin, and acriflavine (Table 5) (He et al. 2019). In the article “*Reduction in horizontal transfer of conjugative plasmid by UV irradiation and low-level chlorination*,” they eliminated a greater number of genes, being *aphA*, *bla*, *tetA*, and *tetR* genes associated with resistance to kanamycin, ampicillin, and tetracycline, respectively (Table SM4). And in addition, they eliminated important genes in the horizontal transfer of genes, which are *FlgC*, *ompF*, and *TraG*, genes that encode proteins necessary in the horizontal transfer of genes between bacteria (Lin et al. 2016).

Similarly, with an amount of ARG removed in the article “*Combination of flow cytometry and molecular analysis to monitor the effect of UVC/H*_*2*_*O*_*2*_* vs UVC/H*_*2*_*O*_*2*_*/Cu-IDS processes on pathogens and antibiotic resistant genes in secondary wastewater effluents*,” they remove the *tet-A*, *qnrS*, and *sul2* genes, which confer resistance to tetracycline, quinolones and sulfonamides, respectively (Table SM6) (Di Cesare et al. 2020). Finally, the article “*Free radicals removing extracellular polymeric substances to enhance the degradation of intracellular antibiotic resistance genes in multi-resistant Pseudomonas Putida by UV/H*_*2*_*O*_*2*_* and UV/peroxydisulfate disinfection processes*” was the one that presented a greater number and types of ARGs, which, after the analysis, allowed us to determine which is the article with the most strengths and no weaknesses in terms of the way to select, present, eliminate, and analyze the ARGs. Three ARGs were eliminated that encode plasmids (eARG) which are *tetA -01* (provides resistance to tetracycline), *aac6-lb*, and *strA* (provides resistance to aminoglycosides), in addition to four ARGs that encode chromosomes (iARG) that are *acrB* (provides resistance to fluoroquinolones), *tetA -02* (provides resistance to tetracycline), *sulI* (provides resistance to sulfonamides), and *mexF* (provides resistance to penicillins) (Table SM7) (Meng et al. 2022).

The article “*Mechanisms of ultraviolet disinfection and chlorination of Escherichia coli: Culturability, membrane permeability, metabolism, and genetic damage*” did not record the type of ARGs they eliminated (Table SM5). In fact, in the article, they evaluated the damage to the genetic material, focusing on the genes necessary for the replication of the microorganism and for DNA damage repair, following damage to the mRNA of the single-stranded DNA binding protein (*ssb*), the protein initiator of chromosome replication (*dnaA*), glutamate decarboxylase (*gadA*), in the SOS response, and DNA repair (*RecA*) (Xu et al. 2018). The genes evaluated in this study are important because they are involved in processes vital to the survival and reproduction of bacteria, so deletion of these genes can have a detrimental effect on bacteria, which can be beneficial in the elimination of pathogenic microorganisms. In addition, deletion of these genes may prevent the development of resistance to antibiotics. Therefore, evaluation of the deletion of these genes is significant to ensure the effectiveness of disinfection systems and prevent the spread of infectious diseases (Deborde and Gunten 2008; Dong et al. 2020; Jungfer et al. 2007; Wang et al. 2020; Xu et al. 2018). However, since the direct damage to ARGs, which can remain in the medium and be easily assimilated by other nearby non-resistant bacteria, was not evaluated, the results were not considered as important bases to solve the fourth objective of this work (ARGs removed).

Regarding the patents, four of the five patents did not mention genes specifically; they mention the term “gene deletion” or “damage to genetic material” in general. Only patent CN111056701A “*Method and special equipment for removing antibiotic-resistant bacteria and resistant genes in sewage*” specified the removal of *tet-A*, *tet-C*, *tet-M*, *tet-W*, and *tet-X* (tetracycline resistance genes), and *sul1* and *sul2* (resistance to sulfonamides) (Table SM9).

In general, the ambiguity with which patents refer to causing damage to genetic material can be attributed to two reasons: (i) the removal of ARGs is not the primary goal of proprietary disinfection systems, but rather the inactivation of pathogenic microorganisms and the prevention of the spread of infectious diseases. Therefore, the removal of ARGs may not be considered an important or relevant feature for the commercial use of the disinfection system (Giannakis et al. 2021; Shinde et al. 2023; Tansel 2010). (ii) As noted in previous lenses, patents may be limited in the amount of information they can provide, as full disclosure may affect the legal protection and exclusivity of the patented technology. Providing too much detail about removed ARGs could limit the breadth of the patent, potentially reducing its commercial value and attractiveness to investors (Blackman 1995; Ernst 2003; McGee 1966; Schmidt 2013).

The removal of the ARG genes mentioned in the reviewed articles is significant because they are genes that confer resistance to various antibiotics and therefore pose a public health risk. Antibiotic resistance is a growing global threat that hinders the treatment of bacterial infections and increases morbidity and mortality worldwide. Deleting ARGs genes is therefore an effective way to reduce the spread of antibiotic resistance in the environment (Echeverry-Gallego et al. 2023; Iwu et al. 2020; Manyi-Loh et al. 2018; Martínez-Pachón et al. 2021).

The ARGs selected in the documents analyzed confer resistance to antibiotics widely used in clinical practice and in animal production, such as tetracyclines, sulfonamides, quinolones, and penicillins, among others (Cai et al. 2021; Manyi-Loh et al. 2018; Robles-Jimenez et al. 2021). In addition, these ARGs represent a common set of resistance genes that have been identified in a wide variety of bacterial species, both pathogenic and non-pathogenic. Deleting these genes can help reduce the ability of bacteria to acquire and transmit antibiotic resistance, which is particularly important in settings where large amounts of antimicrobial are used, such as in animal production and healthcare (Dodd 2012; Echeverry-Gallego et al. 2023; Hurd & Malladi 2008).

It is significant to note that these studies may have limitations in selecting specific genes for deletion, as not all potential resistance genes can be evaluated in a single study. Also, some resistance genes may be more difficult to remove than others due to their location on plasmids or the bacterial chromosome, which may require different removal strategies. Similarly, there may be other resistance mechanisms not associated with ARG genes that may limit the effectiveness of ARG gene knockdown. Therefore, comprehensive and multifaceted approaches are needed to address the problem of antibiotic resistance in the environment.

#### Development of the fifth objective: efficiency of the disinfection system

When evaluating the elimination of ARBs and/or ARGs in disinfectant articles, it is important to demonstrate the effectiveness of the established disinfection systems (UV, H_2_O_2_, and/or Cl_2_ individually or in combination) in reducing the bacterial load and eliminating pathogens from a given environment or substrate. This increases the scientific scope of the article or the commercial scope of the patent. In addition, comparing the effectiveness of different disinfection systems can help determine the optimal operating parameters, such as dose, exposure time, pH, and concentration of disinfectants used, to ensure complete and effective elimination of microorganisms and ARGs present in the evaluated environment or substrate. This information can then be used to determine which disinfection system is most appropriate for treating a particular type of water or environment (Bilińska et al. 2016; Bolton et al. 1996; Comninellis et al. 2008; Suty et al. 2004). This objective is established as one of the most important in this study.

Consequently, it was observed that in the articles, they demonstrated efficiency in terms of ARB inactivation and ARG removal as a decrease in gene copies, and in some articles, they detailed more evaluations of ARB membrane and protein damage. Thus, in the article “*Degradation and Deactivation of Bacterial Antibiotic Resistance Genes during Exposure to Free Chlorine, Monochloramine, Chlorine Dioxide, Ozone, Ultraviolet Light, and Hydroxyl Radical*,” they evaluated different disinfection methods (FAC, O_3_, UV, NH_2_Cl, and ClO_2_), and it was found that the first three methods were highly efficient (more than 90% removal of ARG and iARG), while the last two methods were inefficient for their complete removal (Table 5) (He et al. 2019). In the article “*Reduction in horizontal transfer of conjugative plasmid by UV irradiation and low-level chlorination*,” they evaluated the efficiency of exposure to UV and chlorine doses in reducing the frequency of ARGs transfer in donor and recipient bacteria. The frequency of transfer was found to be significantly reduced after exposure to UV and chlorine doses at different doses and concentrations (Table SM4) (Lin et al. 2016).

In the third studym “*Mechanisms of ultraviolet disinfection and chlorination of Escherichia coli: Culturability, membrane permeability, metabolism, and genetic damage*,” they evaluated the efficiency of two disinfection systems in inactivating *E. coli.* The UV and chlorine systems were highly efficient, with complete inactivation of *E. coli* observed at specific doses of UV and chlorine. In addition, genetic damage and decreased production of key proteins and metabolites were observed (Xu et al. 2018). In the fourth study “*Combination of flow cytometry and molecular analysis to monitor the effect of UVC/H*_*2*_*O*_*2*_* vs UVC/H*_*2*_*O*_*2*_*/Cu-IDS processes on pathogens and antibiotic resistant genes in secondary wastewater effluents*,” two disinfection systems were compared (UV-C/H_2_O_2_/Cu-IDS and UV-C/H_2_O_2_) in terms of bacterial inactivation and ARG dynamics under different environmental conditions. The UV-C/H_2_O_2_/Cu-IDS system was found to be more efficient in bacterial inactivation under ambient conditions due to the modification (Table SM6) (Di Cesare et al. 2020). Finally, in the article “*Free radicals removing extracellular polymeric substances to enhance the degradation of intracellular antibiotic resistance genes in multi-resistant Pseudomonas Putida by UV/H*_*2*_*O*_*2*_* and UV/peroxydisulfate disinfection processes*,” they evaluated three disinfection systems (UV, UV/H_2_O_2_, and UV/PDS), finding that the UV/H_2_O_2_ and UV/PDS systems were highly efficient in the inactivation of *P. putida MX-2* and in the elimination of antibiotic resistance genes carried by this bacterium (Table SM7) (Meng et al. 2022).

According to the information provided by the five articles analyzed, specific working doses or concentrations of the three disinfectants were found as application trends for greater efficiency in the disinfection processes of biologically contaminated water. Thus, for the use of UV radiation, a decrease in the frequency of transfer from donor to recipient bacteria was found after exposure to UV doses between 5 and 20 mJ/cm^2^ (Table 5 y SM4) (He et al. 2019; Lin et al. 2016). Furthermore, complete inactivation of *E. coli* was obtained with UV doses between 35 and 80 mJ/cm^2^ (Table SM5) (Xu et al. 2018). Therefore, it is suggested as the optimal dose to use UV between 5 and 80 mJ/cm^2^ to achieve optimal efficiency in the elimination of ARBs and ARGs. Establishing a dose through systematic analysis can optimize the use of UV disinfection technology by providing a range of doses that may be effective. However, the optimal dose may vary depending on factors specific to the treatment system, such as water quality and contaminant concentration, so this recommendation should be used as a general guide and not as a hard and fast rule. The optimal dose recommendation is based on information from specific studies, and results may vary depending on the specific conditions of each disinfection system. Therefore, there are still several gaps and challenges in establishing the best doses of UV radiation for its future application on a larger scale.

In the case of the results where they implement chlorine, they indicate that a dose of chlorine between 0.3 and 0.5 mg/L can reduce the frequency of transfer of donor bacteria to recipients (Table SM4) (Lin et al. 2016). In addition, the complete elimination of *E. coli* was achieved with a concentration of 5 mg/L of free chlorine in contact for 10 min (Table SM5) (Xu et al. 2018). Therefore, it is suggested to use a free chlorine concentration between 0.3 and 5 mg/L and a contact time of at least 10 min to achieve optimal efficiency in the removal of ARBs and ARGs. In this case, the chlorine range considered as a trend in these documents is the one commonly used to disinfect drinking water and reduce the microbial load in treated wastewater before it is discharged to the environment (Fattoruso et al. 2014; Torretta et al. 2018). However, it is important to note that chlorine is a powerful disinfectant and can have negative effects on aquatic life and other organisms at very high concentrations. Additionally, the use of chlorine can create toxic by-products, such as trihalomethanes, if not handled properly. Therefore, measures such as appropriate dosing, handling, and disposal procedures should be implemented to ensure the safe use of chlorine in water and wastewater treatment and to minimize any negative impact on the environment (Carpinteiro et al. 2019; Espinosa-Barrera et al. 2021; He et al. 2022; Jia et al. 2023; Khalit and Tay 2017).

Regarding the implementation of H_2_O_2_, the combined UV/H_2_O_2_ system showed the highest efficiency in the inactivation of *P. putida MX-2* (together with a UV/PDS system), degradation of transported i-ARG, membrane damage cell, and EPS degradation of *P. putida MX-2*. The optimal operating conditions for these systems were 254 nm UV light at a confluence of 320 mW/cm^2^, and a concentration of 0.3 mM H_2_O_2_, at any tested pH (3, 7, or 10) (Table SM7) (Meng et al. 2022). Therefore, this UV/H_2_O_2_ system with those optimal conditions could be used to achieve optimal efficiency in the removal of bacteria and ARG.

About what was reported within the technological production, there is no detailed data of the system that generates high efficiencies, beyond what is described in “General information obtained in the patents” section of the general information on the conditions and components of disinfection systems. However, they briefly describe the efficiency obtained in water treatment, where in patent US2013087504A1, they reduced more than 90% and, in some cases, it reaches up to 99.9% depending on the heterotrophic bacteria. In the case of the *Legionella* bacteria, the reduction in concentration exceeded 60% and, in some cases, reached up to 85%. Finally, they observed the significant reduction in the colonies of bacteria, including coliforms, *Staphylococcus aureus*, *Escherichia coli*, and *Listeria*.

In patent CN111620493A, they observed that the system allows water disinfection efficiencies ranging from 32.64 to 99.30% in systems using a single disinfectant such as UV or H_2_O_2_, while treatments using a combination of UV/H_2_O_2_ disinfectants reach 100% efficiency in 10 min of treatment. The most efficient treatment is treatment 8, which uses an H_2_O_2_ concentration of 40 mg/L. In general, the disinfection efficiency is higher for sulfa resistance genes than for tetracycline resistance genes (Table SM8).

In patent CN111056701A, they describe that with the disinfection system, they were able to reduce the concentration of fecal coliform bacteria between 1.7 × 10^4^ and 2.2 × 10^4^ MPN/L; they reduced COD ≤ 100 mg/L, ammoniacal nitrogen ≤ 100 mg/L, and turbidity at a level of 2 to 3 NTU (Table SM9). Finally, in patent WO2020019047A1, they only highlight that they are highly effective against a wide range of bacteria, viruses, fungi, and spores, and they even mention that the disinfection system has strong disinfection power even in the presence of biofilm, since it penetrates on the cell membrane of the target microorganism, but they do not imply percentages, tables, or figures related to efficiency (Table SM10).

It is important to note that none of the patents show various ways to demonstrate and illustrate efficacy in the removal of ARBs and ARGs, nor do they include figures, tables, or statistical analyses related to efficacy. There are several reasons why patents may not include efficacy data related to the removal of ARBs or ARGs, such as figures, tables, or statistical analyses. First, patents may be more focused on describing the process or technology than presenting detailed efficacy test results. In addition, test results may be subject to variability due to various factors, such as water quality or bacterial concentration, making it more difficult to present the data in a conclusive manner. Finally, efficacy data may be considered confidential information by the patent holder and therefore cannot be shared publicly (Azoulay et al. 2019; Blackman 1995; Meyer 2004; van Rijn and Timmis 2023; Tilmann 2018).

Due to the wide range of efficiencies observed in these systems, several aspects can be specified: (i) combined UV/H_2_O_2_ systems have shown greater efficiencies in the removal of ARBs and ARGs compared to individual UV systems or compared to the use of Cl_2_ in other studies. The addition of H_2_O_2_ to the UV-treated water stream increases the formation of highly reactive oxidizing species, such as hydroxyl radicals, which can degrade a wide range of contaminants, as mentioned in Lens 1 (“The low scientific-technological interest on the combined systems H_2_O_2_/Cl_2_ and UV/H_2_O_2_/Cl_2_” section). Therefore, combined UV/H_2_O_2_ systems are a promising alternative for water treatment and environmental removal of ARBs and ARGs. Other untested combined systems, such as H_2_O_2_/Cl_2_ and UV/H_2_O_2_/Cl_2_, may also be effective for selected applications (Belghit et al. 2020; Berruti et al. 2022; Djaballah et al. 2023). (ii) Disinfection processes based on UV, H_2_O_2_, and Cl_2_ have great versatility to work in a wide range of pH. The UV process is pH independent, meaning that it is effective in any pH range, from acidic to alkaline. The H_2_O_2_ process is also relatively pH-independent, although it degrades more quickly in alkaline media. Chlorine works better in slightly acidic media, but can be used in alkaline media with adjustments in dosage and contact time (Chu et al. 2022; Pai and Wang 2022; Shah et al. 2011; Tian et al. 2020; Wang et al. 2019a, b, c). (iii) These systems are capable of inactivating a wide range of microorganisms through the combination of AOPs and photolysis (H_2_O_2_, Cl_2_, and UV). AOPs, or advanced oxidation processes, use highly reactive oxidizing species to break down contaminants, while photolysis uses UV radiation to break down the DNA of microorganisms. Together, they destroy microorganisms by disrupting the cell membrane and degrading essential biological molecules, such as extracellular polymeric substances (EPS). In addition, the combination of these processes has been shown to be effective in eliminating ARGs, which are a major concern due to their ability to transfer antibiotic resistance to other bacteria (Di Cesare et al. 2020; He et al. 2019; Lin et al. 2016; Meng et al. 2022; Xu et al. 2018). (iv) These systems have the advantage of being highly versatile and effective in a wide range of aqueous matrices with varying characteristics. They have been shown to be effective in removing bacteria and ARG from ultrapure water, wastewater, and surface and groundwater with varying levels of turbidity, alkalinity, and hardness. The effectiveness of these systems in various aqueous matrices makes them a promising tool for improving water quality. They can help reduce the spread of infectious diseases associated with antibiotic-resistant bacteria in various applications (Ferro et al. 2016; Hassen et al. 2000; Liang et al. 2022; Mamane 2008; Zhong et al. 2019).

### Development of the sixth lens: cost analysis of the disinfection system

None of the manuscripts (Table 5, Table SM4–SM7) and patents (Tables SM8–SM11) selected in the systematic analysis section performed a cost analysis for the UV, H_2_O_2_, and/or Cl_2_ disinfection systems, individually or in combination. This is despite the fact that a cost analysis is important to determine whether the investment in a disinfection system is profitable in the long term and whether its future implementation on a larger scale is feasible. In cases where a higher quality of treated water (effluent) with greater removal of ARBs and ARGs is required, the associated costs may be higher. Modifications to the system may be required to increase its applicability in these scenarios (Buthiyappan et al. 2015; Costa et al. 2021; Sgroi et al. 2021; Sichel-Crespo et al. 2022).

In the specific case of patents, it is necessary to determine patentability, which refers to the ability of an invention to meet the patentability requirements set forth in intellectual property law. These include novelty, inventiveness, and industrial applicability (Mishra 2014; Webber 2003). Therefore, cost reduction may not be directly related to these patentability criteria. However, it may make a system more accessible and attractive to consumers, thereby increasing demand and sales and making it more competitive in the marketplace (Azoulay et al. 2019; Ernst 2003; Hall 2022).

Among the important costs to increase the competitiveness of these systems in the marketplace, it is necessary to consider the costs associated with installing, operating, maintaining, and monitoring the disinfection system to ensure that it is functioning properly and in compliance with applicable regulations. This includes operational costs such as energy consumption and waste disposal (Chuang and Huang 2016; Mahamuni and Adewuyi 2010; Sichel-Crespo et al. 2022; Swift et al. 2000).

The evaluation of the disinfection system must also consider the potential benefits of eliminating ARBs and ARGs to determine the cost–benefit ratio for consumers. Although these benefits can be difficult to quantify, they include reducing the spread of disease and improving public health (Chen et al. 2021; Danguy and van Pottelsberghe de la Potterie 2011). Therefore, omitting a cost analysis when evaluating a disinfection system for the removal of ARBs and ARGs using UV, H_2_O_2_, and/or Cl_2_ systems (individually or in combination) can create a significant gap in informed decision-making. Without proper cost evaluation, researchers and/or consumers may not fully understand the costs and benefits of the disinfection system and may make suboptimal implementation decisions.

## Conclusion

### Overview

Our analysis highlights the significant interest in UV, H_2_O_2_, and/or Cl_2_-based surface and environmental disinfection technologies. The focus on patent publication suggests a competitive landscape with companies investing heavily in protecting their intellectual property. In addition, China and the US are leading the scientific and technological production in this field. The inclusion of specific terms such as *E. coli* and antibiotic resistance indicates a growing concern about the elimination of ARBs and ARGs.

The analysis shows that China has the highest number of joint publications with other countries, while Asian countries collaborate more with each other than with European or American countries in ARB and ARG removal research. The most popular approach in this research is the combined use of UV and H_2_O_2_. The selected manuscripts and patents explore various systems of interest, including UV, Cl_2_, UV/H_2_O_2_, and UV/Cl_2_ combinations, as well as other advanced oxidation processes commonly used in wastewater disinfection. The majority of articles in the dataset were published in high-impact water and environmental science journals, indicating a growing concern in the scientific community about the risks of spreading ARBs and ARGs through water. The patents filed in different countries reflect a focus on developing innovative technologies to control harmful microorganisms that can affect both humans and animals, with a particular emphasis on addressing the problem of harmful microorganisms in the agricultural industry. Overall, the results underscore the urgent need to develop effective and sustainable technologies to control the spread of ARBs and ARGs in water.

The combination of UV, H_2_O_2_, and/or Cl_2_ technologies may be a promising strategy to improve disinfection efficacy and reduce the formation of toxic by-products, but more research is needed to determine the best combination for different applications and to address the challenges associated with its implementation. In assessing the current state of research in this area, it is important to consider the various factors that influence scientific and technological production.

Each system has its strengths and weaknesses in terms of its ability to remove ARBs and ARGs in different aqueous matrices. Some trends have been observed where the use of UV radiation alone focuses mainly on ARB removal, while the addition of H_2_O_2_ or Cl_2_ (in different combinations) enhances ARG removal, making these systems effective in removing both ARBs and ARGs. In addition, the combination of UV and H_2_O_2_ appears to be particularly effective in removing ARGs, including those that are resistant to multiple drugs.

### Future directions

In the bibliographic and systematic review of the scientific-technological production carried out, some gaps were found in the scientific-technological production of combined UV/H_2_O_2_/Cl_2_ disinfection systems. Including the lack of studies that evaluate the effectiveness of these systems in the elimination of ARBs and ARGs in unison, as well as increasing the amount and types of these contaminants. There is lack of studies evaluating the effects of reaction by-products on the quality of treated water. Most of the studies have been conducted at the laboratory level and have not been fully evaluated under pilot-scale conditions or at the industrial level. Overall, this study serves as a starting point for future research aimed at developing effective and sustainable disinfection technologies that can reduce the spread of infectious diseases and prevent the emergence of antibiotic resistance.

Despite these gaps, there are promising prospects in the use of combined UV/H_2_O_2_/Cl_2_ disinfection systems. These systems have several advantages over conventional disinfection systems, such as the ability to remove a wide range of contaminants, including ARBs, fungi or viruses, recorded in other jobs, microorganisms resistant to conventional disinfectants, and the ability to reduce the formation of toxic disinfection byproducts. In addition, UV/H_2_O_2_/Cl_2_ technology can be scalable and suitable for implementation in large-scale water treatment plants, still facing challenges in implementing these systems, including the complexity of system design and operation, the need for constant monitoring and maintenance, and the costs associated with installing and operating the systems. These challenges deserve thorough scientific investigation to pave the way for future technological applications.

### Supplementary Information

Below is the link to the electronic supplementary material.

Supplementary file1 (DOCX 491 KB)

## Data Availability

All data generated or analyzed during this study are included in this published article (and its supplementary information files).
