# Supplementary material for: Systematic analysis of the scientific-technological production on the use of the UV, H2O2, and/or Cl2 systems in the elimination of bacteria and associated antibiotic resistance genes
Source: Environ Sci Pollut Res Int. 2024 Jan 2;31(5):6782–814. doi: 10.1007/s11356-023-31435-2 (PMC10821820; doi:10.1007/s11356-023-31435-2)
Supplement: Supplementary file 1 — Supplementary file1 (DOCX 491 KB) [file 11356_2023_31435_MOESM1_ESM.docx]

**SUPPLEMENTARY MATERIAL**

**Systematic Analysis of the Scientific-Technological Production on the Use of the UV, H_2_O_2_, and/or Cl_2_ Systems in the Elimination of Bacteria and Associated Antibiotic Resistance Genes**

Paula Andrea Espinosa-Barrera^1,2^, Marcela Gómez-Gómez^1^, Javier Vanegas^1^, Fiderman Machuca-Martínez^3^, Ricardo Antonio Torres-Palma^4^, Diana Martínez-Pachón^1^, Alejandro Moncayo-Lasso^1*^

*^1^ Grupo de Investigación en Ciencias Biológicas y Químicas, Facultad de Ciencias, Universidad Antonio Nariño, Bogotá D.C., Colombia.*

*^2^ Doctorado en Ciencia Aplicada (DCA), Universidad Antonio Nariño, Bogotá D.C., Colombia.*

*^3^ Centro de Excelencia en Nuevos Materiales (CENM), Universidad del Valle, Calle 13 Nº100-00, Cali, Colombia.*

*^4^ Grupo de Investigación en Remediación Ambiental y Biocatálisis (GIRAB), Instituto de Química, Facultad de Ciencias Exactas y Naturales, Universidad de Antioquia UdeA, Calle 70 No. 52-21, Medellín, Colombia.*

**Corresponding author:**

* Alejandro Moncayo - Lasso (e-mail: amoncayo@uan.edu.co)

**Supplementary Tables**

**Table SM1.** Portfolio construction: search input keywords and number of documents found as published material on the topic between 2011 and 2022.

| **No.** | **Keywords** | **No. of articles** | **No. of patents** |
| --- | --- | --- | --- |
| 1 | ("chlorine" OR "Chlorination" OR "Cl") AND ("H2O2" OR "peroxide") AND ("Ultraviolet" OR "UV") | 29226 | 156661 |
| 2 | ("chlorine" OR "Chlorination" OR "Cl") AND ("peroxide") AND ("Ultraviolet" OR "UV") | 28733 | 155159 |
| 3 | ("chlorine" OR "Chlorination" OR "Cl") AND ("H2O2" OR "peroxide") AND ("Ultraviolet") | 9845 | 82439 |
| 4 | ("wastewater treatment") AND ("disinfection") AND ("chlorine") AND ("gene" OR "bacteria") AND ("ultraviolet" OR "UV") AND ("hydrogen peroxide" OR "H2O2") | 16 | 172 |
| 5 | ("wastewater treatment") AND ("disinfection") AND ("chlorine") AND ("gene" OR "bacteria") AND ("ultraviolet" OR "UV") AND ("hydrogen peroxide" OR "H2O2") | 9 | 75 |
| 6 | ("disinfection") AND ("chlorine") AND ("gene" OR "bacteria") AND ("ultraviolet" OR "UV") AND ("hydrogen peroxide" OR "H2O2") | 43 | 412 |
| 7 | ("gene") AND ("chlorine" OR "Chlorination" OR "Cl") AND ("H2O2" OR "peroxide") AND ("Ultraviolet" OR "UV") | 31 | 39994 |
| 8 | ("bacteria") AND ("chlorine" OR "Chlorination" OR "Cl") AND ("H2O2" OR "peroxide") AND ("Ultraviolet" OR "UV") | 98 | 13610 |

**Table SM2.** Final portfolio of peer-reviewed documents with corresponding citation counts.

| **Articles** | | |
| --- | --- | --- |
| **Title** | **Reference** | **No. of citations** |
| Inactivation of *Escherichia coli*, Bacteriophage MS2, and *Bacillus Spores* under UV/H_2_O_2_ and UV/Peroxydisulfate Advanced Disinfection Conditions | [(P. Sun et al., 2016)](https://sciwheel.com/work/citation?ids=12090743&pre=&suf=&sa=0) | 164 |
| Inactivation efficiency of plasmid-encoded antibiotic resistance genes during water treatment with chlorine, UV, and UV/H_2_O_2_ | [(Yoon et al., 2017)](https://sciwheel.com/work/citation?ids=12090744&pre=&suf=&sa=0) | 153 |
| Degradation and Deactivation of Bacterial Antibiotic Resistance Genes during Exposure to Free Chlorine, Monochloramine, Chlorine Dioxide, Ozone, Ultraviolet Light, and Hydroxyl Radical | [(H. He et al., 2019)](https://sciwheel.com/work/citation?ids=14174398&pre=&suf=&sa=0) | 115 |
| Inactivation and regrowth of multidrug resistant bacteria in urban wastewater after disinfection by solar-driven and chlorination processes | [(Fiorentino et al., 2015)](https://sciwheel.com/work/citation?ids=4819185&pre=&suf=&sa=0) | 110 |
| β-lactams resistance gene quantification in an antibiotic resistant *Escherichia coli* water suspension treated by advanced oxidation with UV/H_2_O_2_ | [(Ferro et al., 2017)](https://sciwheel.com/work/citation?ids=7663836&pre=&suf=&sa=0) | 94 |
| Reduction in horizontal transfer of conjugative plasmid by UV irradiation and low-level chlorination | [(Lin et al., 2016)](https://sciwheel.com/work/citation?ids=5727936&pre=&suf=&sa=0) | 74 |
| Mechanisms of ultraviolet disinfection and chlorination of *Escherichia coli*: Culturability, membrane permeability, metabolism, and genetic damage | [(Xu et al., 2018)](https://sciwheel.com/work/citation?ids=14174369&pre=&suf=&sa=0) | 68 |
| Inactivation dynamics of 222 nm krypton-chlorine excilamp irradiation on Gram-positive and Gram-negative foodborne pathogenic bacteria | [(Kang, Kim, & Kang, 2018)](https://sciwheel.com/work/citation?ids=14725070&pre=&suf=&sa=0) | 60 |
| Removal of *Microcystis aeruginosa* by UV/chlorine process: Inactivation mechanism and microcystins degradation | [(J. Sun, Bu, Deng, Shi, & Zhou, 2018)](https://sciwheel.com/work/citation?ids=14725071&pre=&suf=&sa=0) | 57 |
| Inactivation of chlorine-resistant bacterial spores in drinking water using UV irradiation, UV/Hydrogen peroxide and UV/Peroxymonosulfate: Efficiency and mechanism | [(Zeng et al., 2020)](https://sciwheel.com/work/citation?ids=11638299&pre=&suf=&sa=0) | 53 |
| Analysis of Hydroxyl Radicals and Inactivation Mechanisms of Bacteriophage MS2 in Response to a Simultaneous Application of UV and Chlorine | [(Rattanakul & Oguma, 2017)](https://sciwheel.com/work/citation?ids=12090737&pre=&suf=&sa=0) | 51 |
| Assessment of the UV/Chlorine Process in the Disinfection of *Pseudomonas aeruginosa*: Efficiency and Mechanism | [(L. Wang et al., 2021)](https://sciwheel.com/work/citation?ids=14725073&pre=&suf=&sa=0) | 46 |
| Inactivation of Bacteria by Peracetic Acid Combined with Ultraviolet Irradiation: Mechanism and Optimization | [(T. Zhang et al., 2020)](https://sciwheel.com/work/citation?ids=14725074&pre=&suf=&sa=0) | 41 |
| Kinetic modeling of *Escherichia coli* and *Enterococcus sp.* inactivation in wastewater treatment by photo-Fenton and H_2_O_2_/UV–vis processes | [(Rodríguez-Chueca, Ormad, Mosteo, & Ovelleiro, 2015)](https://sciwheel.com/work/citation?ids=14725076&pre=&suf=&sa=0) | 34 |
| Application of water-assisted ultraviolet light in combination of chlorine and hydrogen peroxide to inactivate *Salmonella* on fresh produce | [(S. Guo, Huang, & Chen, 2017)](https://sciwheel.com/work/citation?ids=14725078&pre=&suf=&sa=0) | 33 |
| Combination of aerosolized curcumin and UV-A light for the inactivation of bacteria on fresh produce surfaces | [(de Oliveira, Tikekar, & Nitin, 2018)](https://sciwheel.com/work/citation?ids=8948220&pre=&suf=&sa=0) | 33 |
| A comparative study of the disinfection efficacy of H_2_O_2_/ferrate and UV/H_2_O_2_/ferrate processes on inactivation of *Bacillus subtilis* spores by response surface methodology for modeling and optimization | [(Matin et al., 2018)](https://sciwheel.com/work/citation?ids=14725080&pre=&suf=&sa=0) | 29 |
| Effect of UV-C light or hydrogen peroxide wipes on the inactivation of methicillin-resistant *Staphylococcus aureus*, *Clostridium difficile* spores and *norovirus surrogate* | [(Wallace, Ouellette, & Jean, 2019)](https://sciwheel.com/work/citation?ids=11638293&pre=&suf=&sa=0) | 28 |
| UV/Chlorine Process: An Efficient Advanced Oxidation Process with Multiple Radicals and Functions in Water Treatment | [(K. Guo et al., 2022)](https://sciwheel.com/work/citation?ids=14725084&pre=&suf=&sa=0) | 28 |
| Combination of flow cytometry and molecular analysis to monitor the effect of UVC/H_2_O_2_ vs UVC/H_2_O_2_/Cu-IDS processes on pathogens and antibiotic resistant genes in secondary wastewater effluents | [(Di Cesare et al., 2020)](https://sciwheel.com/work/citation?ids=14174370&pre=&suf=&sa=0) | 25 |
| Inactivation of a wild isolated *Klebsiella pneumoniae* by photo-chemical processes: UV-C, UV-C/H_2_O_2_ and UV-C/H_2_O_2_/Fe^3+^ | [(Aguilar et al., 2018)](https://sciwheel.com/work/citation?ids=14725088&pre=&suf=&sa=0) | 19 |
| Inactivation of *Bacillus subtilis* Spores Using Various Combinations of Ultraviolet Treatment with Addition of Hydrogen Peroxide | [(Yiqing Zhang, Zhou, Zhang, & Tan, 2014)](https://sciwheel.com/work/citation?ids=14725090&pre=&suf=&sa=0) | 18 |
| Sterilization of hydrogen peroxide resistant bacterial spores with stabilized chlorine dioxide | [(Friedline et al., 2015)](https://sciwheel.com/work/citation?ids=14725091&pre=&suf=&sa=0) | 18 |
| UV-C/H_2_O_2_ heterogeneous photocatalytic inactivation of coliforms in municipal wastewater in a TiO_2_/SiO_2_ fixed bed reactor: a kinetic and statistical approach | [(Núñez-Núñez et al., 2018)](https://sciwheel.com/work/citation?ids=14725092&pre=&suf=&sa=0) | 14 |
| Factors affecting UV/H_2_O_2_ inactivation of *Bacillus atrophaeus* spores in drinking water | [(Yongji Zhang, Zhang, Zhou, & Tan, 2014)](https://sciwheel.com/work/citation?ids=14725093&pre=&suf=&sa=0) | 13 |
| A chemical, microbiological and (eco)toxicological scheme to understand the efficiency of UV-C/H_2_O_2_ oxidation on antibiotic-related microcontaminants in treated urban wastewater | [(Beretsou et al., 2020)](https://sciwheel.com/work/citation?ids=14174371&pre=&suf=&sa=0) | 12 |
| Free radicals removing extracellular polymeric substances to enhance the degradation of intracellular antibiotic resistance genes in multi-resistant *Pseudomonas Putida* by UV/H_2_O_2_ and UV/peroxydisulfate disinfection processes | [(Meng et al., 2022)](https://sciwheel.com/work/citation?ids=14174372&pre=&suf=&sa=0) | 9 |
| Bacterial community change and antibiotic resistance promotion after exposure to sulfadiazine and the role of UV/H_2_O_2_-GAC treatment | [(Huo, Zhao, Shi, Wang, & He, 2021)](https://sciwheel.com/work/citation?ids=14725097&pre=&suf=&sa=0) | 7 |
| Development of a percarbonate-enhanced Vacuum UV process for simultaneous fluoroquinolone antibiotics removal and fecal bacteria inactivation under a continuous flow mode of operation | [(Mohammadi, Moussavi, Yaghmaeian, & Giannakis, 2022)](https://sciwheel.com/work/citation?ids=14725099&pre=&suf=&sa=0) | 4 |
| Degradation and inactivation of chromosomal and plasmid encoded resistance genes/ARBs and the impact of different matrices on UV and UV/H_2_O_2_ based advanced oxidation process | [(Das, Bordoloi, Achary, Caldwell, & Suri, 2022)](https://sciwheel.com/work/citation?ids=14725064&pre=&suf=&sa=0) | 4 |
| **The total sum of citations in the portfolio of articles** | | **1414** |
| **Patents** | | |
| **Title** | **N°** | **No. of citations** |
| Use of peracetic acid/hydrogen peroxide and peroxide-reducing agents for treatment of drilling fluids, frac fluids, flowback water and disposal water | US10023484B2 | 39 |
| Water treatment device and methods of use | US2013087504A1 | 13 |
| Multi-Barrier System For Water Treatment | US2014353256A1 | 11 |
| Treating and recycling oilfield waste water | US2014374103A1 | 11 |
| Device for treating ship ballast water | CN102092814A | 9 |
| Apparatus and Method for Electro Disinfection | US10968120B2 | 8 |
| Oilfield wastewater treatment | US2017044035A1 | 7 |
| Ballast water sterilization device | WO2018147552A1 | 6 |
| Circulating water treatment method and equipment | CN104528957A | 6 |
| Highly efficient non-flush portable toilet recycling waste water | WO2017043928A1 | 5 |
| Method for sterilizing oil field sewage by cooperating electrochemical oxidation with ultraviolet and ultrasonic technology | CN102372386A | 5 |
| Multifunctional green pre-oxidation sewage treatment equipment and application method | CN113292185A | 5 |
| Electrochemically coupled dynamic membrane assembly and reactor for sewage treatment and application of electrochemically coupled membrane assembly and reactor | CN109354161A | 4 |
| Deodorizing equipment for sewage treatment | CN110451702A | 3 |
| Pig farm wastewater sterilization and disinfection method | CN107500397A | 3 |
| Device for removing antibiotic resistance bacteria and resistance genes in sewage | CN212559817U | 2 |
| Method and special equipment for removing antibiotic-resistant bacteria and resistant genes in sewage | CN111620493A | 2 |
| Treatment of fertigation water | US2020354239A1 | 2 |
| Wastewater composite disinfection process for harmless treatment of livestock and poultry died of illness | CN111056701A | 2 |
| Advanced water purification system using ultraviolet and activated carbon and advanced water purification method for using the same | KR101910483B1 | 1 |
| Apparatus for treating water | JP2011224424A | 1 |
| Technology for producing a disinfectant for neutralizing viruses, bacteria and other microorganisms | WO2020019047A1 | 1 |
| Industrial aquaculture source water disinfection system based on advanced oxidation | CN213446625U | 0 |
| Water Renewal Treatment System | AU2021202826A1 | 0 |
| Water treating apparatus based on advanced oxidation | KR20210042540A | 0 |
| **The total sum of citations in the portfolio of patents** | | **146** |

**Table SM3.** Filtered documents from the bibliometric analysis of the portfolio.

| **Articles** | | | | | | |
| --- | --- | --- | --- | --- | --- | --- |
| **Title** | **Reference** | **Year** | **No. of citations** | **Corresponding author** | **Index h** | **Journal** |
| Inactivation of *Escherichia coli*, Bacteriophage MS2, and *Bacillus Spores* under UV/H_2_O_2_ and UV/Peroxydisulfate Advanced Disinfection Conditions | [(P. Sun et al., 2016)](https://sciwheel.com/work/citation?ids=12090743&pre=&suf=&sa=0) | 2016 | 164 | Huang, Ching-Hua | 52 | Environmental Science and Technology |
| Inactivation efficiency of plasmid-encoded antibiotic resistance genes during water treatment with chlorine, UV, and UV/H_2_O_2_ | [(Yoon et al., 2017)](https://sciwheel.com/work/citation?ids=12090744&pre=&suf=&sa=0) | 2017 | 153 | Yunho Lee | 39 | Water Research |
| Degradation and Deactivation of Bacterial Antibiotic Resistance Genes during Exposure to Free Chlorine, Monochloramine, Chlorine Dioxide, Ozone, Ultraviolet Light, and Hydroxyl Radical | [(H. He et al., 2019)](https://sciwheel.com/work/citation?ids=14174398&pre=&suf=&sa=0) | 2019 | 115 | Dodd, Michael C. | 22 | Environmental Science and Technology |
| Inactivation and regrowth of multidrug resistant bacteria in urban wastewater after disinfection by solar-driven and chlorination processes | [(Fiorentino et al., 2015)](https://sciwheel.com/work/citation?ids=4819185&pre=&suf=&sa=0) | 2015 | 110 | Luigi Rizzo | 46 | Journal of Photochemistry and Photobiology B: Biology |
| β-lactams resistance gene quantification in an antibiotic resistant *Escherichia coli* water suspension treated by advanced oxidation with UV/H_2_O_2_ | [(Ferro et al., 2017)](https://sciwheel.com/work/citation?ids=7663836&pre=&suf=&sa=0) | 2017 | 94 | Luigi Rizzo | 46 | Journal of Hazardous Materials |
| Reduction in horizontal transfer of conjugative plasmid by UV irradiation and low-level chlorination | [(Lin et al., 2016)](https://sciwheel.com/work/citation?ids=5727936&pre=&suf=&sa=0) | 2016 | 74 | Xin Yu | 25 | Water Research |
| Mechanisms of ultraviolet disinfection and chlorination of *Escherichia coli*: Culturability, membrane permeability, metabolism, and genetic damage | [(Xu et al., 2018)](https://sciwheel.com/work/citation?ids=14174369&pre=&suf=&sa=0) | 2018 | 68 | Xiaochang C.Wang | 48 | Journal of Environmental Sciences |
| Inactivation dynamics of 222 nm krypton-chlorine excilamp irradiation on Gram-positive and Gram-negative foodborne pathogenic bacteria | [(Kang et al., 2018)](https://sciwheel.com/work/citation?ids=14725070&pre=&suf=&sa=0) | 2018 | 60 | Dong-Hyun Kang | 45 | Food Research International |
| Removal of *Microcystis aeruginosa* by UV/chlorine process: Inactivation mechanism and microcystins degradation | [(J. Sun et al., 2018)](https://sciwheel.com/work/citation?ids=14725071&pre=&suf=&sa=0) | 2018 | 57 | Shiqing Zhou | 42 | Chemical Engineering Journal |
| Inactivation of chlorine-resistant bacterial spores in drinking water using UV irradiation, UV/Hydrogen peroxide and UV/Peroxymonosulfate: Efficiency and mechanism | [(Zeng et al., 2020)](https://sciwheel.com/work/citation?ids=11638299&pre=&suf=&sa=0) | 2020 | 53 | Feng Ding | 1 | Journal of Cleaner Production |
| Assessment of the UV/Chlorine Process in the Disinfection of *Pseudomonas aeruginosa*: Efficiency and Mechanism | [(L. Wang et al., 2021)](https://sciwheel.com/work/citation?ids=14725073&pre=&suf=&sa=0) | 2021 | 46 | Jingyun Fang | 39 | Environmental Science and Technology |
| Inactivation of Bacteria by Peracetic Acid Combined with Ultraviolet Irradiation: Mechanism and Optimization | [(T. Zhang et al., 2020)](https://sciwheel.com/work/citation?ids=14725074&pre=&suf=&sa=0) | 2020 | 41 | Huang, Ching-Hua | 52 | Environmental Science and Technology |
| UV/Chlorine Process: An Efficient Advanced Oxidation Process with Multiple Radicals and Functions in Water Treatment | [(K. Guo et al., 2022)](https://sciwheel.com/work/citation?ids=14725084&pre=&suf=&sa=0) | 2022 | 28 | Jingyun Fang | 39 | Accounts of Chemical Research |
| Combination of flow cytometry and molecular analysis to monitor the effect of UVC/H_2_O_2_ vs UVC/H_2_O_2_/Cu-IDS processes on pathogens and antibiotic resistant genes in secondary wastewater effluents | [(Di Cesare et al., 2020)](https://sciwheel.com/work/citation?ids=14174370&pre=&suf=&sa=0) | 2020 | 25 | Luigi Rizzo | 46 | Water Research |
| A chemical, microbiological and (eco)toxicological scheme to understand the efficiency of UV-C/H_2_O_2_ oxidation on antibiotic-related microcontaminants in treated urban wastewater | [(Beretsou et al., 2020)](https://sciwheel.com/work/citation?ids=14174371&pre=&suf=&sa=0) | 2020 | 12 | Despo Fatta-Kassinos | 66 | Science of the Total Environment |
| Free radicals removing extracellular polymeric substances to enhance the degradation of intracellular antibiotic resistance genes in multi-resistant *Pseudomonas Putida* by UV/H_2_O_2_ and UV/peroxydisulfate disinfection processes | [(Meng et al., 2022)](https://sciwheel.com/work/citation?ids=14174372&pre=&suf=&sa=0) | 2022 | 9 | Guanyu Zheng | 22 | Journal of Hazardous Materials |
| Bacterial community change and antibiotic resistance promotion after exposure to sulfadiazine and the role of UV/H_2_O_2_-GAC treatment | [(Huo et al., 2021)](https://sciwheel.com/work/citation?ids=14725097&pre=&suf=&sa=0) | 2021 | 7 | Shouyang He | 5 | Chemosphere |
| Development of a percarbonate-enhanced Vacuum UV process for simultaneous fluoroquinolone antibiotics removal and fecal bacteria inactivation under a continuous flow mode of operation | [(Mohammadi et al., 2022)](https://sciwheel.com/work/citation?ids=14725099&pre=&suf=&sa=0) | 2022 | 4 | Stefanos Giannakis | 33 | Chemical Engineering Journal |
| Degradation and inactivation of chromosomal and plasmid encoded resistance genes/ARBs and the impact of different matrices on UV and UV/H_2_O_2_ based advanced oxidation process | [(Das et al., 2022)](https://sciwheel.com/work/citation?ids=14725064&pre=&suf=&sa=0) | 2022 | 4 | Rominder P.S.Suri | 24 | Science of the Total Environment |
| **Patents** | | | | | | |
| **Title** | **N°** | **Year** | **No. of citations** | **Patent type** | **Country** | **Inventors** |
| Use of peracetic acid/hydrogen peroxide and peroxide-reducing agents for treatment of drilling fluids, frac fluids, flowback water and disposal water | US10023484B2 | 2018 | 39 | Method | US | Victor Keasler, Renato de Paula, Li Junzhong, David D. Mcsherry, Brandon Herdt, Richard Staub, Robert J. Ryther |
| Water treatment device and methods of use | US2013087504A1 | 2015 | 13 | Apparatus | US | David Kolstad |
| Multi-Barrier System For Water Treatment | US2014353256A1 | 2014 | 11 | Method and apparatus | US | Martin Kaschek, Silvie Verplancke, Christof Granitz |
| Treating and recycling oilfield waste water | US2014374103A1 | 2014 | 11 | Method | US | Kushal Seth, Jenifer C. Lascano, Larry G. Hines |
| Device for treating ship ballast water | CN102092814A | 2013 | 9 | Apparatus | China | Liu Bingyan, Cao Xuelei, Cao Jingqiang |
| Oilfield wastewater treatment | US2017044035A1 | 2017 | 7 | Method | US | Timothy Underwood, Daryl D. McCracken, Larry G. Hines, Chrysta S. Scurlark |
| Ballast water sterilization device | WO2018147552A1 | 2018 | 6 | Apparatus | South Korea | Bum Mo Ahn, Seung Ho Park, Tae Hwan Song |
| Circulating water treatment method and equipment | CN104528957A | 2017 | 6 | Method and apparatus | China | Dong Gao, Junfeng Zhu, Li Yin, Xinquan Qiao, Qiuying Hu, Zhijian Zhou |
| Highly efficient non-flush portable toilet recycling waste water | WO2017043928A1 | 2017 | 5 | System | South Korea | Man - Seon Park |
| Multifunctional green pre-oxidation sewage treatment equipment and application method | CN113292185A | 2011 | 5 | Method and system | China | Min Wu, Yantao Qin |
| Electrochemically coupled dynamic membrane assembly and reactor for sewage treatment and application of electrochemically coupled membrane assembly and reactor | CN109354161A | 2019 | 4 | System | China | Zhiwei Wang, Qian Lei, Junjian Zheng, Xueye Wang, Qiaoying Wang, Zhichao Wu |
| Deodorizing equipment for sewage treatment | CN110451702A | 2019 | 3 | Apparatus | China | Tonghua Gou |
| Pig farm wastewater sterilization and disinfection method | CN107500397A | 2017 | 3 | Method | China | Dingfa Iu, Kai Liu, Jun Jiang, Qintie Lin, Hongli Zhou |
| Method and special equipment for removing antibiotic-resistant bacteria and resistant genes in sewage | CN111620493A | 2020 | 2 | Method and apparatus | China | Hongna Li, Zhiguo Zhang, Changxiong Zhu, Binxu Li, Jing Ye, Bing Geng, Yunlong Tian, Ping Guo, Lianfang Li |
| Treatment of fertigation water | US2020354239A1 | 2020 | 2 | Method and system | US | Serge Levesque, Thomas Graham, Dorin Bejan, Jaime Lawson, Mike Dixon, Ping Zhang |
| Wastewater composite disinfection process for harmless treatment of livestock and poultry died of illness | CN111056701A | 2020 | 2 | System | China | Jianwei Zhang, Yong Fang, Weiping Wang, Jianxiang Chen, Youqiang Yang |
| Technology for producing a disinfectant for neutralizing viruses, bacteria and other microorganisms | WO2020019047A1 | 2020 | 1 | System | Brazil | Giovannu Beccaro |
| Water treating apparatus based on advanced oxidation | KR20210042540A | 2021 | 0 | Apparatus | South Korea | Byun Seok Jong, Cha Sang Wha |

**Table SM4.** Complete systemic analysis for manuscript 2: Reduction of horizontal transfer of conjugative plasmid by UV irradiation and low-level chlorination [(Lin et al., 2016)](https://sciwheel.com/work/citation?ids=5727936&pre=&suf=&sa=0).

| **Article: Reduction in horizontal transfer of conjugative plasmid by UV irradiation and low-level chlorination** [**(Lin et al., 2016)**](https://sciwheel.com/work/citation?ids=5727936&pre=&suf=&sa=0)**.** | | | | |
| --- | --- | --- | --- | --- |
| **Lens** | **The lens is dealt?** | **If yes, how is this lens dealt with in the publication?** | **Main strengths** | **Main weaknesses** |
| Conditions and components of the disinfection system | Yes | In the methodology section they describe the disinfection systems. There are two systems, one based on UV radiation evaluating seven different frequencies and the other is based on chlorination using sodium hypochlorite evaluating six doses of free chlorine.  They specify types of co-helpers, concentrations of all reagents, exposure times, frequencies of the radiation source, and volumes. | They are simple disinfection systems to replicate. They compare two disinfection systems commonly implemented in water treatment (waste, drinking, irrigation, etc.). Evaluation with small volumes (10 mL) for which there is less reagent consumption. The parameters that vary are the most important for the two disinfection systems (UV and Cl_2_). High detail in the step by step of the methodology for the study of disinfection. They suggest low free chlorine levels (0.05 - 0.5 mg/lL, simulating residual chlorine in drinking water pipes. | It is a preliminary study focused on determining if, even after disinfection, the horizontal transfer of ARG occurs, for which reason they do not implement a greater volume. They do not explore scaling up the experiments and the continuous mode, nor is it posed as a perspective.  The suggested free chlorine levels for drinking water treatment should be evaluated in systems with conditions closer to the real ones. They do not explore other alternatives to the studied disinfectants focused on lower energy consumption, easy disposal or use (e.g. UV LEDs). |
| Physicochemical characteristics of the aqueous matrix | Yes | They implement 0.9% sterile saline water and the LB culture medium to avoid any stress on the microorganisms (*E. coli HB101* and *K12*) that may generate errors in the results, because in this study they focus on observing the ARG transfer mechanisms after disinfection. | The use of sterile saline water and the LB culture medium to avoid stress of the microorganism (*E. coli HB101* and *K12*), makes it easier to determine the disinfection mechanism and how the ARG (*aphA, tetA, tetR*, and *bla* genes) transfer mechanisms between bacteria are affected. The concentration of NaCl in the saline solution (0.9%) can react with the added sodium hypochlorite, increasing the production of dichlor (Cl_2_) which can promote the disinfection of the water. | The effect of the physicochemical characteristics of the water (waste, irrigation, or synthetic) on the elimination of the microorganism (*E. coli HB101* and *K12*) or the DNA (*aphA*, *tetA*, *tetR*, and *bla* genes) in the plants is completely unknown. specific conditions of disinfection systems. Which can negatively or positively affect the efficiency of elimination of the microorganism and DNA. |
| Inactivated microorganisms | Yes | Elimination of two bacteria to determine their interaction in the horizontal transfer of ARG. The ARG donor bacterium was *E. coli HB101* (it had the *RP4 plasmid* with the ARGs of interest) and the recipient bacterium was an *E. coli K12* strain. The specifications of its selection, obtaining and cultivation are described in the methodology. | They implement a species related to acute diarrheal diseases (ADD) as a model bacterium, a health problem associated with poor water quality and crops irrigated with this water, which is why evaluating the elimination of ARGs and the bacteria itself can give a first good approximation to the disinfection of water contaminated with this microorganism and similar pathogens. | Because it is a species under fairly controlled conditions, the effect of the physicochemical characteristics of the water (residual, irrigation, drinking or synthetic) on the elimination of bacteria is completely unknown. |
| Associated ARG removed | Yes | Indirect evaluation of the deletion of the *FlgC*, *ompF* and *TraG* genes, genes that encode proteins necessary in the horizontal transfer of genes between bacteria. The authors explain in the publication that four genes (ARG) *aphA*, *bla*, *tetA* and *tetR* associated with resistance to kanamycin, ampicillin and tetracycline, respectively, are found in the *RP4 plasmid*. These ARGs can be transferred to the recipient bacterium (rifampicin resistant due to mutations in the *rpoB* gene) through the expression of three genes *FlgC*, *ompF* and *TraG*. | Indirect monitoring of damage or disruption of the horizontal transfer of ARG by evaluating the expression of the flagellar genes (*FlgC*) encoding a flagellar protein, an outer membrane protein (*ompF*) gene, and a transfer-regulated gene (*TraG*), which together facilitate bacteria-bacteria interaction to transfer the ARGs present in the *RP4 plasmid*. This monitoring allows transporting the information to future work with other plasmids and other bacteria that implement the same mechanism of conjugative transfer. | In the publication they do not explore other mechanisms of horizontal transfer of ARG (Transformation and transduction) where the transfer of a plasmid does not occur. They do not explore direct damage to ARGs. |
| Disinfection system efficiency | Yes | Efficiency is described in figures in terms of bacterial density ratios (donor and recipient bacteria represented in CFU/mL), bacterial density at transfer frequency, and relative expression of *FlgC*, *OmpF*, and *TraG*.  They found that the transfer frequency decreased after exposure to UV doses between 5 and 20 mJ/cm^2^ (decreased from 2.75 × 10 to 2.44 × 10^-5^) and chlorine doses between 0.3 and 0. 5 mg/L (up to 4.40 × 10^−5^ or below the detection limit). | Efficiency results in the elimination of the bacteria and normalized genes. Implementation of a model of line adjustment to analyze the conjugative logarithmic transfer frequency according to UV radiation. mRNA expression levels of conjugation-related genes calculated and normalized with the 2 delta delta CT method. Arithmetic average. | Efficiencies in ratios of bacterial density (donor and recipient bacteria represented in CFU/mL), bacterial density at transfer frequency, and relative expression of *FlgC*, *OmpF*, and *TraG* are illustrated, but efficiencies are not illustrated anywhere, either. Tables with specific data on the elimination of bacteria or genes are illustrated. |
| Cost analysis of the disinfection system | No | - | - | - |

**Table SM5.** Complete systemic analysis for the manuscript 3: Mechanisms of ultraviolet disinfection and chlorination of *Escherichia coli*: Culturability, membrane permeability, metabolism, and genetic damage [(Xu et al., 2018)](https://sciwheel.com/work/citation?ids=14174369&pre=&suf=&sa=0).

| **Article: Mechanisms of ultraviolet disinfection and chlorination of *Escherichia coli*: Culturability, membrane permeability, metabolism, and genetic damage**[**(Lin et al., 2016; Xu et al., 2018)**](https://sciwheel.com/work/citation?ids=5727936,14174369&pre=&pre=&suf=&suf=&sa=0,0)**.** | | | | |
| --- | --- | --- | --- | --- |
| **Lens** | **The lens is dealt?** | **If yes, how is this lens dealt with in the publication?** | **Main strengths** | **Main weaknesses** |
| Conditions and components of the disinfection system | Yes | In the methodology section they describe disinfection systems based on UV and chlorination. In the case of UV radiation, they implemented a mercury lamp, specifying the power, intensity, and radiation dose (light intensity × exposure time), the latter varying from 4 to 400 mJ/cm of time. In the case of chlorination, they implemented a sodium hypochlorite solution to establish the desired concentrations of free chlorine between 0 and 25 mg/L. In all the experiments they kept the initial pH constant at 7.4 and the volume at 40 mL. | They are simple disinfection systems to replicate. They compare two disinfection systems commonly implemented in water treatment (waste, drinking, irrigation, etc.). Evaluation with small volumes (40 mL) for which there is less reagent consumption. The parameters that vary are the most important for the two disinfection systems. They specify disinfection times and monitoring methods for UV radiation intensity and free chlorine. | It is a preliminary study focused on determining the disinfection mechanisms involved in the UV radiation and chlorination systems, therefore, they do not implement a greater volume. They do not explore scaling up the experiments and the continuous mode, nor is it posed as a perspective. They do not explore other alternatives to the studied disinfectants focused on lower energy consumption, easy disposal or use (e.g. UV LEDs). |
| Physicochemical characteristics of the aqueous matrix | Yes | They implement a phosphate-buffered saline solution (PBS, pH 7.4) to avoid any stress on the microorganisms (*E. coli* ATCC 25922) that could generate errors in the results, because in this study they focus on observing the disinfection mechanisms involved in the UV radiation and chlorination systems. | The use of PBS to avoid the stress of the microorganism (*E. coli* ATCC 25922), makes it easier to determine the disinfection mechanism because it is one of the most common solutions in the preparation of inoculums for plate counting, monitoring by electron microscopy scanning (SEM) and flow cytometry (FCM). PBS, having concentrations of 8 g/L of NaCl, can promote the reaction with sodium hypochlorite, increasing the production of dichlor (Cl2) which can promote water disinfection. | The effect of the physicochemical characteristics of the water (residual, irrigation, or synthetic) on the elimination of the microorganism (*E. coli* ATCC 25922) under the specific conditions of the disinfection systems is completely unknown. Which can negatively or positively affect the efficiency of elimination of the microorganism and damage to the genetic material. |
| Inactivated microorganisms | Yes | They eliminated bacteria from an ATCC grade strain of the *Escherichia coli* species *(E. coli* ATCC 25922). In the methodology section, they describe how they carried out the initial culture, the culture for the disinfection experiments and the follow-up of the inactivation and damage in the genetic material by means of different techniques or methods. | The use of an ATCC strain allows to ensure the quality of the results of microbiological laboratory tests. In addition, *E. coli* facilitates carrying out a study, since it is a microorganism with a short life cycle, which allows for short-term experiments. It also reproduces rapidly and in large numbers, making it easy to obtain enough biological material for study. Another advantage of using *E. coli* is its wide availability and distribution, which allows researchers around the world to access this microorganism. Likewise, by implementing *E.coli* as a model microorganism, there is a good first approximation to the disinfection mechanisms on a pathogenic species used as a fecal indicator and that may be related to acute diarrheal diseases (ADD). | Being an ATCC grade strain, the effect of disinfection on strains or species that may be chlorine resistant or with different morphological characteristics (e.g. bacteria with capsids) is completely unknown. Because it is a species under fairly controlled conditions, the effect of the physicochemical characteristics of the water (residual, irrigation, drinking or synthetic) on the elimination of bacteria is completely unknown. |
| Associated ARG removed | No | - | - | - |
| Disinfection system efficiency | Yes | Efficiency is described in figures and images in terms of cultivability, membrane permeability, metabolism and genetic damage by observing DNA damage on the region that cofidicates for β-glucuronidase (*uida*), or, in the expression of mRNA for synthesis. Chromosomal replication initiator protein (*dnaA*), single-stranded DNA binding protein (*ssb*), damage repair mechanisms (*RecA*) and glutamic acid decarboxylase (*gadA*) production. The complete inactivation of *E.coli* was obtained with UV doses between 35 to 80 mJ/cm^2^, and, with 5 mg/L of free chlorine in contact for 10 minutes (elimination of 7 logarithmic units), where percentages of cells were observed severely damaged between 87.9 to 100%. On the other hand, after 30 minutes of exposure with 5 mg/L of free chlorine, all of the mRNA functions were lost. | Evaluation of culturability, membrane permeability and ATP levels at disinfection using the concentration of *E. coli* after disinfection (N) normalized by the initial concentration (N_0_). They implement an equation to determine the reduction of genes (DNA and mRNA). Comparison of disinfection by UV radiation and chlorination in figures of bacterial inactivation in logarithmic unit decrease, membrane integrity by fluorescence obtained, membrane permeability and metabolism in percentage, reduction of gene copies. Complementarity in the evaluation of efficiency by different terms and techniques. Arithmetic means and error bars. | No table with summary of results in % or concentrations, only information in graphs |
| Cost analysis of the disinfection system | No | - | - | - |

**Table SM6.** Complete systemic analysis for the manuscript 4: Combination of flow cytometry and molecular analysis to monitor the effect of UVC/H_2_O_2_ vs UVC/H_2_O_2_/Cu-IDS processes on pathogens and antibiotic resistance genes in secondary wastewater effluents [(Di Cesare et al., 2020)](https://sciwheel.com/work/citation?ids=14174370&pre=&suf=&sa=0).

| **Article: Combination of flow cytometry and molecular analysis to monitor the effect of UVC/H_2_O_2_ vs UVC/H_2_O_2_/Cu-IDS processes on pathogens and antibiotic resistant genes in secondary wastewater effluents** [**(Di Cesare et al., 2020)**](https://sciwheel.com/work/citation?ids=14174370&pre=&suf=&sa=0)**.** | | | | |
| --- | --- | --- | --- | --- |
| **Lens** | **The lens is dealt?** | **If yes, how is this lens dealt with in the publication?** | **Main strengths** | **Main weaknesses** |
| Conditions and components of the disinfection system | Yes | In the methodology section, they described in detail the conditions of the compared disinfection systems. The systems were: UV-C/H_2_O_2_ and UV-C/H_2_O_2_/Cu-IDS. They implement a constant intensity UV-C lamp, add H_2_O_2_ from the reagent and prepare the Cu-IDS complex. Additionally, they described the parameters established as constants: UV lamp intensity, pH, reactor volume (150 mL), initial concentration of initial H_2_O_2_ and Cu-IDS, exposure time (25 min). | By studying a modification of a traditional disinfection system, the spectrum of possibilities is broadened to determine an efficient system in the elimination of pathogens and antibiotic resistance genes (*intI1*, *tet A*, *qnrS* and *sul2*). They specify disinfection times and monitoring methods for the intensity of UV radiation and for free Cu-IDS. | They did not evaluate treating larger volumes, therefore, there are no results that facilitate the scalability of the disinfection systems. They do not explore other alternatives to the disinfectants studied, focused on the lower consumption of reagents, lower energy consumption, easy disposal or use (e.g. UV LEDs or the electrogeneration of H_2_O_2_). |
| Physicochemical characteristics of the aqueous matrix | Yes | They implemented real samples as an aqueous matrix, being wastewater treated by primary and secondary systems in a WWTP in Verbania, Italy. In the methodology section they briefly described the collection and storage of wastewater samples. Additionally, in the supplementary material they described in detail the physicochemical characteristics (pH, COD, BOD_5_, SST, N-NO_2_, N-NO_3_, N-NH4, TP and TN), and microbiological (presence of bacteria of the genera *Acinetobacter*, *Aeromonas*, *Bacillus*, *Bacteroides*, *Citrobacter*, *Enterobacter*, *Enterococcus*, *Escherichia-Shigella*, *Legionella*, *Morganella*, *Pantoea*, *Prevotella*, *Proteus*, *Pseudomonas*, *Serratia*, *Streptococcus*, and *Treponema*). | Implementation of real wastewater samples, bringing the study closer to realistic conditions for possible large-scale applications. | Being a first study in a relevant environment, they do not involve the option of studying wastewater samples from different WWTPs that implement different primary, secondary or tertiary processes (CAS, MBR, AOP, ponds, membrane, ozonation, etc.), or studying samples from different parts of the world, which can increase the scope of the study and the disinfection system. |
| Inactivated microorganisms | Yes | After analyzing the 16S rRNA gene by qPCR in the wastewater samples, they detected and decreased the relative abundance of putative pathogenic bacteria of the genera *Acinetobacter*, *Aeromonas*, *Bacillus*, *Bacteroides*, *Citrobacter*, *Enterobacter*, *Enterococcus*, *Escherichia-Shigella*, *Legionella*, *Morganella*, *Pantoea*, *Prevotella*, *Proteus*, *Pseudomonas*, *Serratia*, *Streptococcus,* and *Treponema*. In the figures (Figures 2 and 3) and the supplementary material section, they specify the initial relative abundances and at different times of contact with the disinfection systems. | They studied the elimination of a wide variety of genera of putative pathogenic bacteria, which using molecular biology techniques confirmed some genera of potential pathogenic bacteria such as *Aeromonas spp*. and *Escherichia-Shigella spp*, genera often associated with acute diarrheal disease (ADD). This would be a first approach to the disinfection of pathogenic bacteria of interest due to health problems associated with poor water quality and crops irrigated with this water. | No weaknesses |
| Associated ARG removed | Yes | Direct evaluation of the decrease in abundances of the tested genes, which were expressed as copy of ARG or copy of *intI1* per copy of the 16S rRNA gene. The genes evaluated were: *class 1 integrons* (targeting the integrase *intI1* gene), *tet A* (gene providing resistance against tetracycline), *qnrS* (against quinolones) and *sul2* (against sulfonamides) were selected as ARGs. | They evaluated the decrease in abundance of three ARG models (the *tet A* gene is involved in resistance against tetracycline, the *qnrs* gene against quinolones and *sul2* against sulfonamides). These three families of antibiotics are the three most widely implemented types of antibiotics worldwide in the treatment of a wide variety of infections (respiratory, urinary, gastric, skin, eye, reproductive systems, etc.). Resulting in a first approximation for the elimination of different ARGs that may be in different bacteria. The implementation of qPCR in the detection of ARG and the follow-up in its reduction during the disinfection process, obtaining fast results, with high specificity, precision and sensitivity, facilitating the comparison and analysis of the data obtained between the two systems. disinfection (UV-C/H_2_O_2_ and UV-C/H_2_O_2_/Cu-IDS). | They do not expand the study model genes to genes associated with resistance to other families of antibiotics such as penicillins, macrolides, aminoglycosides, carbapenems, etc., which could specify the efficiency of the disinfection systems (UV-C/H_2_O_2_ and UV-C/H_2_O_2_/Cu-IDS) and have more useful results for its implementation or scaling. |
| Disinfection system efficiency | Yes | The efficiency is described in the results and discussion sections. In addition, the efficiency can be observed in the figures, where the results obtained with the two disinfection systems were compared in terms of reduction of the total bacterial abundance in logarithmic units, bacterial viability in percentages, relative abundance of potential human pathogenic bacteria in copies of 16S rRNA, and ARG dynamics in gene copies. The efficiencies of both systems were evaluated with growth under conditions favorable to human pathogens (rich medium and 37 °C) and under environmental imitation conditions (original WW and 20 °C). The UV-C/H_2_O_2_/Cu-IDS disinfection system showed higher efficiencies in the inactivation of bacterial cells in the regrowth experiments under ambient conditions, but similar efficiencies to the UV-C/H_2_O_2_ system in the regrowth experiments under conditions favorable to human pathogens. | They implemented an experimental design that included the simulation of a 24-h regrowth under environmental imitation conditions and favorable to human pathogens, which they described in the supplementary material, to complement the vision of the efficacy of disinfection systems. All the data shown in the figures are arithmetic means of three data (biological replicates) for each investigated condition/treatment. In the use of qPCR for the detection of the genes, they implemented the mean value, the standard deviation, the R2 and the limits of quantification for all the genes studied. | Without a table with summary of results in %, concentrations or number of copies of the genes, only information in graphs. The figures do not have error bars despite implementing arithmetic means of three repetitions. |
| Cost analysis of the disinfection system | No | - | - | - |

**Table SM7.** Complete systemic analysis for the manuscript 5: Free radical-removing extracellular polymeric substances to enhance the degradation of intracellular antibiotic resistance genes in multiresistant *Pseudomonas Putida* by UV/H_2_O_2_ and UV/peroxydisulfate disinfection processes [(Meng et al., 2022)](https://sciwheel.com/work/citation?ids=14174372&pre=&suf=&sa=0).

| **Article: Free radicals removing extracellular polymeric substances to enhance the degradation of intracellular antibiotic resistance genes in multi-resistant *Pseudomonas Putida* by UV/H_2_O_2_ and UV/peroxydisulfate disinfection processes** [**(Meng et al., 2022)**](https://sciwheel.com/work/citation?ids=14174372&pre=&suf=&sa=0)**.** | | | | |
| --- | --- | --- | --- | --- |
| **Lens** | **The lens is dealt?** | **If yes, how is this lens dealt with in the publication?** | **Main strengths** | **Main weaknesses** |
| Conditions and components of the disinfection system | Yes | In the methodology section, they describe in detail the technical components and parameters controlled in each disinfection system. In this paper they compare three disinfection systems: UV, UV/H_2_O_2_ and UV/peroxydisulfate (PDS). In the systems they implemented a 254 nm lamp and 0.3 mM H_2_O_2_ or PDS. The constant parameters in the systems were: The dimensions of the reactor (Petri dish), the distance between the lamp and the reactor, the UV fluence (the exposure time (seconds) multiplied by the photon fluence rate) and the volume. to treat (100 mL). The pH was the only parameter that varied (pH 3.0, 7.0 and 10.0) evaluating the effect of pH on the efficiency of cell membrane damage and the decrease in the relative abundance of ARGs. | With the study of the efficiency in the elimination of microorganisms and ARG (iARG and eARG) with different disinfection systems, they present a wide spectrum of possibilities to determine the most efficient system and with optimal conditions for its future scaling. Comparisons of removal efficiencies at different pHs would facilitate determining use conditions of the systems close to real environmental conditions, mitigating the change in pH in the water. | The reactor is limited to a Petri dish with a capacity of 100 mL, they did not evaluate treating larger volumes, therefore, there are no results that facilitate the scalability of the disinfection systems. They do not explore other alternatives to the disinfectants studied, focused on the lower consumption of reagents, lower energy consumption, easy disposal or use (e.g. UV LEDs or the electrogeneration of H_2_O_2_). |
| Physicochemical characteristics of the aqueous matrix | Yes | As aqueous matrices, they implemented sterile water and real wastewater to determine the effect of the matrix on the elimination of microorganisms and ARGs (iARG and eARG). The wastewater sample was collected from a secondary effluent from a WWTP in Taihu New City, China. WWTP used an anaerobic-anoxic-oxic process as secondary treatment. In the methodology section they describe the physicochemical parameters of real wastewater such as pH (6.4), TOC, DO, COD and conductivity. The pH variation experiments were carried out with sterile water. The experiments with the real water sample had no change in pH. | Implementation of real wastewater samples, bringing the study closer to realistic conditions for possible large-scale applications. They did not modify the initial parameters of the real wastewater, such as the pH, thus maintaining the evaluation of the efficiency in the elimination of microorganisms and ARG (iARG and eARG) in relevant/close to real conditions. | Being a first study in a relevant environment, they do not involve the option of studying wastewater samples from different WWTPs that implement different primary, secondary and/or tertiary processes (CAS, MBR, AOP, ponds, membrane, ozonation, etc.), or study samples from different parts of the world, which can increase the scope of the study and the disinfection system. |
| Inactivated microorganisms | Yes | Elimination of the species *Pseudomonas putida* (*P. putida*), specifically *P. putida MX-2* resistant to multiple drugs (it has seven ARGs). In the introduction they described why it was chosen as a model bacterium, specifying that it is an opportunistic pathogen present in soil and water, it has a cell envelope that protects it during disinfection with UV radiation, and it is a key pathogen in the spread of ARG to other human pathogens, and its outbreak in hospital drinking water can cause nosocomial infections. | They implement a model bacterium that is highly pathogenic and dangerous, multi-resistant (resistant to five different families of antibiotics), generating health problems due to poor water quality and crops irrigated with this water, for which they evaluate the elimination of the bacterium *P. putida* and associated ARGs (iARG and eARG) can give a first approximation to the disinfection of water contaminated with this microorganism or bacilli with similar characteristics. | They miss the opportunity to eliminate a greater variety of pathogenic bacteria present in wastewater (*E. coli*, *P. aeruginosa*, *A. Fischeri*, *Campylobacter*, *Salmonella spp*., *Shigella dysenteriae* or others, due to the great diversity in the microbial community), because they focus on a single species (*P. putida*). |
| Associated ARG removed | Yes | Removal of iARG and eARG. In the model bacterium that they implement (*P. putida MX-2*) they determined that it carried three ARGs that encode plasmids (eARG), which are *tetA -01* (provides resistance to tetracycline), *aac6-lb* and *strA* (provides resistance to aminoglycosides). in addition to four ARGs that encode chromosomes (iARGs) that are *acrB* (provides resistance to fluoroquinolones), *tetA -02* (provides resistance to tetracycline), *sulI* (provides resistance to sulfonamides) and *mexF* (provides resistance to penicillins). | Elimination of iARGs was more difficult than elimination of eARGs or *P. putida* bacteria. They eliminate a wide variety of ARGs that can generate resistance to five families of antibiotics (tetracycline, aminoglycosides, fluoroquinolones, sulfonamides, and penicillins). They present the removal of iARGs in terms of degradation kinetics of total iARGs transported, facilitating comparison of the normalized data. They evaluate the removal of both iARGs and eARGs together, highlighting the potential disinfection mechanisms involved in removing each type of ARG. In turn, they include the implementation of molecular biology techniques such as qPCR for the analysis of alternative sequences in the bacterial chromosome and plasmids. Thus, it is an ARG elimination study with important conceptual and methodological bases for future studies. | No weaknesses |
| Disinfection system efficiency | Yes | The efficiency is described in the results and discussion section, figures and images are included to illustrate the efficiency in terms of inactivation of *P. putida MX-2*, degradation of the transported i-ARGs, changes in cell membrane damage and the degradation of bacterial extracellular polymeric substances (EPS) from *P. putida MX-2*. In addition, they compare efficiencies of three disinfection systems (UV, UV/H_2_O_2_ and UV/PDS). The highest efficiencies in the inactivation of *P. putida MX-2* (decrease of 7 logarithmic units), generation of cell membrane damage (between 70 to 86% of cells with membrane damage), ARG degradation (decrease of 4 logarithmic units) and EPS (76.2% - 84.8% of the polysaccharides and 51.2% - 56.7% of the proteins in the EPS) were obtained with the UV/H_2_O_2_ and UV/PDS systems with the following operating conditions: UV light of 254 nm with a confluence of 320 mW/cm^2^, 0.3 mM of disinfectant (H_2_O_2_ or PDS) at any of the pH's tested (3, 7 or 10). | They carried out an in-depth statistical analysis of the results obtained in all terms (inactivation of *P. putida MX-2*, generation of damage to the cell membrane, degradation of ARG and EPS. To find the rate constants in the inactivation of *P. putida MX-2* and ARG degradation performed linear regressions of the log concentration of *P. putida MX-2* or transported ARGs against UV fluence data, performed Pearson correlation analysis and tests of significance. They were analyzed by one-way ANOVA with a Student-Newman-Keuls (SNK) test. For all the analyzes they used a significance limit of α = 0.05. Arithmetic means and error bars since they performed all the experiments in triplicate. In the supplementary material they expand the efficiency results and the respective statistical analyzes in tables. | No weaknesses |
| Cost analysis of the disinfection system | No | - | - | - |

**Table SM8.** Complete systemic analysis for Patent 2: Method and specialized equipment for removing antibiotic-resistant bacteria and resistance genes from wastewater (CN111620493A).

| **Patent: Method and special equipment for removing antibiotic-resistant bacteria and resistant genes in sewage (CN111620493A).** | | | | |
| --- | --- | --- | --- | --- |
| **Lens** | **The lens is dealt?** | **If yes, how is this lens dealt with in the publication?** | **Main strengths** | **Main weaknesses** |
| Conditions and components of the disinfection system | Yes | The disinfection system consists of a combination of four technologies: electrochemical reaction, ultraviolet disinfection, ultrasonic treatment and H_2_O_2_ disinfection. The technical components of the device include a reaction vessel with water inlet and outlet, at least one pair of anode and cathode electrodes at the bottom, at least one ultrasonic element, and at least one ultraviolet light source at the top of the device. bowl. The system can also include a feed port and a stirring element in the reaction vessel.  Regarding the controlled or varied parameters in the system, several characteristics are mentioned. The current density in electrochemical reaction should be 10 mA/cm^2^ to 40 mA/cm^2^, the ultraviolet radiation dose in ultraviolet disinfection should be 20 mJ/cm^2^ to 80 mJ/cm^2^, the ultrasonic radiation frequency in the ultrasonic treatment should be between 20kHz and 40kHz, and the H_2_O_2_ concentration in the sewage should be 10 mg/L to 40 mg/L. The duration of treatment of joints is 5 - 20 minutes. | A major strength of the patent is that it is a combined system that uses multiple technologies to eliminate antibiotic-resistant bacteria and resistance genes in wastewater. Using multiple technologies together increases the efficiency of the system compared to using just one technology. In addition, the specific parameters of each technology have been clearly defined, allowing optimization of the system in terms of effectiveness and efficiency. | A weakness of the patent could be that it does not provide detailed information on the interaction between the different technologies. It is not clear how electrochemical reaction, ultraviolet disinfection, ultrasonic treatment and H_2_O_2_ disinfection combine to achieve effective removal of antibiotic resistant bacteria and resistance genes in wastewater. Also, although specific parameters are mentioned for each technology, no information is provided on how these parameters are optimized together to achieve maximum system efficiency. They do not explore other alternatives to the disinfectants studied, focused on the lower consumption of reagents, lower energy consumption, easy disposal or use (e.g. UV LEDs). |
| Physicochemical characteristics of the aqueous matrix | No | - | - | - |
| Inactivated microorganisms | No | - | - | - |
| Associated ARG removed | Yes | In the detailed description section, they recorded the ARGs they eliminated during water disinfection, eliminating tetA, *tetC*, *tetM*, *tetW*, *tetX*: tetracycline resistance genes. And *sul1* and *sul2*: sulfonamide resistance genes. | The strength of the patent is the elimination of antibiotic resistance genes that are common in the health and food industry, and that represent a threat to public health due to the possibility of being transmitted to pathogenic bacteria. The efficiency of electrochemical technology to remove these genes is high, with removal rates greater than 90% in various treatments.  In addition, electrochemical technology is a sustainable and environmentally friendly method, since it does not use aggressive chemicals and does not generate toxic waste. | A weakness of the patent could be its limitation to the removal of specific antibiotic resistance genes, since there are many other antibiotic resistance genes that might not be affected by this technology. In addition, the patent focuses on the elimination of tetracycline and sulfamethoxazole resistance genes, leaving out other types of antibiotics and their corresponding resistance genes.  Another limitation is that the study was conducted under laboratory conditions and further research would be needed to determine its effectiveness in real-world situations, such as wastewater treatment or disinfection of medical equipment. They do not specify the molecular biological techniques or methods that they implement for monitoring. |
| Disinfection system efficiency | Yes | In the detailed description section, they specify that the disinfection system may vary in combinations between the systems described in the claims, in this case the efficiencies may vary. They record that the disinfection efficiencies of the water vary depending on the treatment used, it can range between 32.64% and 99.30% in systems with a single disinfectant such as UV or H_2_O_2_, while treatments with a combination of disinfectants UV/H_2_O_2_ reach 100% efficiency in 10 minutes of treatment. The most efficient treatment is which uses an H_2_O_2_ concentration of 40 mg/L. In general, the disinfection efficiency is higher for sulfa resistance genes than for tetracycline resistance genes. | The patent presents a large amount of data on the efficiency of different treatments in eliminating antibiotic resistance genes. The patent also provides details about the specific genes that were studied and the treatments used. Furthermore, the technical formula used to calculate the sterilization efficiency is clear and easy to understand. | The patent does not provide information about the type of bacteria that were used in the experiments, which could affect the applicability of the results to other types of bacteria. In addition, information on the frequency of resistance genes in bacteria before and after treatments is not provided, which could be useful to assess the efficacy of treatment in removing resistance genes. In addition, no statistical tests are presented to assess the significance of the differences between treatments, making it difficult to assess the certainty of the observed differences. They do not present the efficiency in figures or tables. |
| Cost analysis of the disinfection system | No | - | - | - |

**Table SM9.** Complete systemic analysis for Patent 3: Wastewater composite disinfection process for the safe treatment of diseased livestock and poultry (CN111056701A).

| **Patent: Wastewater composite disinfection process for harmless treatment of livestock and poultry died of illness (CN111056701A).** | | | | |
| --- | --- | --- | --- | --- |
| **Lens** | **The lens is dealt?** | **If yes, how is this lens dealt with in the publication?** | **Main strengths** | **Main weaknesses** |
| Conditions and components of the disinfection system | Yes | In the claims section, they specify that the wastewater disinfection system for the harmless treatment of sick and dead livestock and birds consists of several technical components, including an oil separation pretreatment, A_2_O, and AO secondary biological treatment, an advanced treatment of flocculation, sedimentation and sand filtration, use ultraviolet light irradiation with wavelengths of 253.7 nm and 184.9 nm, continuous addition of NaClO to ensure that the residual chlorine is not less than 0.5 mg/L. Parameters that are controlled or varied in the system include the maximum design dose of the ultraviolet light source (not less than 100 mJ/cm^2^), the concentration of the NaClO stock solution used (from 10 % and a concentration ratio of 3 %), and the quality parameters of the treated water, such as the concentration of fecal coliform bacteria, COD, ammoniacal nitrogen, turbidity and pH. | The system uses several treatment steps to ensure that the wastewater is fully treated and disinfected, including oil separation, secondary biological treatment, flocculation, sedimentation and sand filtration, and ultraviolet light irradiation. The system uses a combination of UV light wavelengths to ensure thorough disinfection, which is more effective than using just one wavelength. The continuous addition of NaClO guarantees that the disinfection is maintained over time and that new infections do not occur. The system complies with water quality standards, ensuring that the treated water is safe for use. | Flow rates of treated water are not mentioned, which could affect the effectiveness of the system. Specific details about the size of the reactor and the relationship between the size of the reactor and the volume of water treated, which could affect the efficiency of the system, are not mentioned. The patent does not provide information on the cost of the system and its maintenance, which could limit its use in areas with limited resources. The patent focuses on the treatment of wastewater from sick and dead livestock and poultry, which could limit its application in other contexts. |
| Physicochemical characteristics of the aqueous matrix | Yes | In the background technique and detailed description of the invention section, they describe some physicochemical characteristics of the water they treat. Being wastewater from the project for the harmless disposal of sick and dead livestock and poultry, and specifying the range of fecal coliforms, COD, ammoniacal nitrogen, and turbidity. | Implementation of real wastewater samples associated with a real problem, which increases your commercial reach. In addition, the registered physicochemical characteristics may be sufficient, since they are the characteristics that can most affect the efficiency of water disinfection through the system. | The specification of these parameters and the unique type of water can limit the commercial scope and application of the patent. |
| Inactivated microorganisms | No | - | - | - |
| Associated ARG removed | No | - | - | - |
| Disinfection system efficiency | Yes | In the summary section of the invention, they describe that with the disinfection system described in lens 1 they achieved an efficient elimination of fecal coliform bacteria in a range of 1.7X10^4^ to 2.2X10^4^ MPN/L, as well as a COD reduction. at a level equal to or less than 100 mg/L, ammonia nitrogen at a level equal to or less than 100 mg/L, turbidity at a level of 2 to 3 NTU, and maintaining a pH between 6 and 8. These are the system conditions under which these efficiencies were achieved. | They have several strengths in terms of the efficiency of the disinfection process. Firstly, the elimination of bacteria is achieved through a synergistic effect, combining different disinfection methods to maximize effectiveness. Second, the process is capable of effectively removing residual refractory organic matter, color, odor, etc. in the water, ensuring continuous disinfection and better water quality. Furthermore, the patent highlights that the process is not simply an overlap of processes, but rather a synergistic effect, suggesting that the efficacy is greater than the sum of the effects of the individual processes. | Although the patent has several strengths in terms of the efficiency of the disinfection process, there are also some weaknesses that must be taken into account. First, the patent does not provide detailed information on the amount of testing done to prove the effectiveness of the process. The number of samples taken or the number of repetitions performed are not indicated. This makes it difficult to assess the reliability of the results and their statistical representativeness. Second, although the patent mentions that the process is capable of effectively removing residual refractory organic matter, no information is provided on the reduction of the concentration of other contaminants. Therefore, the overall effectiveness of the process for the removal of contaminants in the wastewater cannot be determined. Third, although the patent mentions that the process is capable of compensating for UV rays that only propagate along a straight line, no information is provided on how this is achieved. More information would be needed to assess the effectiveness of this compensation. They do not record figures or tables, they do not add statistical analysis of the only information on the elimination of coliform bacteria. |
| Cost analysis of the disinfection system | No | - | - | - |

**Table SM10.** Full systemic analysis for Patent 4: Technology for producing a disinfectant for neutralizing viruses, bacteria and other microorganisms (WO2020019047A1).

| **Patent: Technology for producing a disinfectant for neutralizing viruses, bacteria and other microorganisms (WO2020019047A1).** | | | | |
| --- | --- | --- | --- | --- |
| **Lens** | **The lens is dealt?** | **If yes, how is this lens dealt with in the publication?** | **Main strengths** | **Main weaknesses** |
| Conditions and components of the disinfection system | Yes | In the claims section, they specified the conditions and components of the disinfection system. The technical components of the disinfection system include a cartridge filter, a centrifugal pump, a flow reader, a flow initiator valve, a brine pump, a magnetic brine level pump, a conductivity meter, a reactor, an oxidizing reaction reducer, product storage and an electrical panel. The system works through the electrochemical synthesis of hypochlorous acid as an active principle, which is produced via electrolysis and through a saline solution produced in situ before the water disinfection system. A strictly neutral pH is controlled during the hypochlorous acid production process, and a myeloperoxidase (MPO) enzyme is used to produce the biocidal agent from hydrogen peroxide (H_2_O_2_) and chloride ions (CI^-^). | The strengths of the patent are the use of an electrochemical method to produce hypochlorous acid as an active ingredient, which eliminates the need to use dangerous chemicals. In addition, the disinfection system works under a strictly neutral pH, which minimizes the impact on the quality of the treated water. A myeloperoxidase (MPO) enzyme is also used to produce the biocidal agent from hydrogen peroxide (H_2_O_2_) and chloride ions (CI^-^), minimizing the generation of toxic byproducts. | The weaknesses of the patent may include the need for a complex set of technical components for the production of hypochlorous acid, which may increase the cost of implementing the disinfection system. Furthermore, no specific information is provided on the parameters that are controlled or varied in the disinfection system, which makes it difficult to assess the effectiveness of the system under different operating conditions. |
| Physicochemical characteristics of the aqueous matrix | No | - | - | - |
| Inactivated microorganisms | Yes | In the section of the patent description, it indicates that the disinfection system acts effectively against bacteria, viruses, fungi, spores and pathogenic microorganisms most commonly spread in the circuit water, including *Campylobacter spp.*, *Escherichia coli*, *Legionella pneumophila*, *Pseudomonas aeruginosa*, *Salmonella*, *Staphylococcus aureus*, *Aspergillus niger*, *Candida albicans*, among others. | The patent is capable of eliminating a wide range of bacteria, viruses, fungi, and spores, including species such as *Campylobacter spp.*, *Escherichia coli*, *Legionella pneumophila*, *Pseudomonas aeruginosa*, *Salmonella*, and *Staphylococcus aureus*, among others. This is important because many of these species can cause serious disease in humans and animals, so their removal is crucial to maintaining public health and food safety. The solution used in the patent has a high disinfection power, even in the presence of biofilm, which means that it can penetrate the cell membrane of the target microorganism and eliminate it effectively. This is especially important for species like *Legionella pneumophila*, which can form biofilms in pipes and water systems, making them difficult to remove. This can reduce environmental impact and lower water treatment costs. The solution used in the patent does not alter the characteristics of the treated drinking water and does not produce chlorinated by-products that are harmful to health. This is important because some chemicals used in water disinfection can adversely affect the taste, odor, and quality of drinking water, and can produce toxic byproducts that are harmful to health and the environment. | The main weakness of the patent is that it focuses solely on the elimination of bacteria, viruses, fungi, spores and pathogenic microorganisms in the water of the circuit. This means that it does not address other possible contaminants, especially ARGs that may be present in real water and can be released during the disinfection process, it does not specify anything about resistant bacteria (ARB), as well as other chemical contaminants. |
| Associated ARG removed | No | - | - | - |
| Disinfection system efficiency | Yes | In the patent description section, they highlight that they are highly effective against a wide range of bacteria, viruses, fungi, and spores, and they even mention that the disinfection system has strong disinfection power even in the presence of biofilm, since that penetrates the cell membrane of the target microorganism. | They register high efficacy against a wide range of bacteria, viruses, fungi and spores, which means they can be effective against a variety of common pathogenic microorganisms in loop water. The strong disinfection power even in the presence of biofilm, which means that it can penetrate the cell membrane of the target microorganism and kill it, even if it is surrounded by a protective layer. It does not alter the characteristics of the treated drinking water and provides more guarantees on eventual problems of possible corrosion of the neutral "pH" cleaning metallic conduits, which means that it is safe and has no negative effects on the treated water. Results comparable to those obtained with hypochlorite, using lower concentrations ("HOCI" is about 80 times more effective than "NaClO"), describe that they have a dilute solution, which means that it can be equally effective as other disinfectant solutions, but with lower concentrations and less environmental impact. It does not lead to the formation of harmful chlorinated by-products, which means that it is safer and has no negative effects on human health. | No quantitative information is provided on the actual effectiveness of the solution. Therefore, it is difficult to assess the true effectiveness of the solution and compare it with other disinfection solutions. Furthermore, the exact concentrations of hypochlorous acid and neutral pH needed to achieve the desired efficacy are not specified. This could make the solution difficult to reproduce and limit its practical application in different contexts. Another weakness is that the patent does not mention any environmental impact assessment of the solution. Hypochlorous acid is a strong oxidizer and may be toxic to some aquatic organisms and other beneficial microorganisms. Therefore, it is important to consider the possible side effects and the necessary safety precautions for its use. In addition, a statistical analysis is not presented, the significance of the differences between the treatments, which makes it difficult to assess the certainty of the observed differences. They do not present the efficiency in figures or tables. |
| Cost analysis of the disinfection system | No | - | - | - |

**Table SM11.** Complete systemic analysis for Patent 5: Water treatment apparatus based on advanced oxidation (KR20210042540A).

| **Patent: Water treating apparatus based on advanced oxidation (KR20210042540A).** | | | | |
| --- | --- | --- | --- | --- |
| **Lens** | **The lens is dealt?** | **If yes, how is this lens dealt with in the publication?** | **Main strengths** | **Main weaknesses** |
| Conditions and components of the disinfection system | Yes | In the original claims section, they specify the conditions and components of the disinfection system. The patent describes a water treatment system based on advanced oxidation consisting of a water supply pipe to be treated, a hydrogen peroxide (H_2_O_2_) injection unit to inject hydrogen peroxide (H_2_O_2_) into the water supply pipe to be treated, a connected line mixer to the treatment water supply pipeline for passing treatment water supplied from the treatment water supply pipeline, ozone (O_3_) bubble water injection unit for injecting ozone (O_3_) bubble water into the line mixer, a primary reaction unit connected to the line mixer and into which the treated water passing through the line mixer is introduced, a second reaction unit connected to the first reaction unit to receive the water to be treated discharged from the first reaction unit reaction and installed with a plurality of ultraviolet lamps, and a control unit for selectively performing the single treatment mode or a complex treatment in which two or more of the waters are in contact with the water to be treated.  Parameters that are controlled or varied in the system include the amount of hydrogen peroxide (H_2_O_2_) injected, the amount of ozone (O_3_) bubble water injected, the intensity of the ultraviolet lamps, the selected treatment mode (single or complex), and the flow rate of water through the system. | They have the ability to perform selective advanced oxidation treatment using a combination of ozone (O_3_), ultraviolet light, and hydrogen peroxide (H_2_O_2_), which can provide greater efficiency in removing contaminants from water. Furthermore, the system can be controlled and adjusted to adapt to different types of water and levels of contamination. | A potential weakness of the system described in the patent is that the concentrations or doses of the disinfectants (UV, H_2_O_2_ and O_3_) are not specified, nor do they present the levels of efficiency in the elimination of contaminants from the water in different situations, such as contamination levels specific or specific types of pollutants. Also, there is no mention of the possibility of generating toxic by-products through the advanced oxidation process, which can be a problem in some cases. Finally, the system's ability to handle large volumes of water is not specified, which may limit its usefulness in some industrial settings. They do not explore other alternatives to the disinfectants studied, focused on the lower consumption of reagents, lower energy consumption, easy disposal or use (e.g. UV LEDs). |
| Physicochemical characteristics of the aqueous matrix | No | - | - | - |
| Inactivated microorganisms | No | - | - | - |
| Associated ARG removed | No | - | - | - |
| Disinfection system efficiency | No | - | - | - |
| Cost analysis of the disinfection system | No | - | - | - |

**Supplementary Figures**

**A**


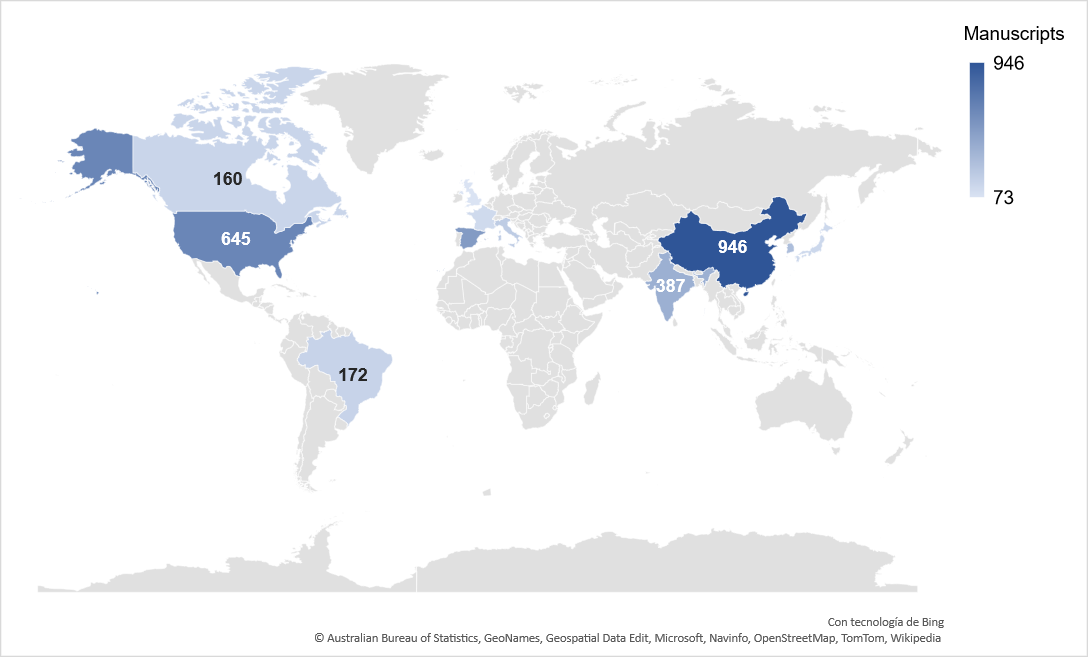


**B**


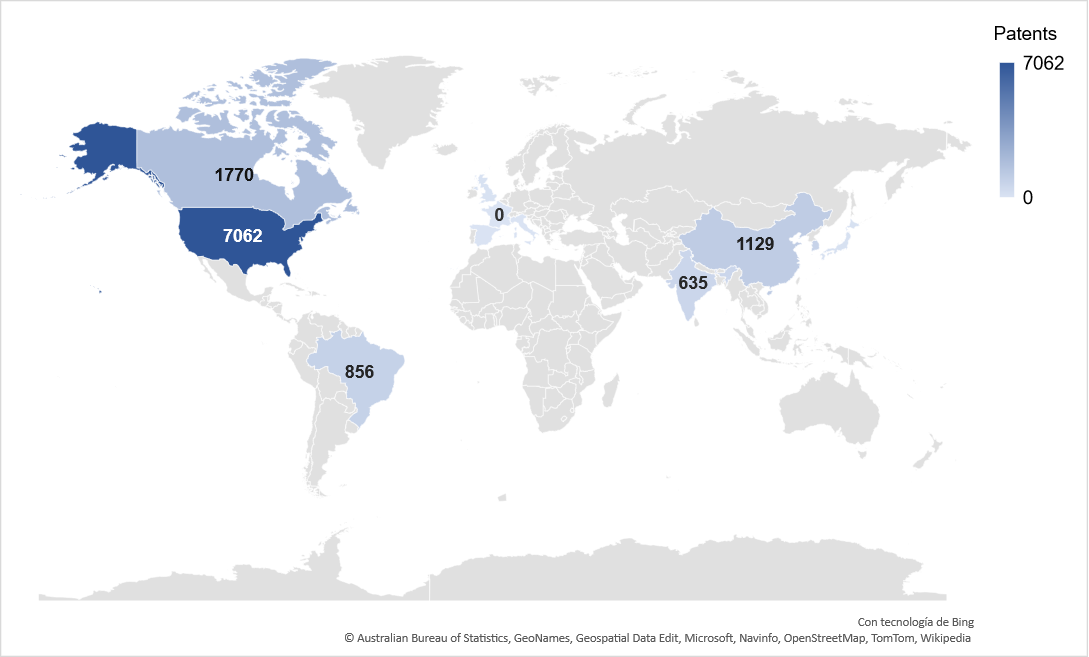


**Figure SM1.** Map showing the distribution of the number of documents in the period 2011 - 2022. **A.** Manuscripts according to the Science Direct database and **B.** Patents according to The Lens database.


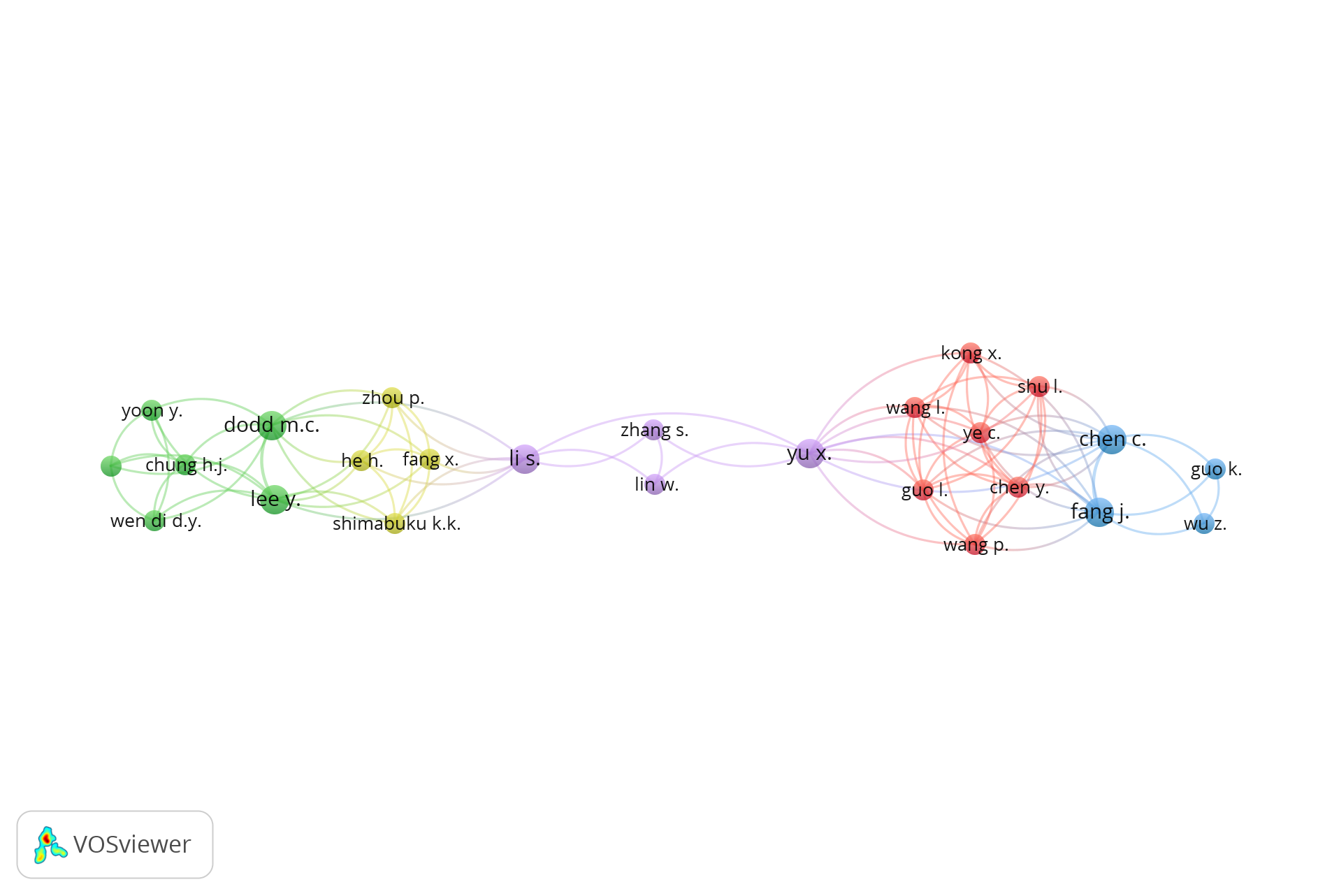


**Figure SM2**. Map of the main authors of manuscripts related to the subject of study.

**Supplementary References**

Aguilar, S., Rosado, D., Moreno-Andrés, J., Cartuche, L., Cruz, D., Acevedo-Merino, A., & Nebot, E. (2018). Inactivation of a wild isolated Klebsiella pneumoniae by photo-chemical processes: UV-C, UV-C/H2O2 and UV-C/H2O2/Fe3+. Catalysis today, 313, 94–99.

Beretsou, V. G., Michael-Kordatou, I., Michael, C., Santoro, D., El-Halwagy, M., Jäger, T., Besselink, H., et al. (2020). A chemical, microbiological and (eco)toxicological scheme to understand the efficiency of UV-C/H2O2 oxidation on antibiotic-related microcontaminants in treated urban wastewater. The Science of the Total Environment, 744, 140835.

Das, D., Bordoloi, A., Achary, M. P., Caldwell, D. J., & Suri, R. P. S. (2022). Degradation and inactivation of chromosomal and plasmid encoded resistance genes/ARBs and the impact of different matrices on UV and UV/H2O2 based advanced oxidation process. The Science of the Total Environment, 833, 155205.

Di Cesare, A., De Carluccio, M., Eckert, E. M., Fontaneto, D., Fiorentino, A., Corno, G., Prete, P., et al. (2020). Combination of flow cytometry and molecular analysis to monitor the effect of UVC/H2O2 vs UVC/H2O2/Cu-IDS processes on pathogens and antibiotic resistant genes in secondary wastewater effluents. Water Research, 184, 116194.

Ferro, G., Guarino, F., Cicatelli, A., & Rizzo, L. (2017). β-lactams resistance gene quantification in an antibiotic resistant Escherichia coli water suspension treated by advanced oxidation with UV/H2O2. Journal of hazardous materials, 323(Pt A), 426–433.

Fiorentino, A., Ferro, G., Alferez, M. C., Polo-López, M. I., Fernández-Ibañez, P., & Rizzo, L. (2015). Inactivation and regrowth of multidrug resistant bacteria in urban wastewater after disinfection by solar-driven and chlorination processes. Journal of Photochemistry and Photobiology. B, Biology, 148, 43–50.

Friedline, A., Zachariah, M., Middaugh, A., Heiser, M., Khanna, N., Vaishampayan, P., & Rice, C. V. (2015). Sterilization of hydrogen peroxide resistant bacterial spores with stabilized chlorine dioxide. AMB Express, 5, 24.

Guo, K., Wu, Z., Chen, C., & Fang, J. (2022). UV/Chlorine Process: An Efficient Advanced Oxidation Process with Multiple Radicals and Functions in Water Treatment. Accounts of Chemical Research, 55(3), 286–297.

Guo, S., Huang, R., & Chen, H. (2017). Application of water-assisted ultraviolet light in combination of chlorine and hydrogen peroxide to inactivate Salmonella on fresh produce. International Journal of Food Microbiology, 257, 101–109.

He, H., Zhou, P., Shimabuku, K. K., Fang, X., Li, S., Lee, Y., & Dodd, M. C. (2019). Degradation and Deactivation of Bacterial Antibiotic Resistance Genes during Exposure to Free Chlorine, Monochloramine, Chlorine Dioxide, Ozone, Ultraviolet Light, and Hydroxyl Radical. Environmental Science & Technology, 53(4), 2013–2026.

Huo, L., Zhao, S., Shi, B., Wang, H., & He, S. (2021). Bacterial community change and antibiotic resistance promotion after exposure to sulfadiazine and the role of UV/H2O2-GAC treatment. Chemosphere, 283, 131214.

Kang, J.-W., Kim, S.-S., & Kang, D.-H. (2018). Inactivation dynamics of 222 nm krypton-chlorine excilamp irradiation on Gram-positive and Gram-negative foodborne pathogenic bacteria. Food research international (Ottawa, Ont.), 109, 325–333.

Lin, W., Li, S., Zhang, S., & Yu, X. (2016). Reduction in horizontal transfer of conjugative plasmid by UV irradiation and low-level chlorination. Water Research, 91, 331–338.

Matin, A. R., Yousefzadeh, S., Ahmadi, E., Mahvi, A., Alimohammadi, M., Aslani, H., & Nabizadeh, R. (2018). A comparative study of the disinfection efficacy of H2O2/ferrate and UV/H2O2/ferrate processes on inactivation of Bacillus subtilis spores by response surface methodology for modeling and optimization. Food and Chemical Toxicology, 116(Pt B), 129–137.

Meng, X., Li, F., Yi, L., Dieketseng, M. Y., Wang, X., Zhou, L., & Zheng, G. (2022). Free radicals removing extracellular polymeric substances to enhance the degradation of intracellular antibiotic resistance genes in multi-resistant Pseudomonas Putida by UV/H2O2 and UV/peroxydisulfate disinfection processes. Journal of hazardous materials, 430, 128502.

Mohammadi, S., Moussavi, G., Yaghmaeian, K., & Giannakis, S. (2022). Development of a percarbonate-enhanced Vacuum UV process for simultaneous fluoroquinolone antibiotics removal and fecal bacteria inactivation under a continuous flow mode of operation. Chemical Engineering Journal, 431, 134064.

Núñez-Núñez, C. M., Chairez-Hernández, I., García-Roig, M., García-Prieto, J. C., Melgoza-Alemán, R. M., & Proal-Nájera, J. B. (2018). UV-C/H2O2 heterogeneous photocatalytic inactivation of coliforms in municipal wastewater in a TiO2/SiO2 fixed bed reactor: a kinetic and statistical approach. Reaction Kinetics, Mechanisms and Catalysis, 125(2), 1159–1177.

de Oliveira, E. F., Tikekar, R., & Nitin, N. (2018). Combination of aerosolized curcumin and UV-A light for the inactivation of bacteria on fresh produce surfaces. Food research international (Ottawa, Ont.), 114, 133–139.

Rattanakul, S., & Oguma, K. (2017). Analysis of hydroxyl radicals and inactivation mechanisms of bacteriophage MS2 in response to a simultaneous application of UV and chlorine. Environmental Science & Technology, 51(1), 455–462.

Rodríguez-Chueca, J., Ormad, M. P., Mosteo, R., & Ovelleiro, J. L. (2015). Kinetic modeling of Escherichia coli and Enterococcus sp. inactivation in wastewater treatment by photo-Fenton and H2O2/UV–vis processes. Chemical engineering science, 138, 730–740.

Sun, J., Bu, L., Deng, L., Shi, Z., & Zhou, S. (2018). Removal of Microcystis aeruginosa by UV/chlorine process: Inactivation mechanism and microcystins degradation. Chemical Engineering Journal, 349, 408–415.

Sun, P., Tyree, C., & Huang, C.-H. (2016). Inactivation of Escherichia coli, Bacteriophage MS2, and Bacillus Spores under UV/H2O2 and UV/Peroxydisulfate Advanced Disinfection Conditions. Environmental Science & Technology, 50(8), 4448–4458.

Wallace, R. L., Ouellette, M., & Jean, J. (2019). Effect of UV-C light or hydrogen peroxide wipes on the inactivation of methicillin-resistant Staphylococcus aureus, Clostridium difficile spores and norovirus surrogate. Journal of Applied Microbiology, 127(2), 586–597.

Wang, L., Ye, C., Guo, L., Chen, C., Kong, X., Chen, Y., Shu, L., et al. (2021). Assessment of the UV/Chlorine Process in the Disinfection of Pseudomonas aeruginosa: Efficiency and Mechanism. Environmental Science & Technology, 55(13), 9221–9230.

Xu, L., Zhang, C., Xu, P., & Wang, X. C. (2018). Mechanisms of ultraviolet disinfection and chlorination of Escherichia coli: Culturability, membrane permeability, metabolism, and genetic damage. Journal of environmental sciences (China), 65, 356–366.

Yoon, Y., Chung, H. J., Wen Di, D. Y., Dodd, M. C., Hur, H.-G., & Lee, Y. (2017). Inactivation efficiency of plasmid-encoded antibiotic resistance genes during water treatment with chlorine, UV, and UV/H2O2. Water Research, 123, 783–793.

Zeng, F., Cao, S., Jin, W., Zhou, X., Ding, W., Tu, R., Han, S.-F., et al. (2020). Inactivation of chlorine-resistant bacterial spores in drinking water using UV irradiation, UV/Hydrogen peroxide and UV/Peroxymonosulfate: Efficiency and mechanism. Journal of Cleaner Production, 243, 118666.

Zhang, T., Wang, T., Mejia-Tickner, B., Kissel, J., Xie, X., & Huang, C.-H. (2020). Inactivation of Bacteria by Peracetic Acid Combined with Ultraviolet Irradiation: Mechanism and Optimization. Environmental Science & Technology, 54(15), 9652–9661.

Zhang, Yiqing, Zhou, L., Zhang, Y., & Tan, C. (2014). Inactivation of Bacillus subtilis spores using various combinations of ultraviolet treatment with addition of hydrogen peroxide. Photochemistry and Photobiology, 90(3), 609–614.

Zhang, Yongji, Zhang, Y., Zhou, L., & Tan, C. (2014). Factors affecting UV/H2O2 inactivation of Bacillus atrophaeus spores in drinking water. Journal of Photochemistry and Photobiology. B, Biology, 134, 9–15.
